# Supplementary material for: The Advantages of Non-Adhesive Gel-like Embolic Materials in the Endovascular Treatment of Benign Hypervascularized Lesions of the Head and Neck
Source: Gels. 2023 Dec 4;9(12):954. doi: 10.3390/gels9120954 (PMC10742558; doi:10.3390/gels9120954)
Supplement: Supplementary file 1 [file gels-09-00954-s001.zip › gels-2691791-supplementary.pdf]

Article

The Advantages of Non-Adhesive Gel-Like Embolic Materials in the Endovascular Treatment of Benign Hypervascularized Lesions of the Head and Neck

Andrey Petrov [1,2,*], Arkady Ivanov [1,2], Egor Kolomin [1], Nikita Tukanov [1], Anna Petrova [1], Larisa Rozhchenko [1] and Julia Suvorova [1,2]

[1] Vascular Neurosurgery Department, Polenov Neurosurgical Research Institute, Branch of Almazov National Medical Research Centre, 191014 Saint Petersburg, Russia
[2] North-Western District Scientific and Clinical Center Named after L. G. Sokolov Federal Medical and Biological Agency, 194291 Saint Petersburg, Russia
* Correspondence: doctorpetrovandrey@gmail.com; Tel.: +7-9213333100

Statistical analysis

Statistical analysis was performed using StatTech v. 3.1.10 (Developer - StatTech LLC, Russia).

Quantitative variables were assessed for normality using the Shapiro-Wilk test (when the number of subjects was less than 50) or the Kolmogorov-Smirnov test (when the number of subjects was more than 50).

Quantitative variables following a normal distribution were described using mean (M) and standard deviation (SD), 95% confidence interval (95% CI) for the mean were estimated.

Quantitative variables following non normal distribution were described using median (Me) and lower and upper quartiles ($Q_1 – Q_3$).

Categorical data were described with absolute and relative frequencies.

Comparison of the two groups for a quantitative variable following a normal distribution, under the condition of equality of variances, was performed using Student's t-test.

Comparisons of three or more groups on a quantitative variable following a normal distribution were performed using one-way analysis of variance and Tukey test as a post-hoc method (assuming equal variances).

Mann-Whitney U-test was used to compare two groups on a quantitative variable whose distribution differed from the normal distribution.

Comparisons of three or more groups on a quantitative variable whose distribution differed from normal were made using the Kruskal-Wallis test and Dunn's criterion with Holm correction as a post-hoc method.

Comparison of frequencies in the analysis of 2 by 2 contingency tables was performed using Fisher's exact test (for expected values less than 10)

Comparison of frequencies in the analysis of multifield contingency tables was performed using Pearson's chi-square test (for expected values greater than 10).

The direction and strength of the association between two quantitative variables were estimated using Spearman's correlation coefficient (if at least one variable does not follow a normal distribution)

The prognostic model characterizing the dependence of a quantitative variable on predictors was developed using ordinary least squares linear regression.

The development of a prognostic model for the probability of a binary outcome was carried out using logistic regression. Nagelkerke R^2 was used as a measure of the model performance.

ROC analysis was used to assess the diagnostic performance of quantitative variables in predicting a categorical outcome. The optimal cut-off value of the quantitative variable at was estimated using the Youden's J statistic.

Table S1. Descriptive statistics for quantitative variables.

Variables	M ± SD / Me	95% CI / Q_1 – Q_3	n	min	max
Age, M ± SD (year)	55 ± 13	49 – 61	23	29	76
Number of treatment stages, Me	2	1 – 2	23	1	5
Number of NAEM embolisation steps, Me	1	1 – 1	23	1	4
mRS before embolisation, Me	0	0 – 1	23	0	3
Time of embolisation, M ± SD (min)	97 ± 28	85 – 109	23	60	170
NAEM volume, Me (ml)	7.50	5.50 – 8.00	23	4.00	16.50
mRS at discharge, Me	1	0 – 1	23	0	2

Table S2. Descriptive statistics for categorical variables.

Variables	Categories	Abs.	%	95% CI
Sex	female	16	69.6	47.1 – 86.8
	male	7	30.4	13.2 – 52.9
Type of Lesion	Arteriovenous Malformation of Face	5	21.7	7.5 – 43.7
	Carotid body paraganglioma	7	30.4	13.2 – 52.9
	Jugular paraganglioma	11	47.8	26.8 – 69.4
Open Surgical Interventions	after embolisation	4	17.4	5.0 – 38.8
	before embolisation	1	4.3	0.1 – 21.9
	none	18	78.3	56.3 – 92.5
Embolisation by other agents	adhesive	4	17.4	5.0 – 38.8
	alcohol	1	4.3	0.1 – 21.9
	coils	1	4.3	0.1 – 21.9
	none	17	73.9	51.6 – 89.8
Type of NAEM	Squid	12	52.2	30.6 – 73.2
	Onyx+Squid	2	8.7	1.1 – 28.0
	Onyx	8	34.8	16.4 – 57.3
	Phill	1	4.3	0.1 – 21.9
Coils for NAEM	none	21	91.3	72.0 – 98.9
	+coils	2	8.7	1.1 – 28.0
	total	17	73.9	51.6 – 89.8

Radicality embolisation	subtotal	6	26.1	10.2 – 48.4
Type of catheter	Scepter C, XC	21	91.3	72.0 – 98.9
	Headway	2	8.7	1.1 – 28.0
Complications	none	22	95.7	78.1 – 99.9
	cerebral ischaemia	1	4.3	0.1 – 21.9

Analysis of Age was performed conditioning on Type of Lesion.

Table S3. Analysis of Age conditioning on Type of Lesion.

Variable	Categories	Age (year)			*p*
		M ± SD	95% CI	n	
Type of Lesion	Arteriovenous Malformation of Face	42 ± 6	34 – 49	5	0.033*<br>$p_{\text{Arteriovenous Malformation of Face – Carotid body paraganglioma}}$ = 0.044<br>$p_{\text{Arteriovenous Malformation of Face – Jugular paraganglioma}}$ = 0.048
	Carotid body paraganglioma	59 ± 11	49 – 70	7	
	Jugular paraganglioma	58 ± 14	49 – 67	11	

* – differences are statistically significant ($p < 0.05$).

Statistically significant differences were revealed when comparing of Age depending on Type of Lesion ($p = 0.033$) *(applied method: One-way ANOVA)*.

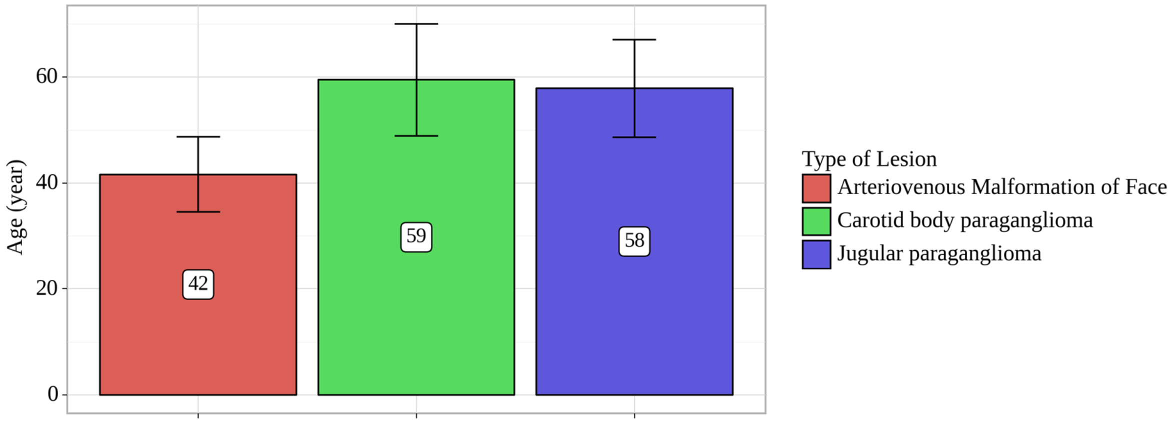

Figure S1. Analysis of Age conditioning on Type of Lesion.

We performed analysis of Number of treatment stages conditioning on Type of Lesion.

Table S4. Analysis of Number of treatment stages conditioning on Type of Lesion.

Variable	Categories	Number of treatment stages			*p*
		Me	$Q_1 – Q_3$	n	
Type of Lesion	Arteriovenous Malformation of Face	2	2 – 2	5	0.044*
	Carotid body paragangliom a	1	1 – 1	7	
	Jugular paragangliom a	2	1 – 4	11	

* – differences are statistically significant ($p < 0.05$).

In accordance with the presented table, when comparing of Number of treatment stages, statistically significant differences were revealed depending on Type of Lesion ($p = 0.044$) *(applied method: The Kruskal-Wallis test)*.

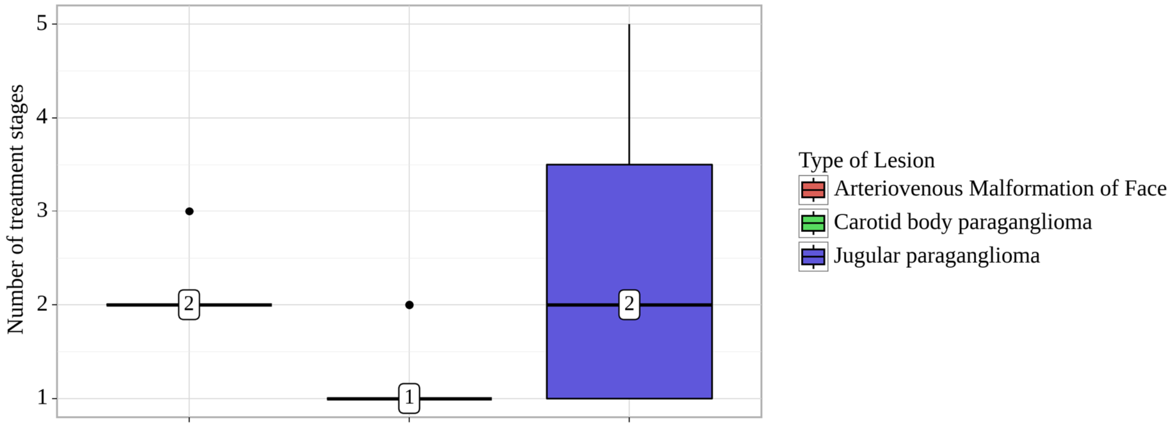

Figure S2. Analysis of Number of treatment stages conditioning on Type of Lesion.

Analysis of Number of NAEM embolisation steps was performed conditioning on Type of Lesion.

Table S5. Analysis of Number of NAEM embolisation steps conditioning on Type of Lesion.

Variable	Categories	Number of NAEM embolisation steps			*p*
		Me	$Q_1 – Q_3$	n	
Type of Lesion	Arteriovenous Malformation of Face	1	1 – 1	5	0.193
	Carotid body paragangliom a	1	1 – 1	7	
	Jugular paragangliom a	1	1 – 3	11	

When comparing of Number of NAEM embolisation steps depending on Type of Lesion no statistically significant differences were revealed ($p = 0.193$) *(applied method: The Kruskal-Wallis test)*.

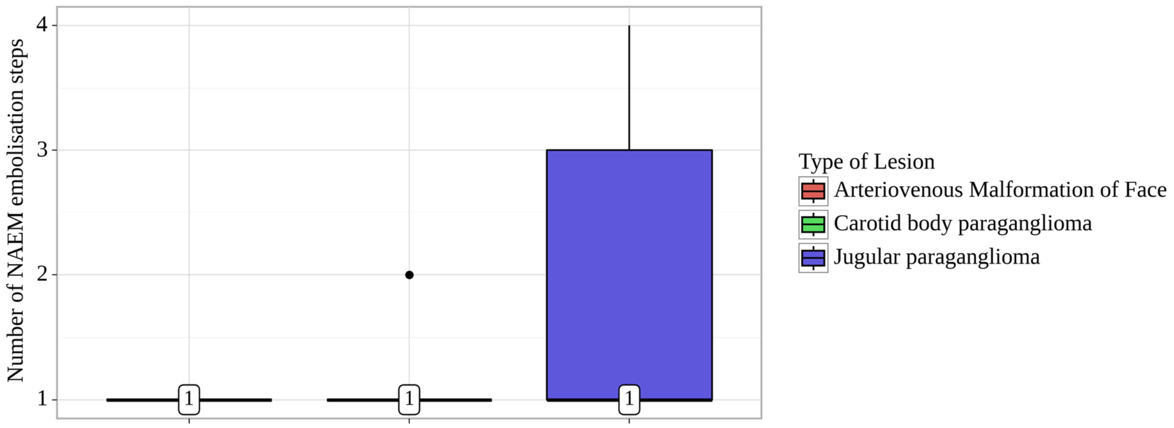

Figure S3. Analysis of Number of NAEM embolisation steps conditioning on Type of Lesion.

We performed analysis of Age conditioning on Open Surgical Interventions.

Table S6. Analysis of Age conditioning on Open Surgical Interventions.

Variable	Categories	Age (year)			*p*
		Me	$Q_1 - Q_3$	n	
Open Surgical Interventions	after embolisation	44	40 – 47	4	0.059
	before embolisation	72	72 – 72	1	
	none	57	48 – 68	18	

When comparing of Age depending on Open Surgical Interventions no statistically significant differences were revealed ($p = 0.059$) *(applied method: The Kruskal-Wallis test)*.

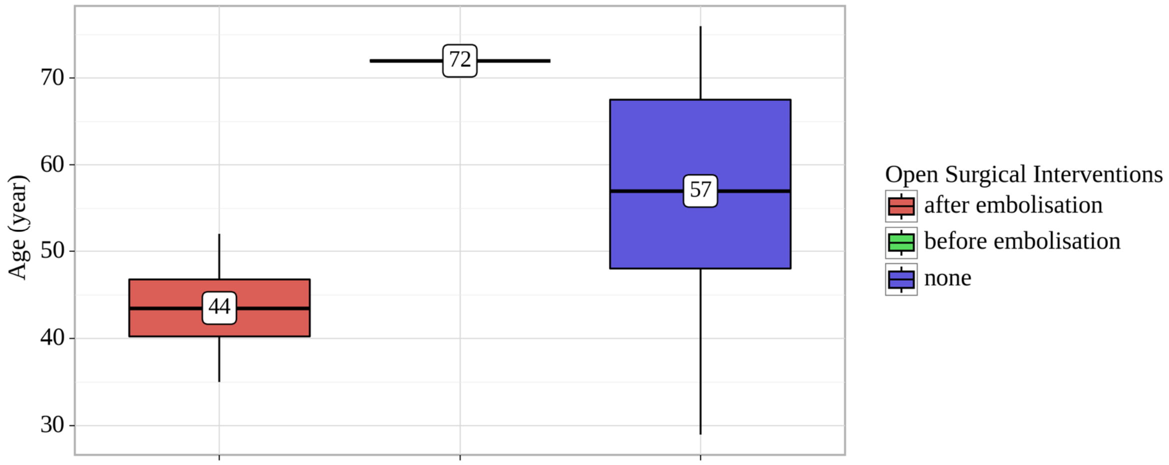

Figure S4. Analysis of Age conditioning on Open Surgical Interventions.

We performed analysis of Type of Lesion conditioning on Open Surgical Interventions.

Table S7. Analysis of Type of Lesion conditioning on Open Surgical Interventions.

Variable	Categories	Open Surgical Interventions			*p*
		after embolisation	before embolisation	none	

	Arteriovenous Malformation of Face	3 (75.0)	0 (0.0)	2 (11.1)	
Type of Lesion	Carotid body paragangliom a	0 (0.0)	0 (0.0)	7 (38.9)	0.053
	Jugular paragangliom a	1 (25.0)	1 (100.0)	9 (50.0)	

When comparing of Type of Lesion depending on Open Surgical Interventions there were no statistically significant differences (p = 0.053) *(applied method: Pearson's chi-square test)*.

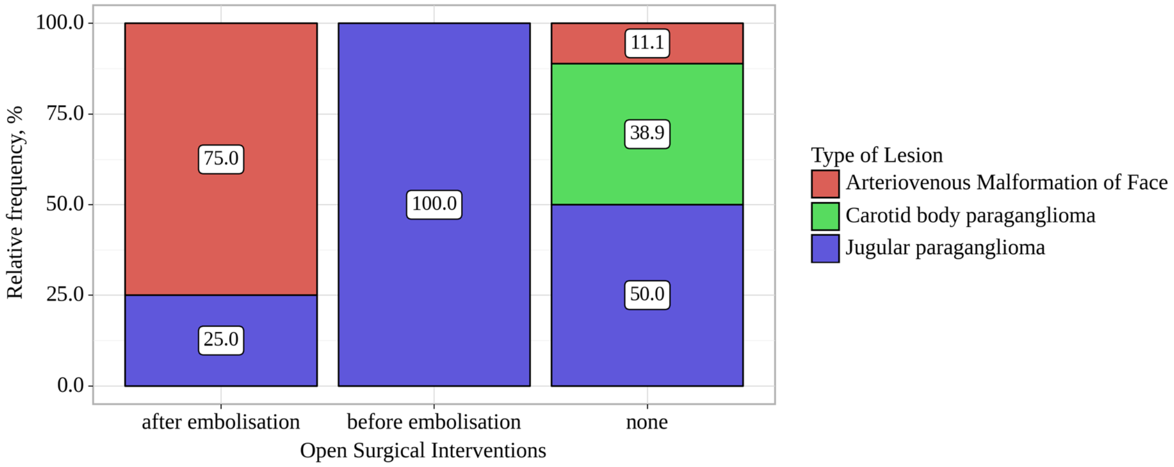

Figure S5. Analysis of Type of Lesion conditioning on Open Surgical Interventions.

We performed analysis of Radicality embolisation conditioning on Open Surgical Interventions.

Table S8. Analysis of Radicality embolisation conditioning on Open Surgical Interventions.

Variable	Categories	Open Surgical Interventions			p
		after embolisation	before embolisation	none	
Radicality embolisation	total	3 (75.0)	1 (100.0)	13 (72.2)	0.826
	subtotal	1 (25.0)	0 (0.0)	5 (27.8)	

When comparing of Radicality embolisation depending on Open Surgical Interventions no statistically significant differences were revealed (p = 0.826) *(applied method: Pearson's chi-square test)*.

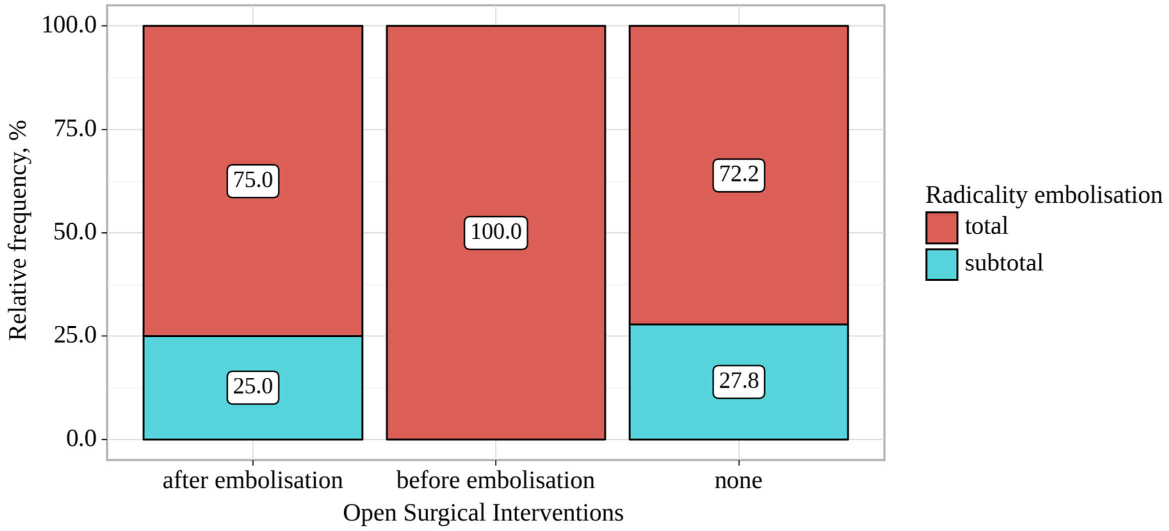

Figure S6. Analysis of Radicality embolisation conditioning on Open Surgical Interventions.

Analysis of Type of Lesion was performed conditioning on Embolisation by other agents.

Table S9. Analysis of Type of Lesion conditioning on Embolisation by other agents.

Variable	Categories	Embolisation by other agents				*p*
		adhesive	alcohol	coils	none	
Type of Lesion	Arteriovenous Malformation of Face	3 (75.0)	1 (100.0)	1 (100.0)	0 (0.0)	0.004* $p_{adhesive - none}$ = 0.002 $p_{alcohol - none}$ < 0.001 $p_{coils - none}$ < 0.001
	Carotid body paraganglioma	0 (0.0)	0 (0.0)	0 (0.0)	7 (41.2)	
	Jugular paraganglioma	1 (25.0)	0 (0.0)	0 (0.0)	10 (58.8)	

* – differences are statistically significant ($p < 0.05$).

According to the data obtained when comparing of Type of Lesion statistically significant differences were revealed depending on Embolisation by other agents (p = 0.004) *(applied method: Pearson's chi-square test)*.

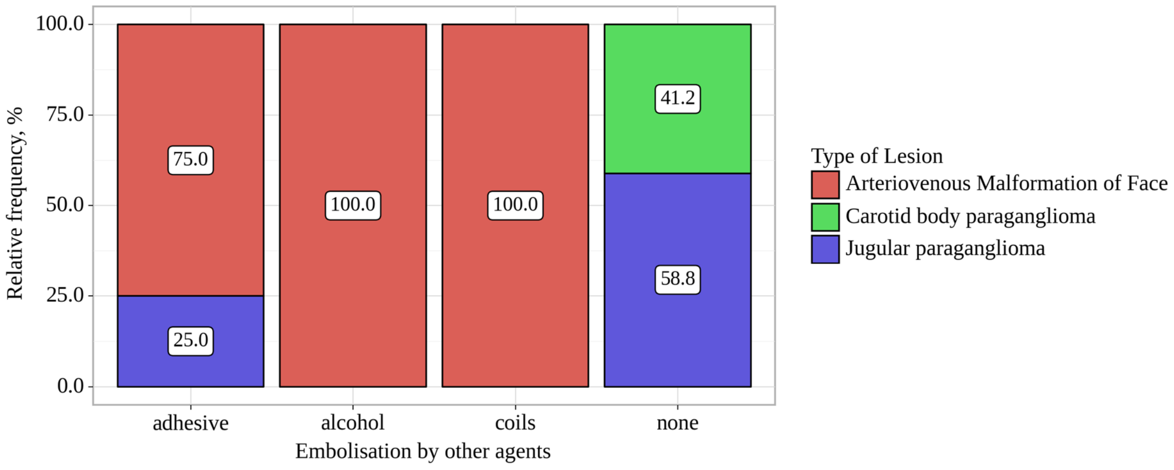

Figure S7. Analysis of Type of Lesion conditioning on Embolisation by other agents.

We performed analysis of Number of treatment stages conditioning on Embolisation by other agents.

Table S10. Analysis of Number of treatment stages conditioning on Embolisation by other agents.

Variable	Categories	Number of treatment stages			p
		Me	$Q_1 - Q_3$	n	
Embolisation by other agents	adhesive	2	2 – 2	4	0.347
	alcohol	3	3 – 3	1	
	coils	2	2 – 2	1	
	none	1	1 – 2	17	

When comparing of Number of treatment stages depending on Embolisation by other agents there were no statistically significant differences (p = 0.347) *(applied method: The Kruskal-Wallis test)*.

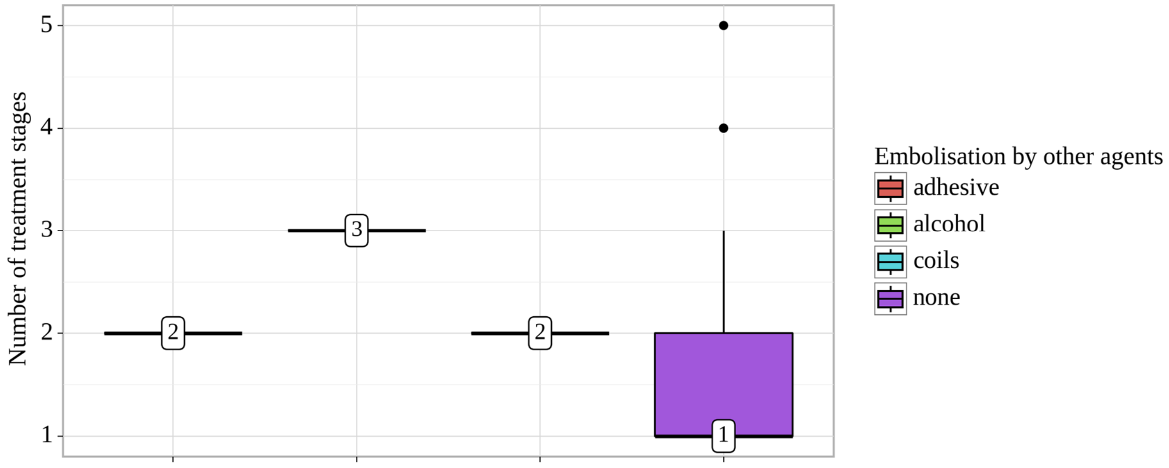

Figure S8. Analysis of Number of treatment stages conditioning on Embolisation by other agents.

Correlation analysis of the association between mRS before embolisation and Age was performed.

Table S11. Results of the correlation analysis of the association between mRS before embolisation and Age.

Variable	Correlation characteristics		
	ϱ	Strength of the association assesed using Chaddock scale	*p*
mRS before embolisation – Age	0.159	Weak	0.468

A weak correlation positive association between Age and mRS before embolisation was estimated.

Observed dependence of Age from mRS before embolisation is described by a linear regression equation:

$$Y_{Age} = -0.745 \times X_{mRS\ before\ embolisation} + 55.204 \qquad (1)$$

With an 1 decrease of mRS before embolisation 0.745 year change of Age should be expected. According to the coefficient of determination R^2 of the resulting model, 0.2% of the observed variance of Age were explained.

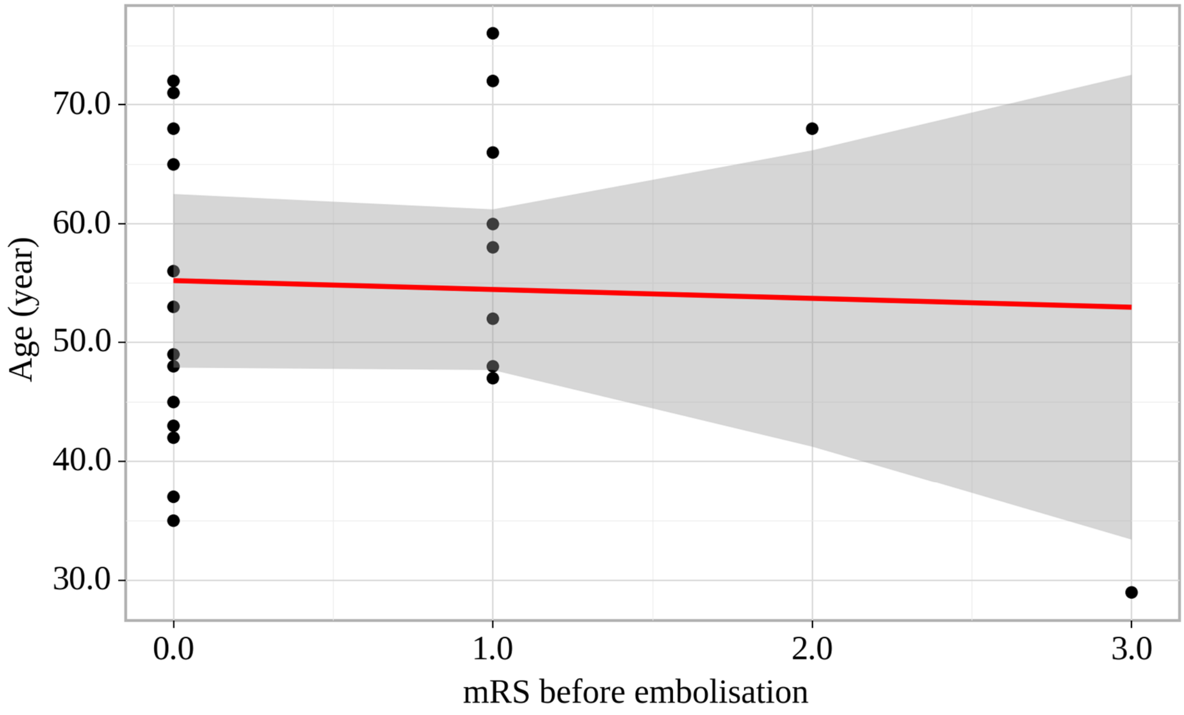

Figure S9. Regression line characterizing the dependence of Age from mRS before embolisation.

Analysis of mRS before embolisation was performed conditioning on Type of Lesion.

Table S12. Analysis of mRS before embolisation conditioning on Type of Lesion.

Variable	Categories	mRS before embolisation			*p*
		Me	$Q_1 - Q_3$	n	
Type of Lesion	Arteriovenous Malformation of Face	0	0 – 0	5	0.002* $p_{Jugular\ paraganglioma\ –\ Arteriovenous\ Malformation\ of\ Face}$ = 0.010
	Carotid body paragangliom a	0	0 – 0	7	

Jugular paraganglioma	1	1 – 1	11	$p_{\text{Jugular paraganglioma – Carotid body paraganglioma}}$ = 0.011

* – differences are statistically significant ($p < 0.05$).

According to the presented table, when comparing of mRS before embolisation, statistically significant differences were revealed depending on Type of Lesion (p = 0.002) *(applied method: The Kruskal-Wallis test)*.

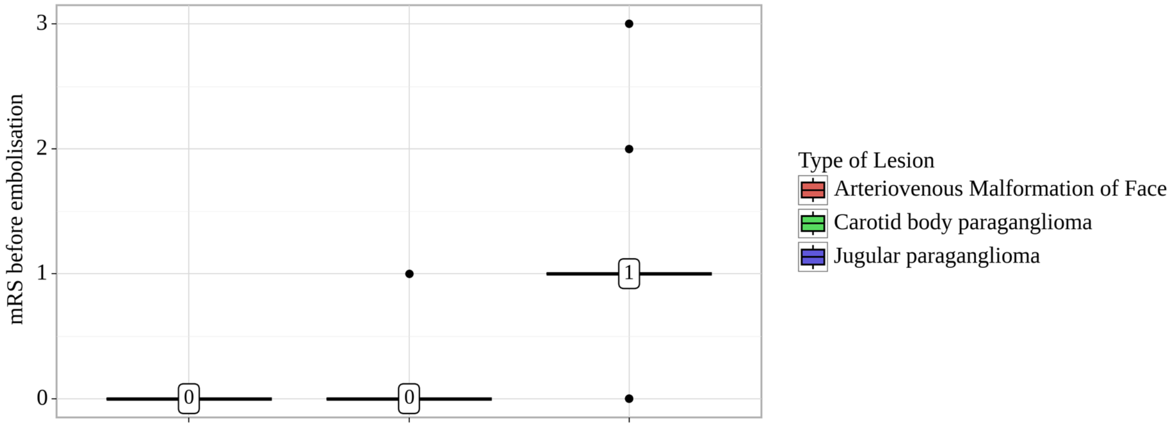

Figure S10. Analysis of mRS before embolisation conditioning on Type of Lesion.

We performed a correlation analysis of the association between mRS before embolisation and Number of treatment stages.

Table S13. Results of the correlation analysis of the association between mRS before embolisation and Number of treatment stages.

Variable	Correlation characteristics		
	ρ	Strength of the association assesed using Chaddock scale	p
mRS before embolisation – Number of treatment stages	0.277	Weak	0.200

A weak correlation positive association between Number of treatment stages and mRS before embolisation was estimated.

Observed dependence of Number of treatment stages from mRS before embolisation is described by a linear regression equation:

$$Y_{Number\ of\ treatment\ stages} = 0.596\ \times\ X_{mRS\ before\ embolisation}\ + 1.576 \qquad (2)$$

With an 1 increase of mRS before embolisation 0.596 change of Number of treatment stages should be expected. According to the coefficient of determination R^2 of the resulting model, 16.2% of the observed variance of Number of treatment stages were explained..

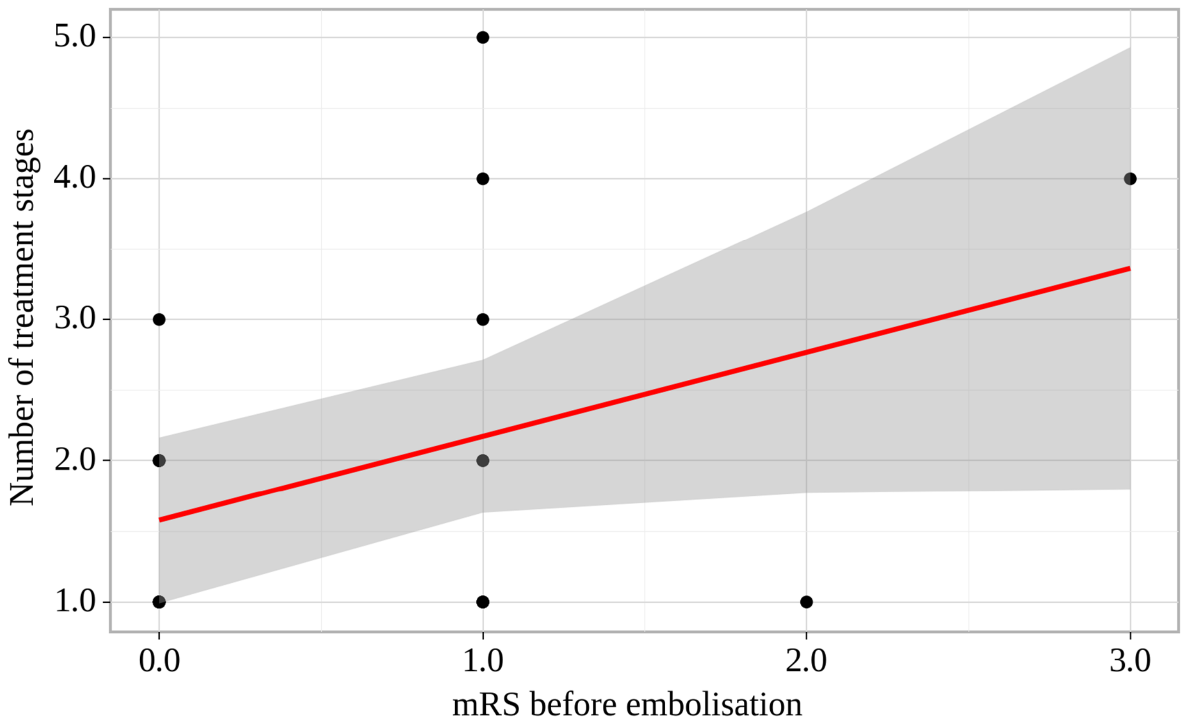

Figure S11. Regression line characterizing the dependence of Number of treatment stages from mRS before embolisation.

Correlation analysis of the association between mRS before embolisation and Number of NAEM embolisation steps was performed.

Table S14. Results of the correlation analysis of the association between mRS before embolisation and Number of NAEM embolisation steps.

Variable	Correlation characteristics		
	ρ	Strength of the association assesed using Chaddock scale	*p*
mRS before embolisation – Number of NAEM embolisation steps	0.435	Moderate	0.038*

* – differences are statistically significant ($p < 0.05$).

A moderate correlation positive association between Number of NAEM embolisation steps and mRS before embolisation was estimated.

Observed dependence of Number of NAEM embolisation steps from mRS before embolisation is described by a linear regression equation:

$$Y_{Number\ of\ NAEM\ embolisation\ steps} = 0.538 \times X_{mRS\ before\ embolisation} + 1.131 \quad (3)$$

With an 1 increase of mRS before embolisation 0.538 change of Number of NAEM embolisation steps should be expected. According to the coefficient of determination R^2 of the resulting model, 22.4% of the observed variance of Number of NAEM embolisation steps were explained.

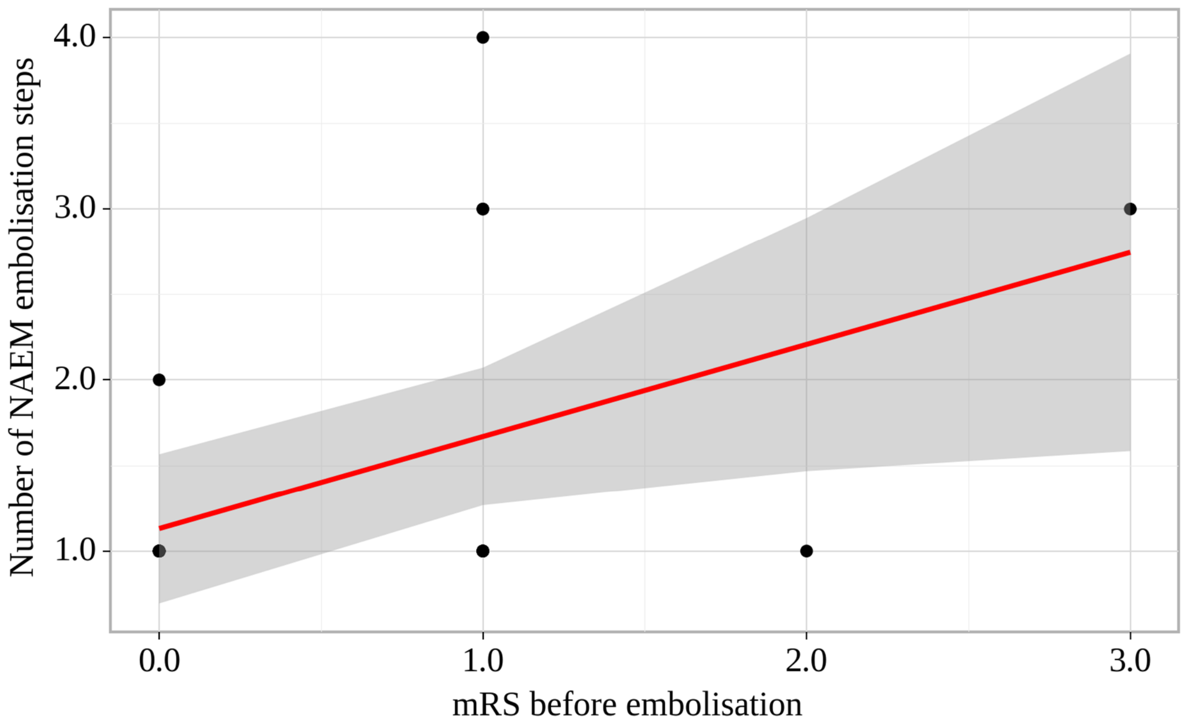

Figure S12. Regression line characterizing the dependence of Number of NAEM embolisation steps from mRS before embolisation.

Analysis of mRS before embolisation was performed conditioning on Embolisation by other agents.

Table S15. Analysis of mRS before embolisation conditioning on Embolisation by other agents.

Variable	Categories	mRS before embolisation			p
		Me	$Q_1 - Q_3$	n	
Embolisation by other agents	adhesive	0	0 – 0	4	0.449
	alcohol	0	0 – 0	1	
	coils	0	0 – 0	1	
	none	1	0 – 1	17	

When comparing of mRS before embolisation depending on Embolisation by other agents there were no statistically significant differences (p = 0.449) *(applied method: The Kruskal-Wallis test)*.

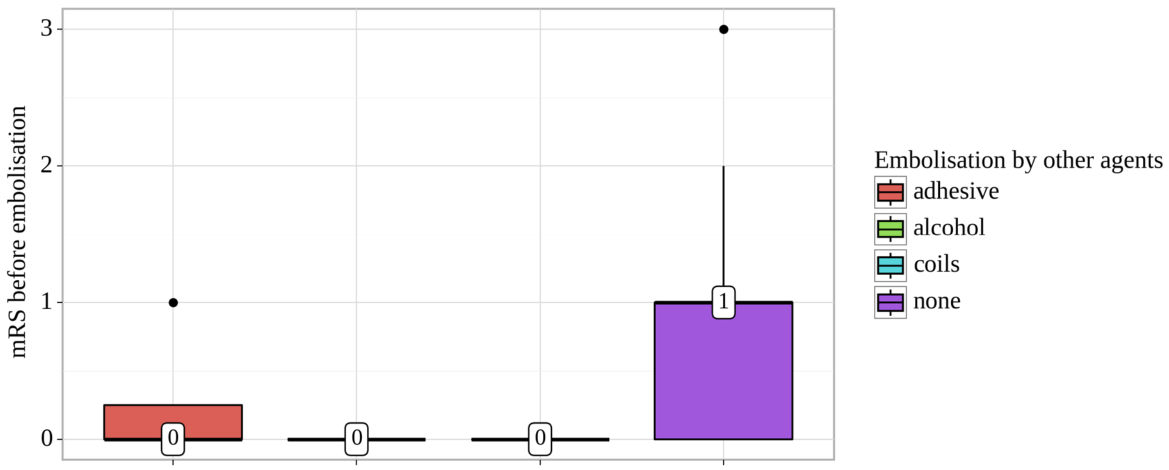

Figure S13. Analysis of mRS before embolisation conditioning on Embolisation by other agents.

We performed analysis of mRS before embolisation conditioning on Complications.

Table S16. Analysis of mRS before embolisation conditioning on Complications.

Variable	Categories	mRS before embolisation			*p*
		Me	$Q_1 – Q_3$	n	
Complications	none	0	0 – 1	22	0.088
	cerebral ischaemia	2	2 – 2	1	

When comparing of mRS before embolisation depending on Complications there were no statistically significant differences (p = 0.088) *(applied method: Mann-Whitney U-test)*.

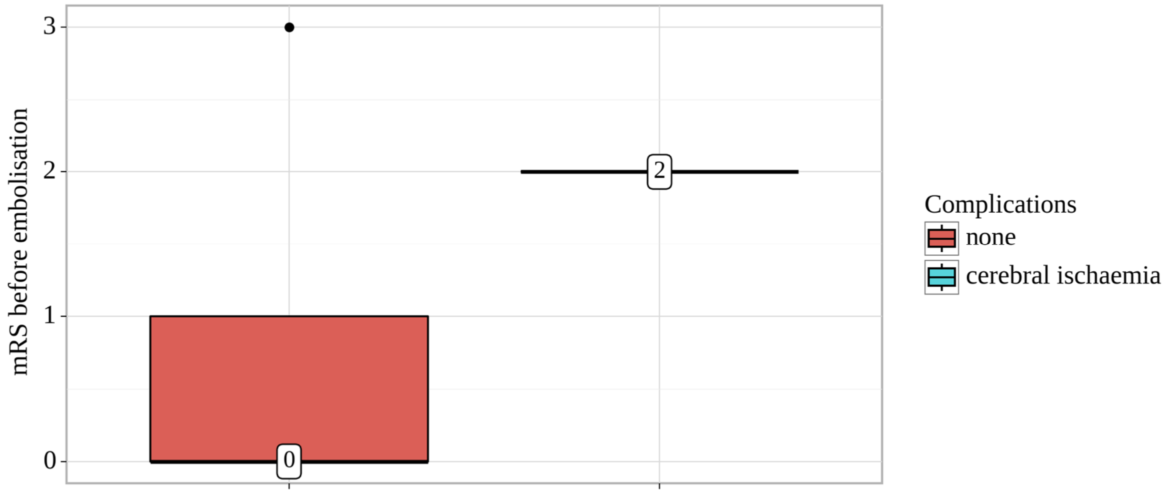

Figure S14. Analysis of mRS before embolisation conditioning on Complications.

When evaluating the dependence of the probability of cerebral ischaemia on the mRS before embolisation using the ROC analysis, the following curve was obtained.

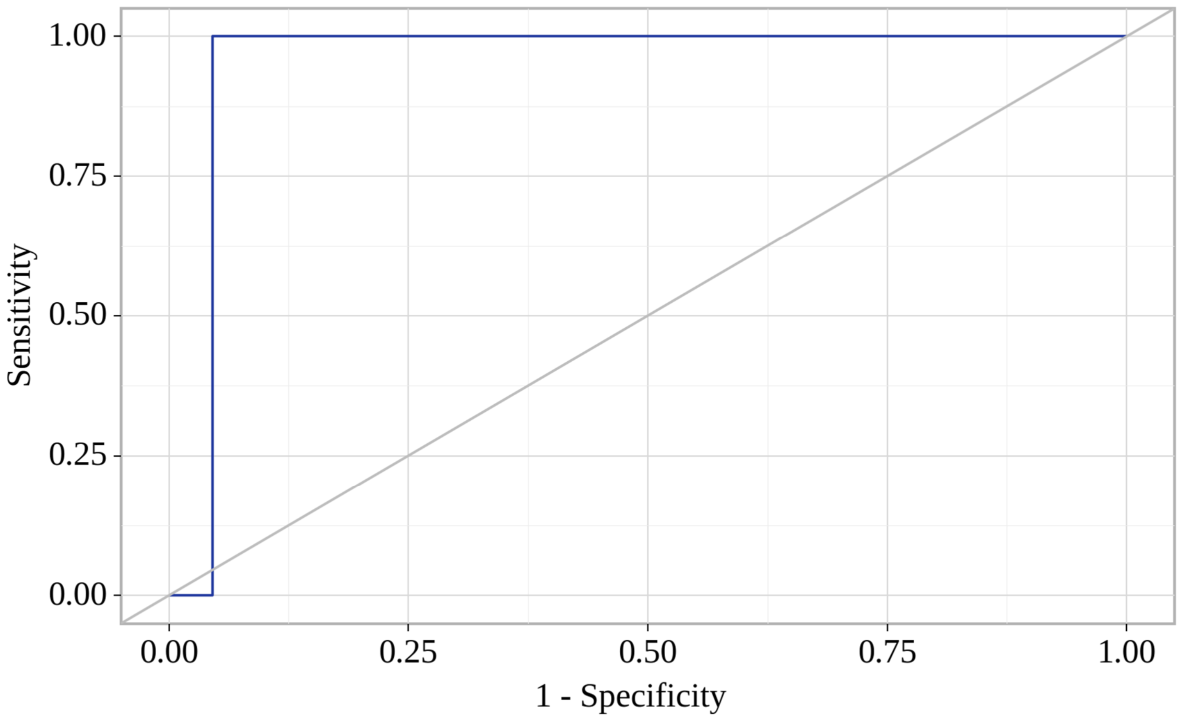

Figure S15. ROC-curve characterizing the dependence of the probability Complications on mRS before embolisation.

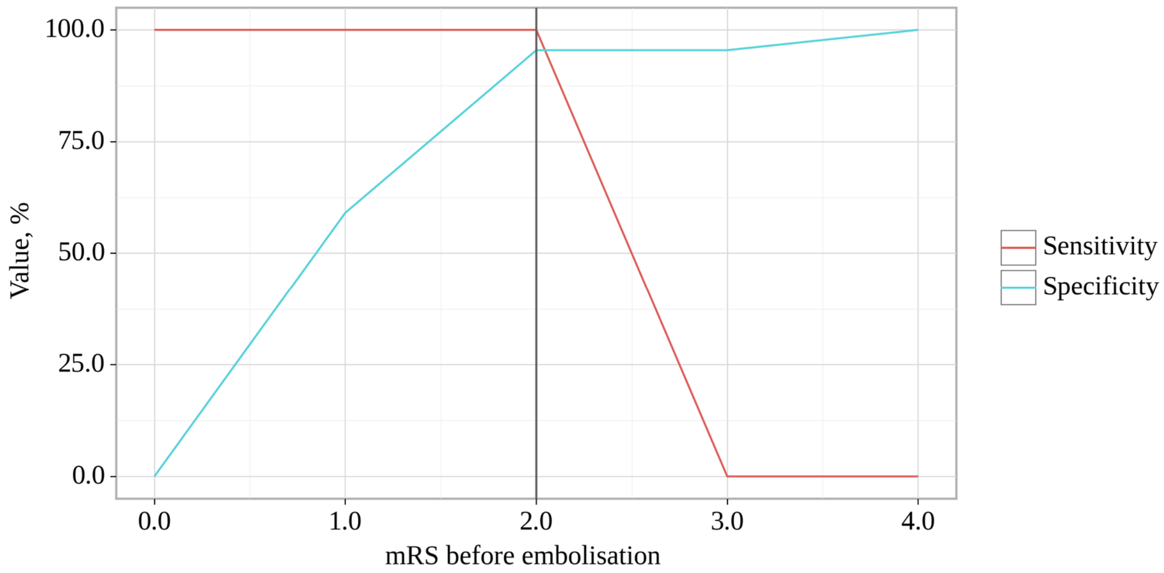

Figure S16. Analysis of the sensitivity and specificity of Complications depending on mRS before embolisation.

Table S17. Threshold mRS before embolisation.

Threshold	Sensitivity (Se), %	Specificity (Sp), %	PPV	NPV
**2**	**100.0**	**95.5**	**50.0**	**100.0**
1	100.0	59.1	10.0	100.0

The area under the ROC curve comprised 0.955 ± 0.149 with 95% CI: 0.663 - 1.000. The resulting model was not statistically significant (p = 0.088).

The cut-off value of mRS before embolisation which corresponds to the highest Youden's J statistic is 2.000. If mRS before embolisation was greater than or equal to this

value, cerebral ischaemia was predicted. The sensitivity and specificity of the method were 100.0% and 95.5%, respectively.

We performed analysis of Time of embolisation conditioning on Type of Lesion.

Table S18. Analysis of Time of embolisation conditioning on Type of Lesion.

Variable	Categories	Time of embolisation (min)			p
		Me	$Q_1 – Q_3$	n	
Type of Lesion	Arteriovenous Malformation of Face	70	70 – 75	5	0.035* $p_{Carotid\ body\ paraganglioma – Arteriovenous\ Malformation\ of\ Face}$ = 0.048 $p_{Jugular\ paraganglioma – Arteriovenous\ Malformation\ of\ Face}$ = 0.049
	Carotid body paraganglioma	100	90 – 115	7	
	Jugular paraganglioma	100	82 – 120	11	

* – differences are statistically significant ($p < 0.05$).

According to the data obtained when comparing of Time of embolisation statistically significant differences were revealed depending on Type of Lesion (p = 0.035) *(applied method: The Kruskal-Wallis test)*.

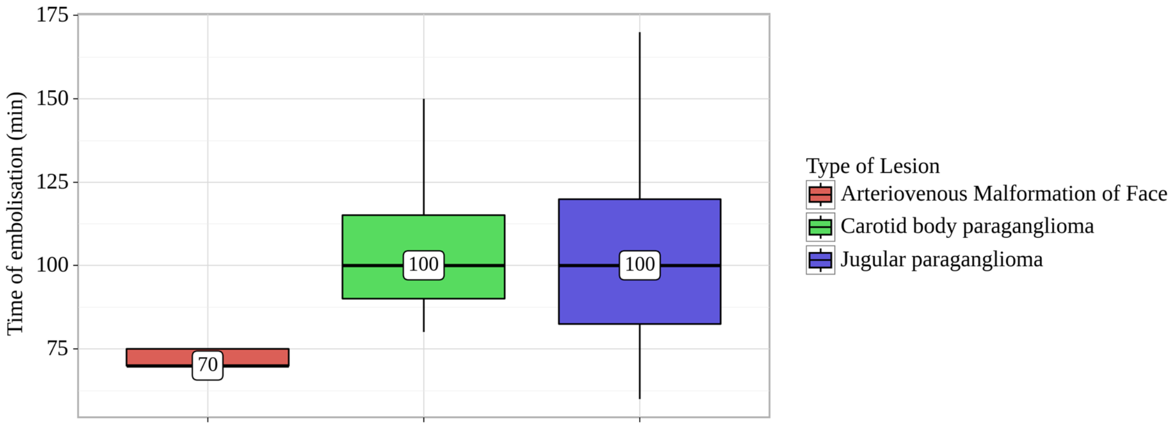

Figure S17. Analysis of Time of embolisation conditioning on Type of Lesion.

We performed analysis of Time of embolisation conditioning on Embolisation by other agents.

Table S19. Analysis of Time of embolisation conditioning on Embolisation by other agents.

Variable	Categories	Time of embolisation (min)			p
		Me	$Q_1 – Q_3$	n	
Embolisation by other agents	adhesive	70	68 – 71	4	0.019* $p_{none – adhesive}$ = 0.030
	alcohol	70	70 – 70	1	
	coils	75	75 – 75	1	
	none	100	90 – 120	17	

* – differences are statistically significant ($p < 0.05$).

In accordance with the presented table, when comparing of Time of embolisation, statistically significant differences were revealed depending on Embolisation by other agents (p = 0.019) *(applied method: The Kruskal-Wallis test)*.

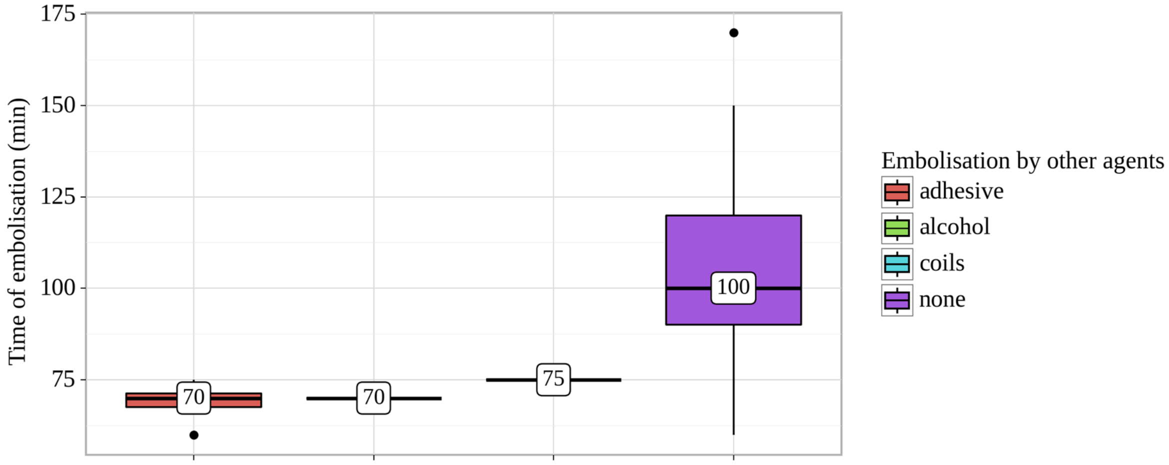

Figure S18. Analysis of Time of embolisation conditioning on Embolisation by other agents.

We performed analysis of Time of embolisation conditioning on Type of NAEM.

Table S20. Analysis of Time of embolisation conditioning on Type of NAEM.

Variable	Categories	Time of embolisation (min)			*p*
		Me	$Q_1 - Q_3$	n	
Type of NAEM	Squid	95	75 – 112	12	0.280
	Onyx+Squid	110	105 – 115	2	
	Onyx	78	70 – 95	8	
	Phill	120	120 – 120	1	

When comparing of Time of embolisation depending on Type of NAEM no statistically significant differences were revealed (p = 0.280) (*applied method: The Kruskal-Wallis test*).

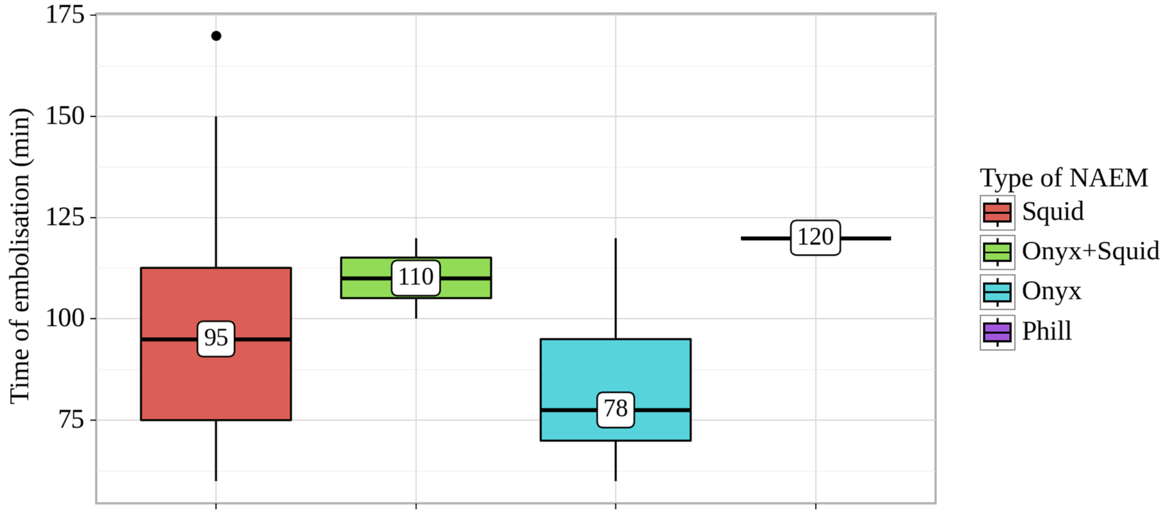

Figure S19. Analysis of Time of embolisation conditioning on Type of NAEM.

We performed analysis of Time of embolisation conditioning on Coils for NAEM.

Table S21. Analysis of Time of embolisation conditioning on Coils for NAEM.

Variable	Categories	Time of embolisation (min)			*p*
		Me	$Q_1 - Q_3$	n	
	none	90	75 – 120	21	0.350

Coils for NAEM	+coils	80	70 – 90	2

When comparing of Time of embolisation depending on Coils for NAEM no statistically significant differences were revealed (p = 0.350) *(applied method: Mann-Whitney U-test)*.

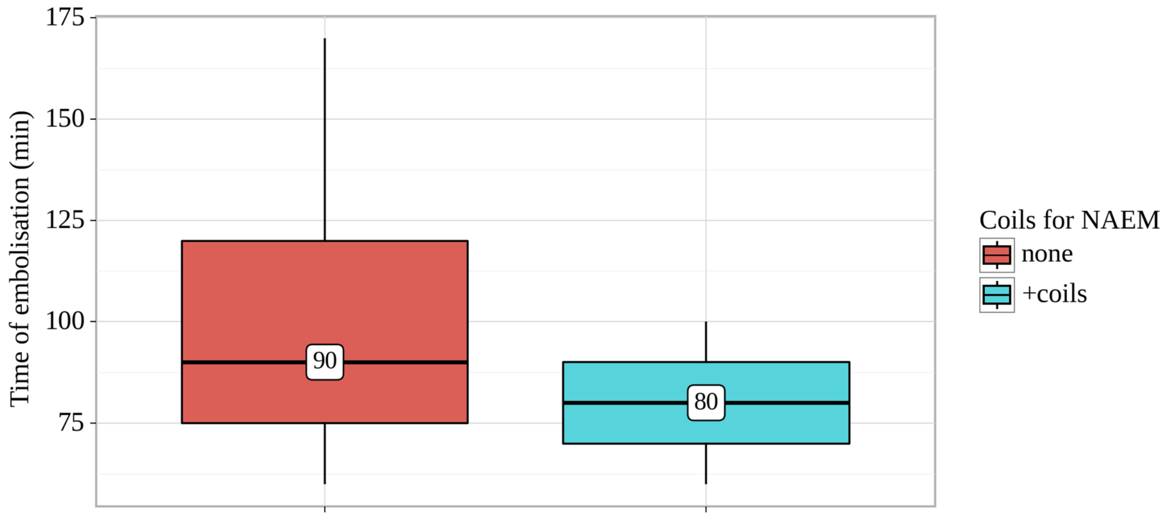

Figure S20. Analysis of Time of embolisation conditioning on Coils for NAEM.

When evaluating the dependence of the probability of +coils on the Time of embolisation using the ROC analysis, the following curve was obtained.

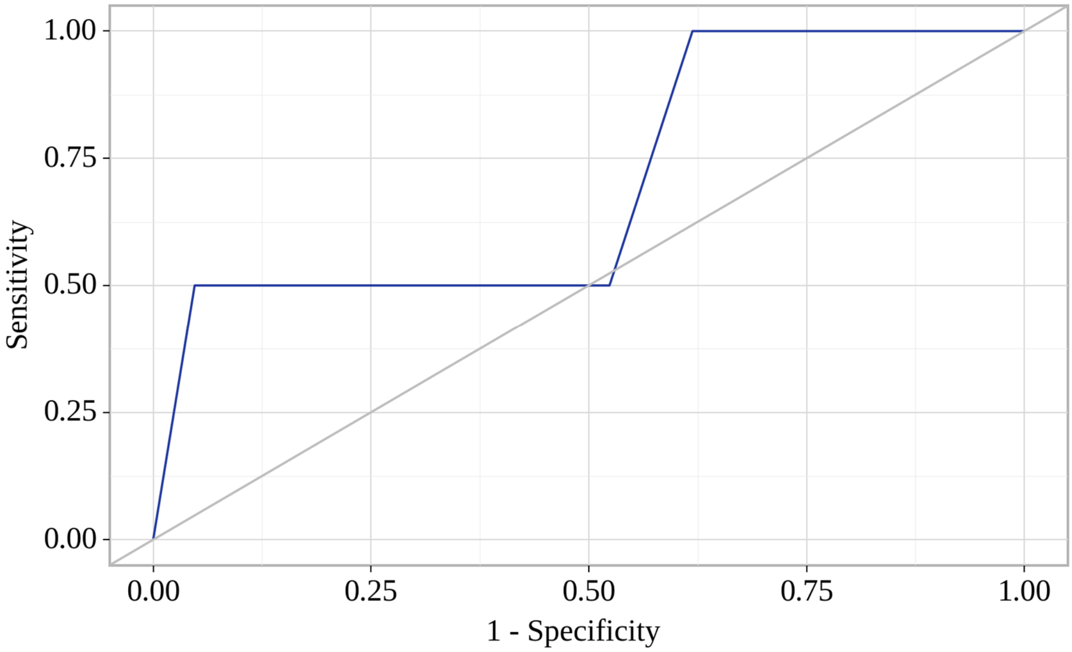

Figure S21. ROC-curve characterizing the dependence of the probability Coils for NAEM on Time of embolisation.

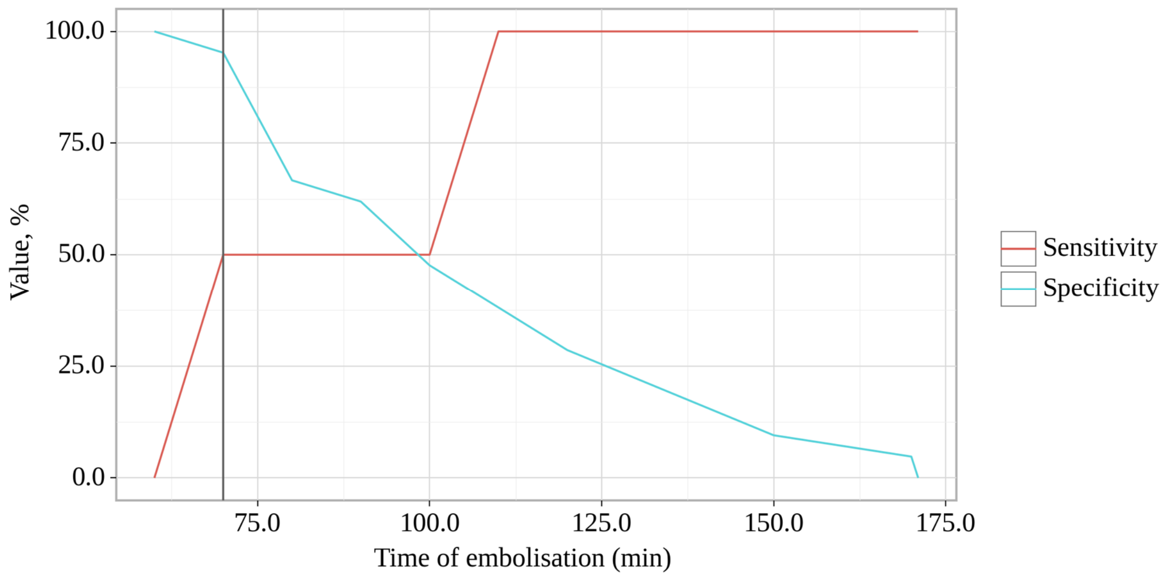

Figure S22. Analysis of the sensitivity and specificity of Coils for NAEM depending on Time of embolisation.

Table S22. Threshold Time of embolisation.

Threshold	Sensitivity (Se), %	Specificity (Sp), %	PPV	NPV
**90**	**50.0**	**61.9**	**11.1**	**92.9**
80	50.0	66.7	12.5	93.3
70	50.0	95.2	50.0	95.2

The area under the ROC curve comprised 0.702 ± 0.173 with 95% CI: 0.364 - 1.000. The resulting model was not statistically significant (p = 0.350).

The cut-off value of Time of embolisation which corresponds to the highest Youden's J statistic is 70.000 min. If Time of embolisation was less than this value, +coils was predicted. The sensitivity and specificity of the method were 50.0% and 95.2%, respectively.

We performed analysis of Time of embolisation conditioning on Radicality embolisation.

Table S23. Analysis of Time of embolisation conditioning on Radicality embolisation.

Variable	Categories	Time of embolisation (min)			p
		M ± SD	95% CI	n	
Radicality embolisation	total	98 ± 30	82 – 113	17	0.801
	subtotal	94 ± 24	69 – 120	6	

When comparing of Time of embolisation depending on Radicality embolisation there were no statistically significant differences (p = 0.801) *(applied method: Student's t-test)*.

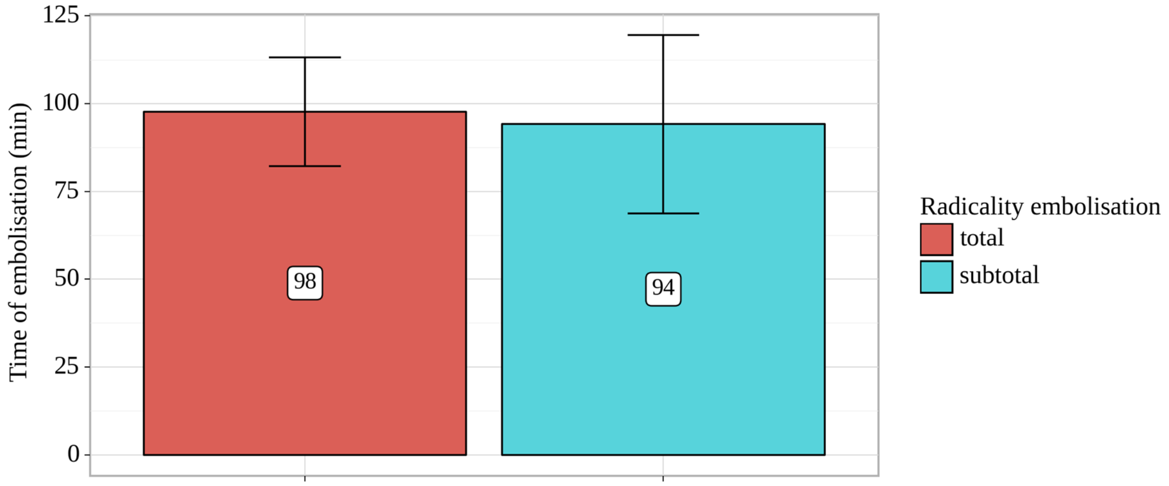

Figure S23. Analysis of Time of embolisation conditioning on Radicality embolisation.

When evaluating the dependence of the probability of subtotal on the Time of embolisation using the ROC analysis, the following curve was obtained.

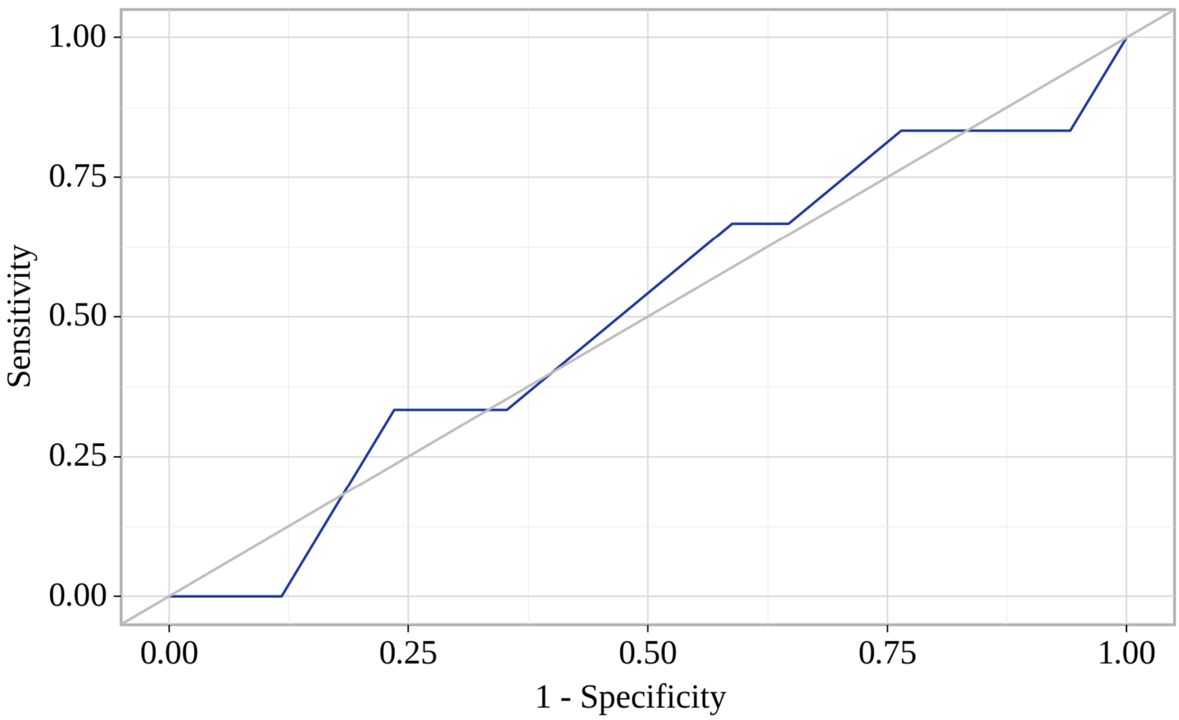

Figure S24. ROC-curve characterizing the dependence of the probability Radicality embolisation on Time of embolisation.

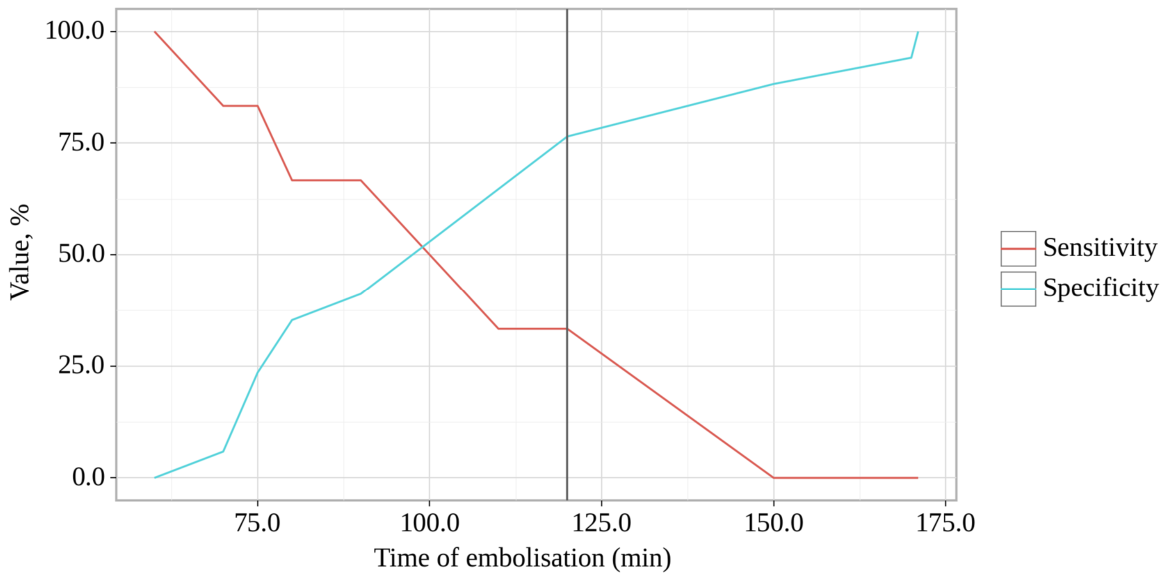

Figure S25. Analysis of the sensitivity and specificity of Radicality embolisation depending on Time of embolisation.

The area under the ROC curve comprised 0.505 ± 0.140 with 95% CI: 0.230 - 0.780. The resulting model was not statistically significant (p = 0.972).

The cut-off value of Time of embolisation which corresponds to the highest Youden's J statistic is 120.000 min. If Time of embolisation was greater than or equal to this value, subtotal was predicted. The sensitivity and specificity of the method were 33.3% and 76.5%, respectively.

We performed analysis of Time of embolisation conditioning on Type of catheter.

Table S24. Analysis of Time of embolisation conditioning on Type of catheter.

Variable	Categories	Time of embolisation (min)			p
		Me	$Q_1 – Q_3$	n	
Type of catheter	Scepter C, XC	100	75 – 120	21	0.206
	Headway	75	68 – 82	2	

When comparing of Time of embolisation depending on Type of catheter there were no statistically significant differences (p = 0.206) *(applied method: Mann-Whitney U-test)*.

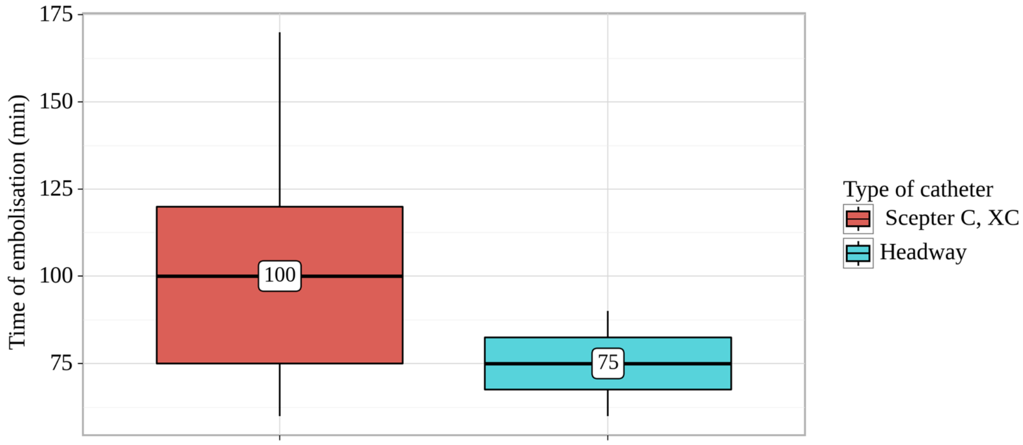

Figure S26. Analysis of Time of embolisation conditioning on Type of catheter.

When evaluating the dependence of the probability of Headway on the Time of embolisation using the ROC analysis, the following curve was obtained.

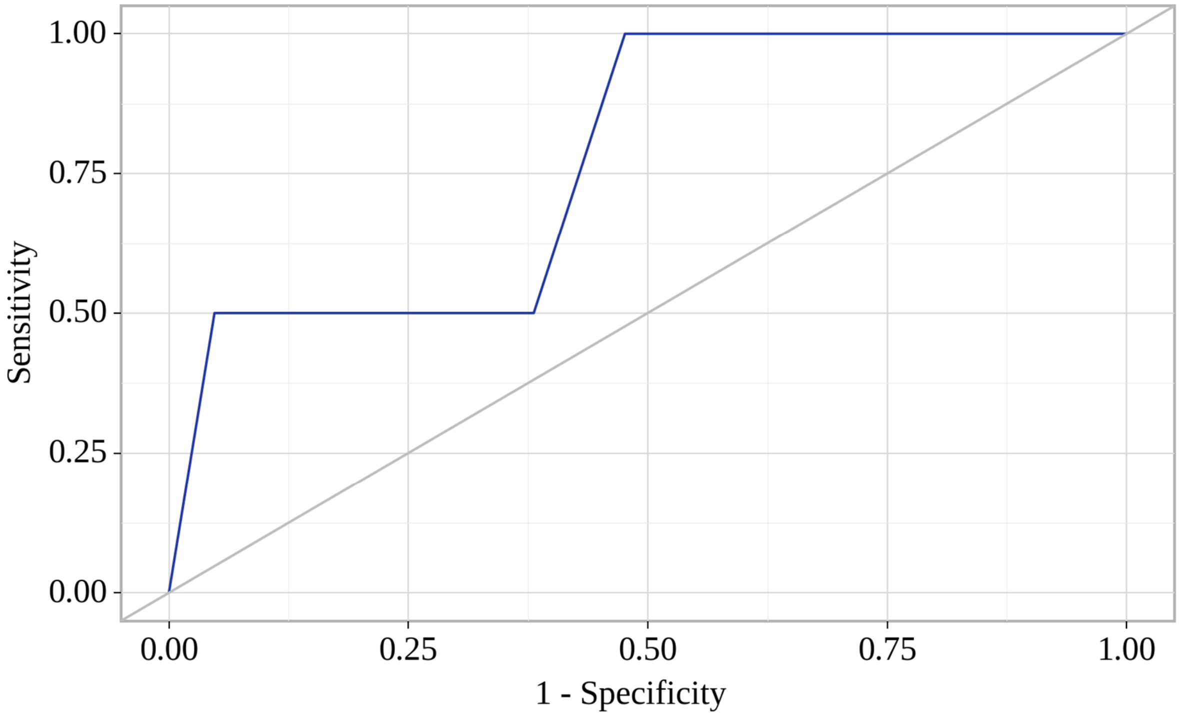

Figure S27. ROC-curve characterizing the dependence of the probability Type of catheter on Time of embolisation.

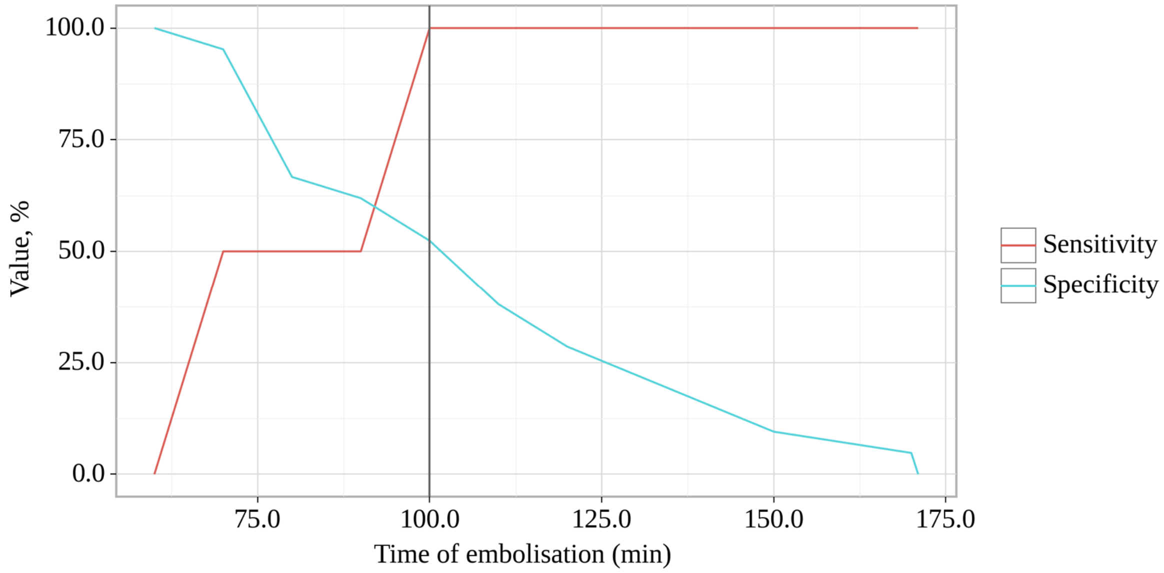

Figure S28. Analysis of the sensitivity and specificity of Type of catheter depending on Time of embolisation.

Table S25. Threshold Time of embolisation.

Threshold	Sensitivity (Se), %	Specificity (Sp), %	PPV	NPV
100	100.0	52.4	16.7	100.0
**90**	**50.0**	**61.9**	**11.1**	**92.9**
80	50.0	66.7	12.5	93.3

70	50.0	95.2	50.0	95.2

The area under the ROC curve comprised 0.774 ± 0.146 with 95% CI: 0.487 - 1.000. The resulting model was not statistically significant ($p = 0.206$).

The cut-off value of Time of embolisation which corresponds to the highest Youden's J statistic is 100.000 min. If Time of embolisation was less than this value, Headway was predicted. The sensitivity and specificity of the method were 100.0% and 52.4%, respectively.

We performed a correlation analysis of the association between Time of embolisation and NAEM volume.

Table S26. Results of the correlation analysis of the association between Time of embolisation and NAEM volume.

Variable	Correlation characteristics		
	ρ	Strength of the association assesed using Chaddock scale	*p*
Time of embolisation – NAEM volume	0.395	Moderate	0.062

A moderate correlation positive association between NAEM volume and Time of embolisation was estimated.

Observed dependence of NAEM volume from Time of embolisation is described by a linear regression equation:

$$Y_{\text{NAEM volume}} = 0.023 \times X_{\text{Time of embolisation}} + 5.109$$

With an 1 min increase of Time of embolisation 0.023 ml change of NAEM volume should be expected. According to the coefficient of determination R^2 of the resulting model, 6.0% of the observed variance of NAEM volume were explained..

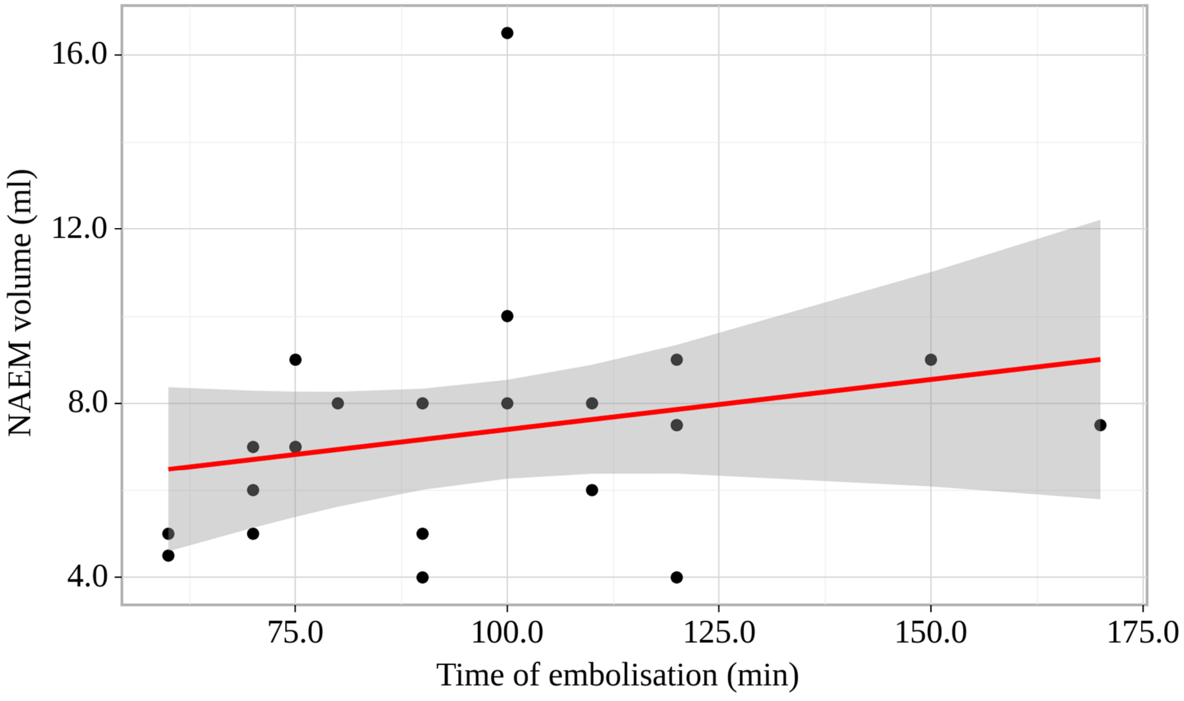

Figure S29 – Regression line characterizing the dependence of NAEM volume from Time of embolisation

Analysis of Type of Lesion was performed conditioning on Type of NAEM.

Table S27 – Analysis of Type of Lesion conditioning on Type of NAEM

Variable	Categories	Type of NAEM				*p*
		Squid	Onyx+Squid	Onyx	Phill	
Type of Lesion	Arteriovenous Malformation of Face	3 (25.0)	0 (0.0)	2 (25.0)	0 (0.0)	0.703
	Carotid body paraganglioma	4 (33.3)	0 (0.0)	3 (37.5)	0 (0.0)	
	Jugular paraganglioma	5 (41.7)	2 (100.0)	3 (37.5)	1 (100.0)	

When comparing of Type of Lesion depending on Type of NAEM no statistically significant differences were revealed (*p* = 0.703) *(applied method: Pearson's chi-square test)*.

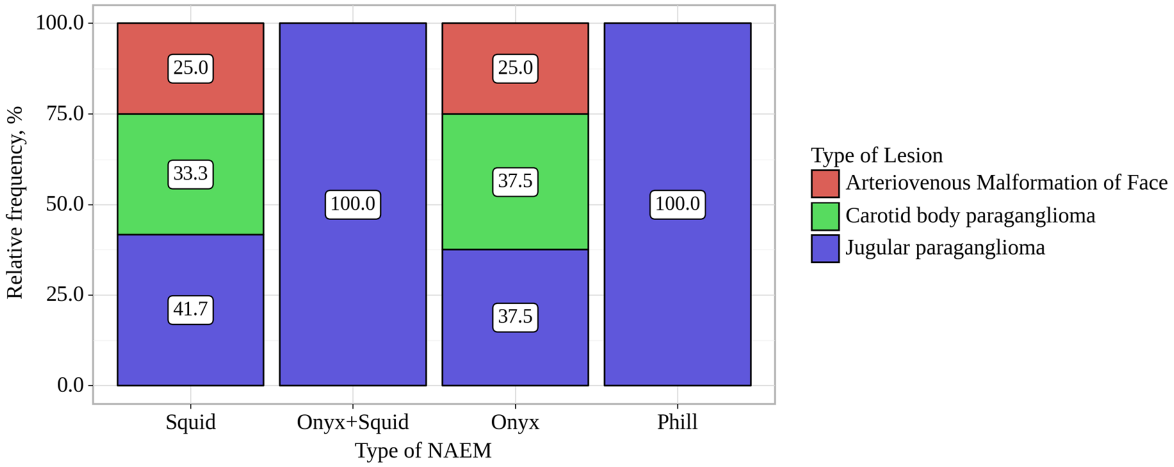

Figure S30 – Analysis of Type of Lesion conditioning on Type of NAEM

We performed analysis of Number of treatment stages conditioning on Type of NAEM.

Table S28 – Analysis of Number of treatment stages conditioning on Type of NAEM

Variable	Categories	Number of treatment stages			*p*
		Me	$Q_1 – Q_3$	n	
Type of NAEM	Squid	2	1 – 2	12	0.190
	Onyx+Squid	4	3 – 4	2	
	Onyx	2	1 – 2	8	
	Phill	1	1 – 1	1	

When comparing of Number of treatment stages depending on Type of NAEM there were no statistically significant differences (*p* = 0.190) *(applied method: The Kruskal-Wallis test)*.

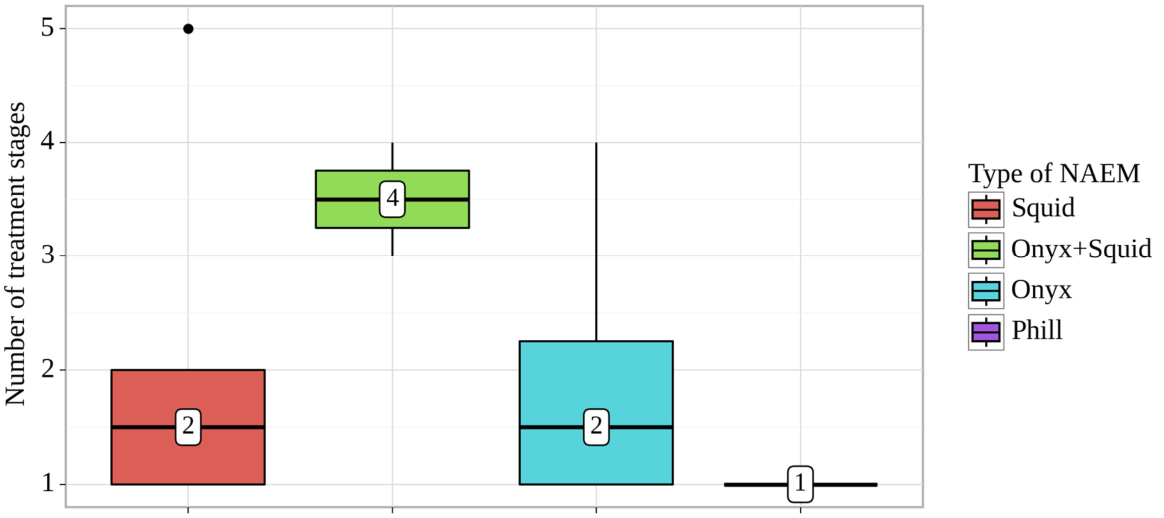

Figure S31 – Analysis of Number of treatment stages conditioning on Type of NAEM

We performed analysis of Number of NAEM embolisation steps conditioning on Type of NAEM.

Table S29 – Analysis of Number of NAEM embolisation steps conditioning on Type of NAEM

Variable	Categories	Number of NAEM embolisation steps			*p*
		Me	$Q_1 – Q_3$	n	
Type of NAEM	Squid	1	1 – 1	12	0.056
	Onyx+Squid	3	3 – 3	2	
	Onyx	1	1 – 1	8	
	Phill	1	1 – 1	1	

When comparing of Number of NAEM embolisation steps depending on Type of NAEM there were no statistically significant differences ($p = 0.056$) *(applied method: The Kruskal-Wallis test)*.

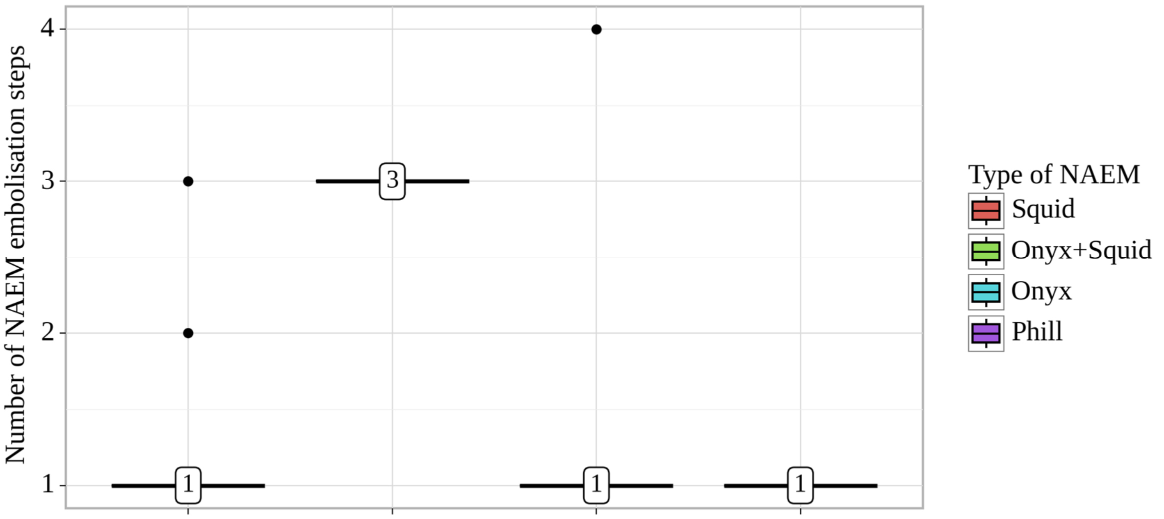

Figure S32 – Analysis of Number of NAEM embolisation steps conditioning on Type of NAEM

We performed analysis of Embolisation by other agents conditioning on Type of NAEM.

Table S30 – Analysis of Embolisation by other agents conditioning on Type of NAEM

Variable	Categories	Type of NAEM				*p*
		Squid	Onyx+Squid	Onyx	Phill	
Embolisation by other agents	adhesive	2 (16.7)	0 (0.0)	2 (25.0)	0 (0.0)	0.911
	alcohol	0 (0.0)	0 (0.0)	1 (12.5)	0 (0.0)	
	coils	1 (8.3)	0 (0.0)	0 (0.0)	0 (0.0)	
	none	9 (75.0)	2 (100.0)	5 (62.5)	1 (100.0)	

When comparing of Embolisation by other agents depending on Type of NAEM no statistically significant differences were revealed ($p = 0.911$) *(applied method: Pearson's chi-square test)*.

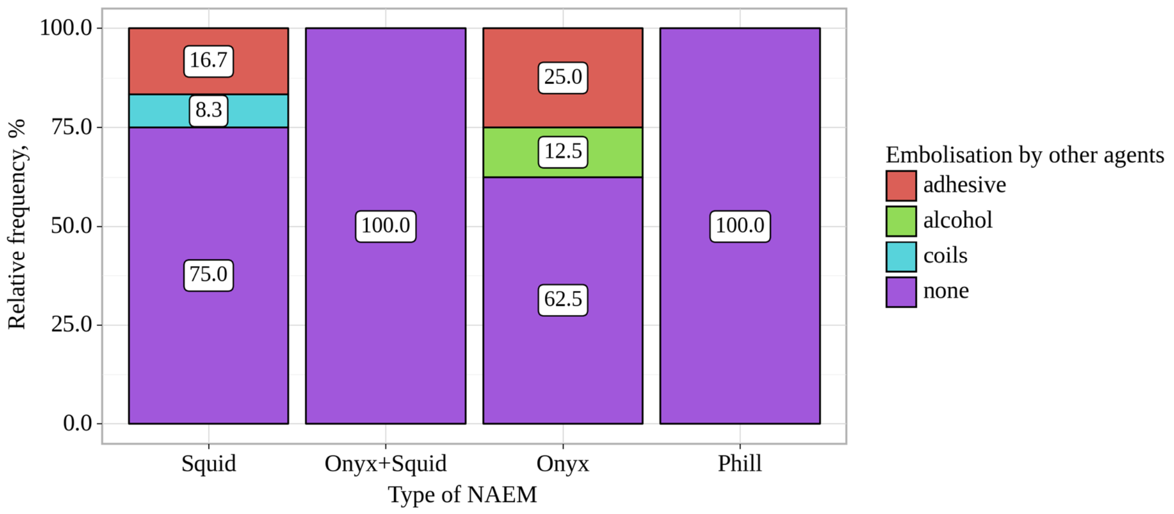

Figure S33 – Analysis of Embolisation by other agents conditioning on Type of NAEM

We performed analysis of Time of embolisation conditioning on Type of NAEM.

Table S31 – Analysis of Time of embolisation conditioning on Type of NAEM

Variable	Categories	Time of embolisation (min)			*p*
		Me	$Q_1 - Q_3$	n	
Type of NAEM	Squid	95	75 – 112	12	0.280
	Onyx+Squid	110	105 – 115	2	
	Onyx	78	70 – 95	8	
	Phill	120	120 – 120	1	

When comparing of Time of embolisation depending on Type of NAEM no statistically significant differences were revealed ($p = 0.280$) *(applied method: The Kruskal-Wallis test)*.

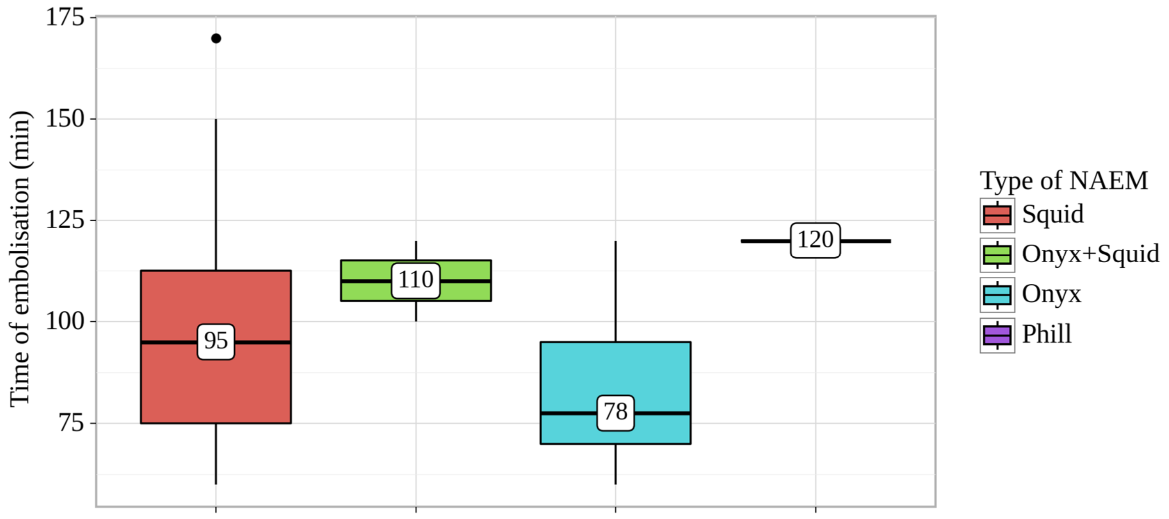

Figure S34 – Analysis of Time of embolisation conditioning on Type of NAEM

We performed analysis of Coils for NAEM conditioning on Type of NAEM.

Table S32 – Analysis of Coils for NAEM conditioning on Type of NAEM

Variable	Categories	Type of NAEM				*p*
		Squid	Onyx+Squid	Onyx	Phill	
Coils for NAEM	none	10 (83.3)	2 (100.0)	8 (100.0)	1 (100.0)	0.571
	+coils	2 (16.7)	0 (0.0)	0 (0.0)	0 (0.0)	

When comparing of Coils for NAEM depending on Type of NAEM there were no statistically significant differences ($p = 0.571$) *(applied method: Pearson's chi-square test)*.

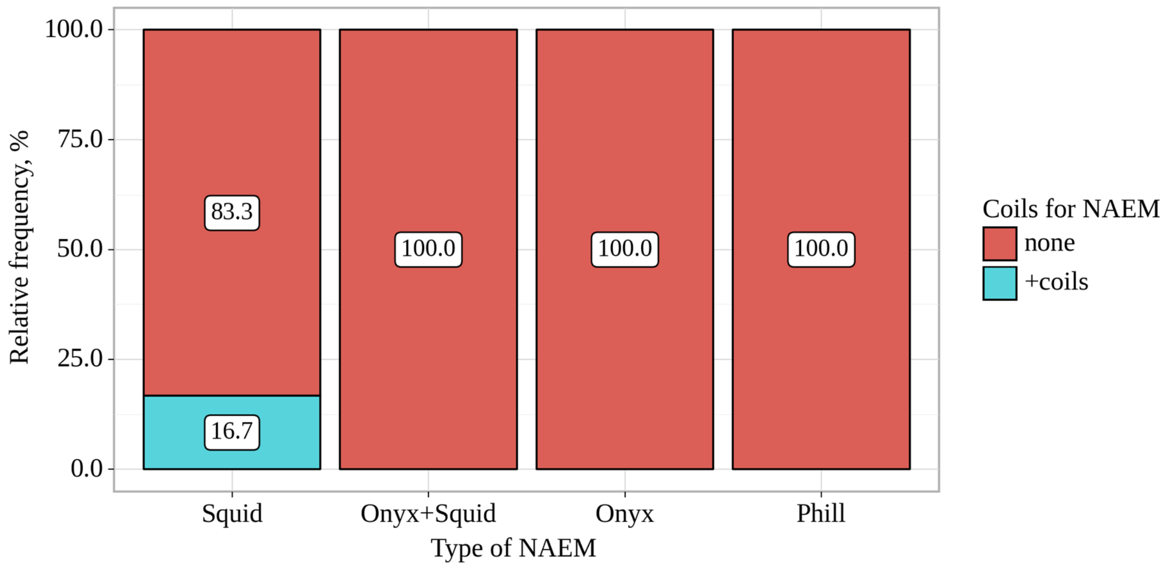

Figure S35 – Analysis of Coils for NAEM conditioning on Type of NAEM

Analysis of Radicality embolisation was performed conditioning on Type of NAEM.

Table S33 – Analysis of Radicality embolisation conditioning on Type of NAEM

Variable	Categories	Type of NAEM				*p*
		Squid	Onyx+Squid	Onyx	Phill	
Radicality embolisation	total	8 (66.7)	2 (100.0)	7 (87.5)	0 (0.0)	0.201
	subtotal	4 (33.3)	0 (0.0)	1 (12.5)	1 (100.0)	

When comparing of Radicality embolisation depending on Type of NAEM no statistically significant differences were revealed (p = 0.201) *(applied method: Pearson's chi-square test)*.

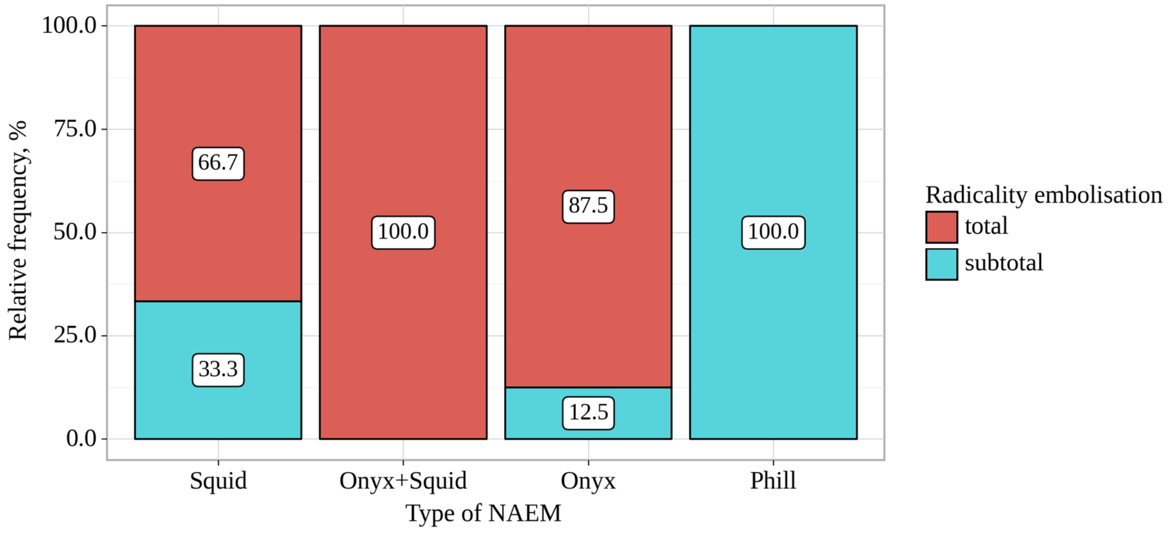

Figure S36 – Analysis of Radicality embolisation conditioning on Type of NAEM

Analysis of Type of catheter was performed conditioning on Type of NAEM.

Table S34 – Analysis of Type of catheter conditioning on Type of NAEM

Variable	Categories	Type of NAEM				*p*
		Squid	Onyx+Squid	Onyx	Phill	
Type of catheter	Scepter C, XC	10 (83.3)	2 (100.0)	8 (100.0)	1 (100.0)	0.571
	Headway	2 (16.7)	0 (0.0)	0 (0.0)	0 (0.0)	

When comparing of Type of catheter depending on Type of NAEM no statistically significant differences were revealed (p = 0.571) *(applied method: Pearson's chi-square test)*.

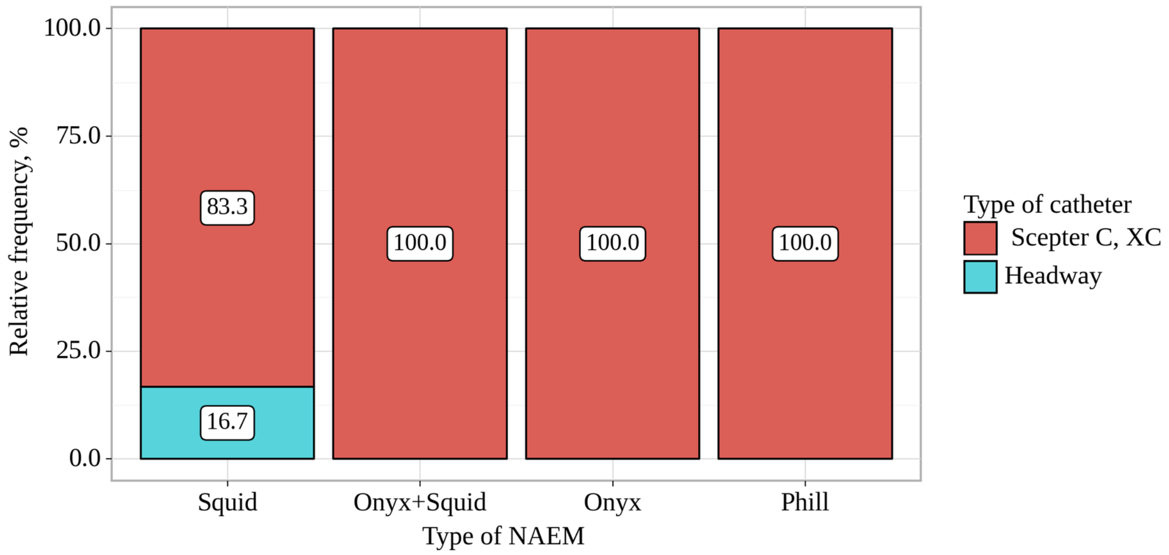

Figure S37 – Analysis of Type of catheter conditioning on Type of NAEM

Analysis of NAEM volume was performed conditioning on Type of NAEM.

Table S35 – Analysis of NAEM volume conditioning on Type of NAEM

Variable	Categories	NAEM volume (ml)			*p*
		Me	$Q_1 - Q_3$	n	
Type of NAEM	Squid	7.25	5.00 – 8.00	12	0.672
	Onyx+Squid	10.25	7.12 – 13.38	2	
	Onyx	7.25	6.00 – 8.00	8	
	Phill	9.00	9.00 – 9.00	1	

When comparing of NAEM volume depending on Type of NAEM there were no statistically significant differences ($p = 0.672$) *(applied method: The Kruskal-Wallis test)*.

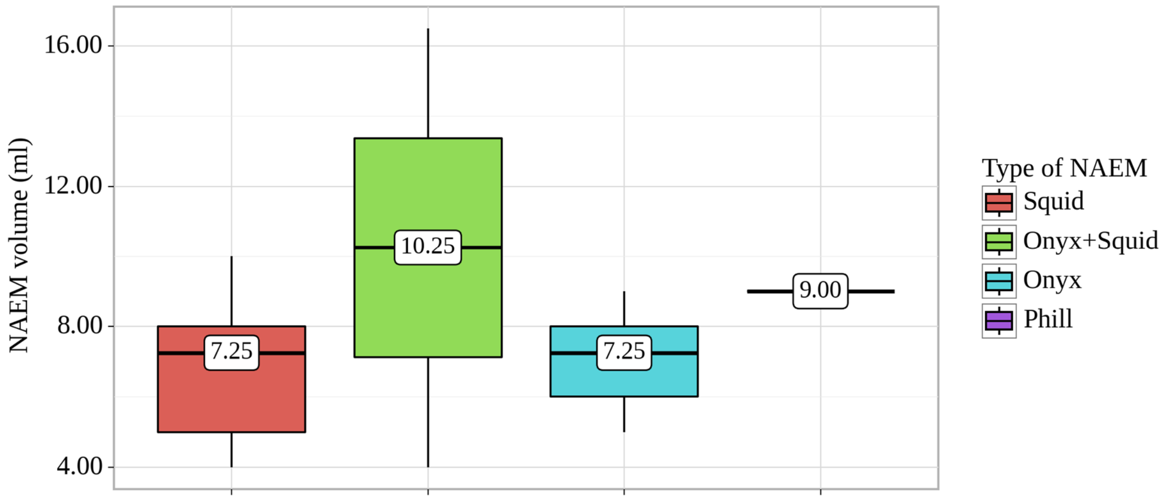

Figure S38 – Analysis of NAEM volume conditioning on Type of NAEM

Analysis of Type of Lesion was performed conditioning on Coils for NAEM.

Table S36 – Analysis of Type of Lesion conditioning on Coils for NAEM

Variable	Categories	Coils for NAEM		*p*
		none	+coils	
Type of Lesion	Arteriovenous Malformation of Face	5 (23.8)	0 (0.0)	0.303
	Carotid body paraganglioma	7 (33.3)	0 (0.0)	
	Jugular paraganglioma	9 (42.9)	2 (100.0)	

When comparing of Type of Lesion depending on Coils for NAEM no statistically significant differences were revealed ($p = 0.303$) *(applied method: Pearson's chi-square test)*.

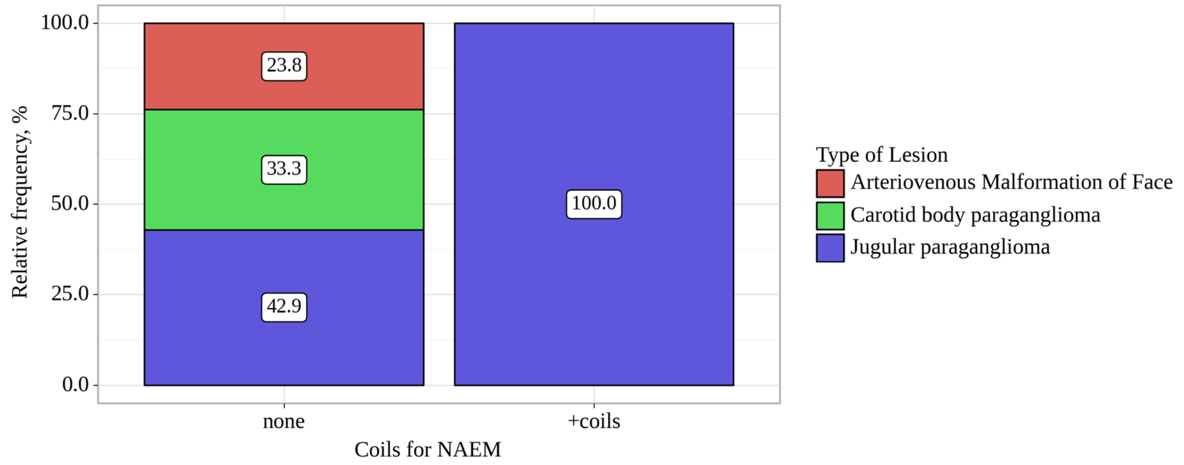

Figure S39 – Analysis of Type of Lesion conditioning on Coils for NAEM

Analysis of Time of embolisation was performed conditioning on Coils for NAEM.

Table S37 – Analysis of Time of embolisation conditioning on Coils for NAEM

Variable	Categories	Time of embolisation (min)			*p*
		Me	$Q_1 – Q_3$	n	
Coils for NAEM	none	90	75 – 120	21	0.350
	+coils	80	70 – 90	2	

When comparing of Time of embolisation depending on Coils for NAEM there were no statistically significant differences ($p = 0.350$) *(applied method: Mann-Whitney U-test)*.

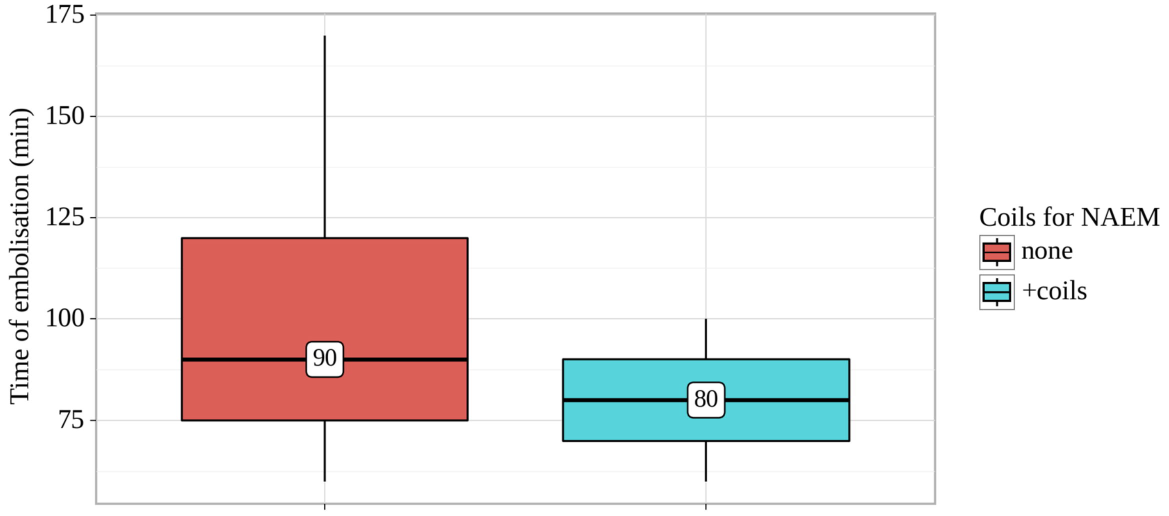

Figure S40 – Analysis of Time of embolisation conditioning on Coils for NAEM

Analysis of Type of NAEM was performed conditioning on Coils for NAEM.

Table S38 – Analysis of Type of NAEM conditioning on Coils for NAEM

Variable	Categories	Coils for NAEM		*p*
		none	+coils	
Type of NAEM	Squid	10 (47.6)	2 (100.0)	0.571
	Onyx+Squid	2 (9.5)	0 (0.0)	
	Onyx	8 (38.1)	0 (0.0)	
	Phill	1 (4.8)	0 (0.0)	

When comparing of Type of NAEM depending on Coils for NAEM there were no statistically significant differences ($p = 0.571$) *(applied method: Pearson's chi-square test)*.

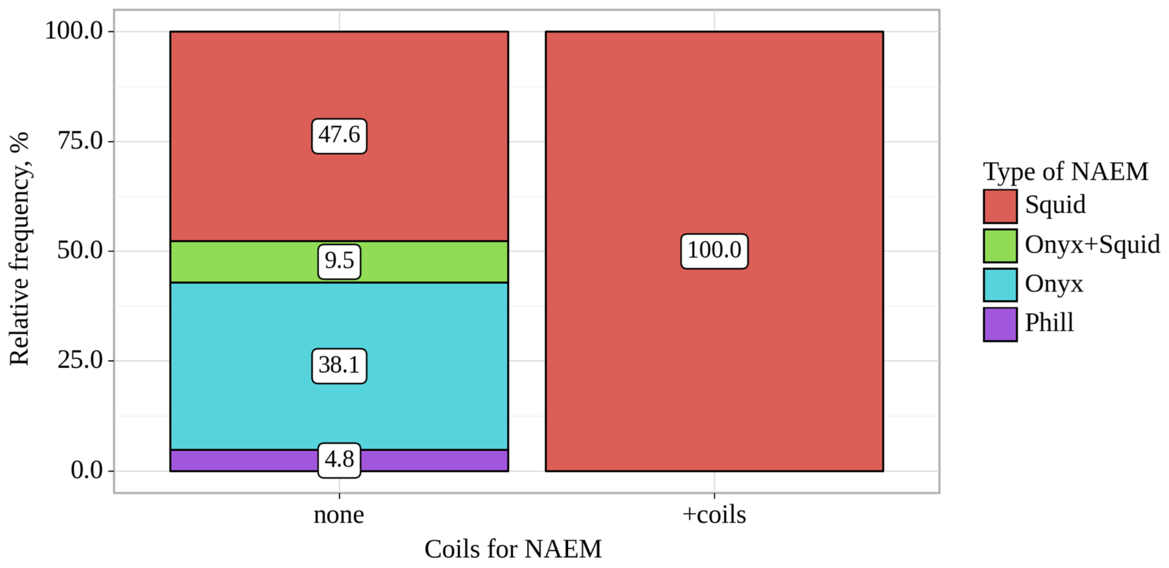

Figure S41 – Analysis of Type of NAEM conditioning on Coils for NAEM

Analysis of Radicality embolisation was performed conditioning on Coils for NAEM.

Table S39 – Analysis of Radicality embolisation conditioning on Coils for NAEM

Variable	Categories	Coils for NAEM		*p*
		none	+coils	
Radicality embolisation	total	17 (81.0)	0 (0.0)	0.059
	subtotal	4 (19.0)	2 (100.0)	

When comparing of Radicality embolisation depending on Coils for NAEM no statistically significant differences were revealed (p = 0.059) *(applied method: Fisher's exact test)*.

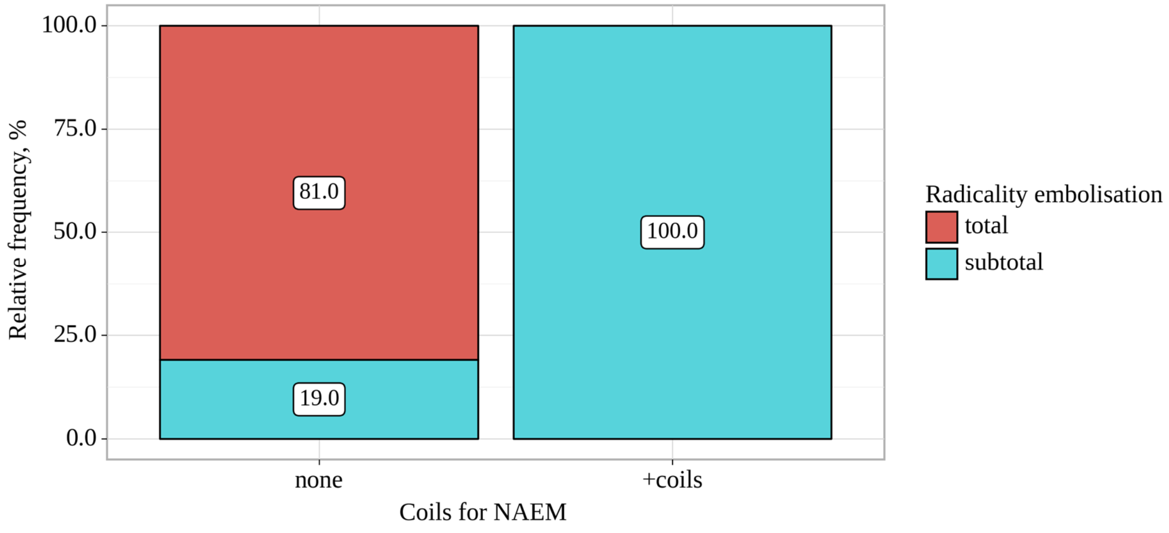

Figure S42 – Analysis of Radicality embolisation conditioning on Coils for NAEM

Odds of subtotal were 19.444 times greater in +coils group comparing with none, the relative difference in odds was not statistically significant (95% CI: 0.786 – 480.958).

Analysis of Type of catheter was performed conditioning on Coils for NAEM.

Table S40 – Analysis of Type of catheter conditioning on Coils for NAEM

Variable	Categories	Coils for NAEM		*p*
		none	+coils	
Type of catheter	Scepter C, XC	20 (95.2)	1 (50.0)	0.170
	Headway	1 (4.8)	1 (50.0)	

When comparing of Type of catheter depending on Coils for NAEM there were no statistically significant differences (p = 0.170) *(applied method: Fisher's exact test)*.

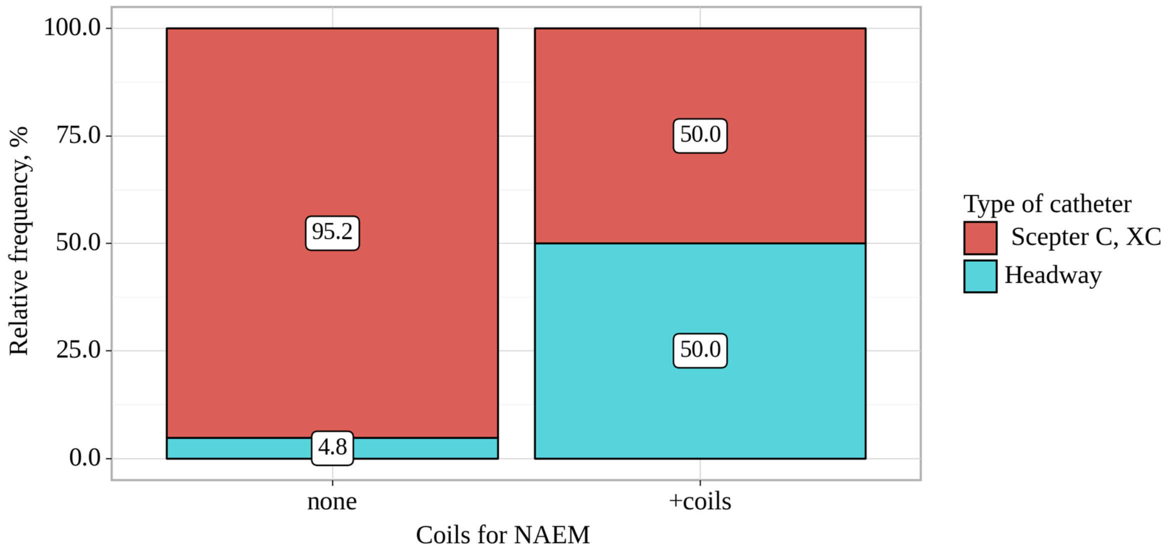

Figure S43 – Analysis of Type of catheter conditioning on Coils for NAEM

Odds of Headway were 20.000 times greater in +coils group comparing with none, the relative difference in odds was not statistically significant (95% CI: 0.652 – 613.182).

We performed analysis of NAEM volume conditioning on Coils for NAEM.

Table S41 – Analysis of NAEM volume conditioning on Coils for NAEM

Variable	Categories	NAEM volume (ml)			p
		Me	$Q_1 – Q_3$	n	
Coils for NAEM	none	7.50	6.00 – 8.00	21	0.621
	+coils	6.25	5.38 – 7.12	2	

When comparing of NAEM volume depending on Coils for NAEM there were no statistically significant differences (p = 0.621) *(applied method: Mann-Whitney U-test)*.

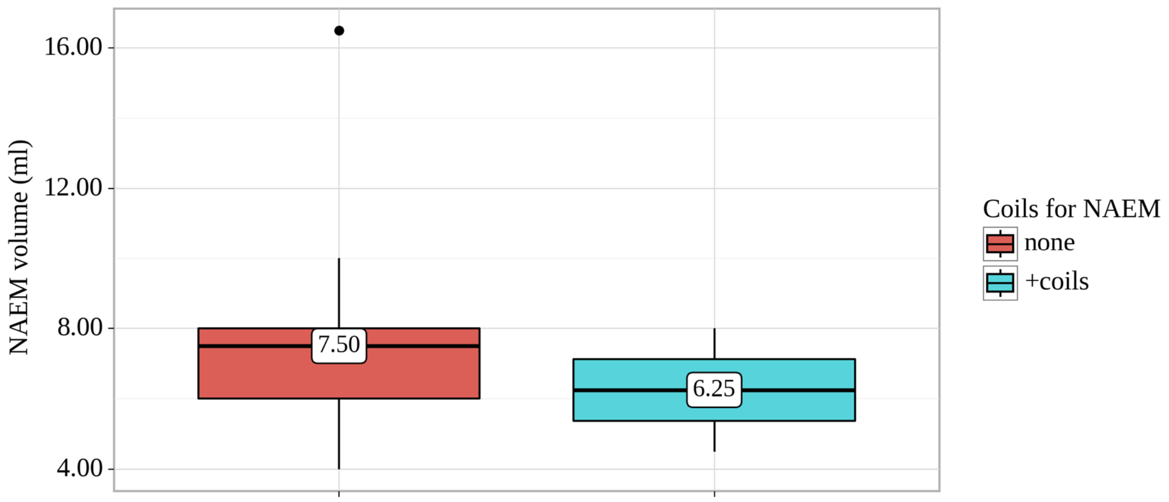

Figure S44 – Analysis of NAEM volume conditioning on Coils for NAEM

We performed analysis of Type of Lesion conditioning on Radicality embolisation.

Table S42 – Analysis of Type of Lesion conditioning on Radicality embolisation

Variable	Categories	Radicality embolisation		*p*
		total	subtotal	
Type of Lesion	Arteriovenous Malformation of Face	5 (29.4)	0 (0.0)	0.012*
	Carotid body paraganglioma	7 (41.2)	0 (0.0)	
	Jugular paraganglioma	5 (29.4)	6 (100.0)	

* – differences are statistically significant ($p < 0.05$)

Statistically significant differences were revealed when comparing of Type of Lesion depending on Radicality embolisation ($p = 0.012$) *(applied method: Pearson's chi-square test)*.

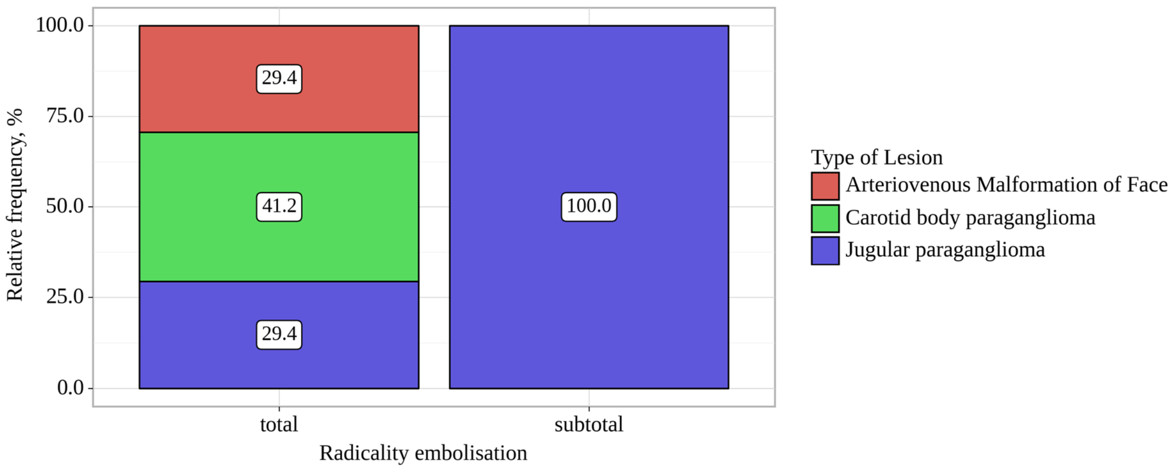

Figure S45 – Analysis of Type of Lesion conditioning on Radicality embolisation

Analysis of Number of treatment stages was performed conditioning on Radicality embolisation.

Table S43 – Analysis of Number of treatment stages conditioning on Radicality embolisation

Variable	Categories	Number of treatment stages			p
		Me	$Q_1 – Q_3$	n	
Radicality embolisation	total	2	1 – 3	17	0.042*
	subtotal	1	1 – 1	6	

* – differences are statistically significant ($p < 0.05$)

According to the data obtained when comparing of Number of treatment stages statistically significant differences were revealed depending on Radicality embolisation ($p = 0.042$) *(applied method: Mann-Whitney U-test)*.

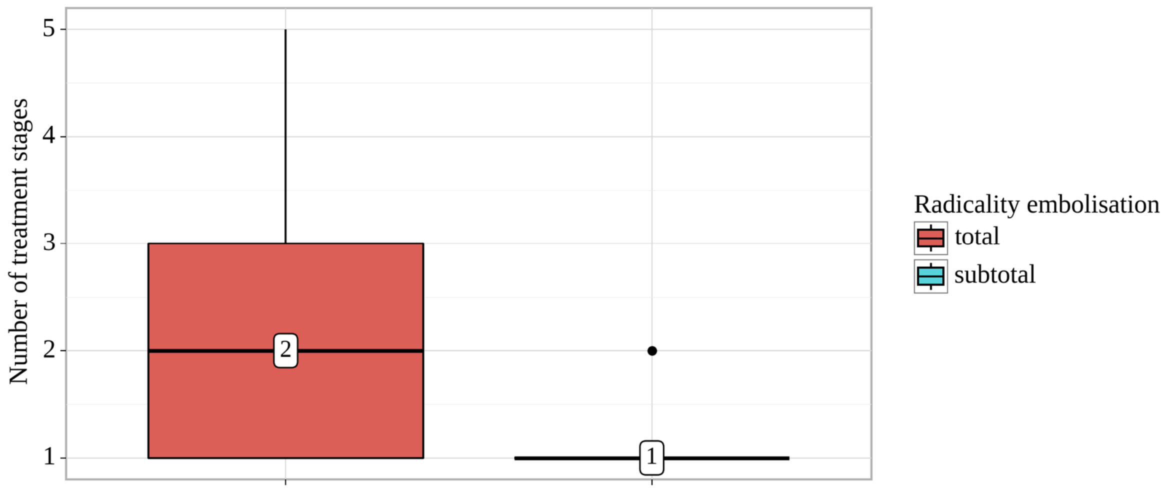

Figure S46 – Analysis of Number of treatment stages conditioning on Radicality embolisation

Analysis of Number of NAEM embolisation steps was performed conditioning on Radicality embolisation.

Table S44 – Analysis of Number of NAEM embolisation steps conditioning on Radicality embolisation

Variable	Categories	Number of NAEM embolisation steps			p
		Me	$Q_1 – Q_3$	n	
Radicality embolisation	total	1	1 – 2	17	0.145
	subtotal	1	1 – 1	6	

When comparing of Number of NAEM embolisation steps depending on Radicality embolisation no statistically significant differences were revealed ($p = 0.145$) *(applied method: Mann-Whitney U-test)*.

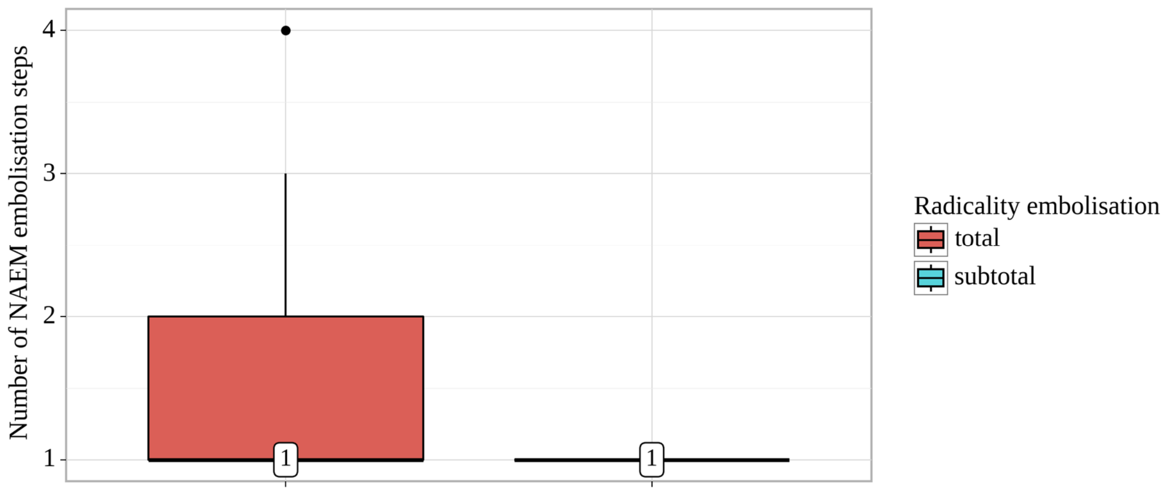

Figure S47 – Analysis of Number of NAEM embolisation steps conditioning on Radicality embolisation

We performed analysis of Embolisation by other agents conditioning on Radicality embolisation.

Table S45 – Analysis of Embolisation by other agents conditioning on Radicality embolisation

Variable	Categories	Radicality embolisation		*p*
		total	subtotal	
Embolisation by other agents	adhesive	4 (23.5)	0 (0.0)	0.413
	alcohol	1 (5.9)	0 (0.0)	
	coils	1 (5.9)	0 (0.0)	
	none	11 (64.7)	6 (100.0)	

When comparing of Embolisation by other agents depending on Radicality embolisation no statistically significant differences were revealed ($p = 0.413$) *(applied method: Pearson's chi-square test)*.

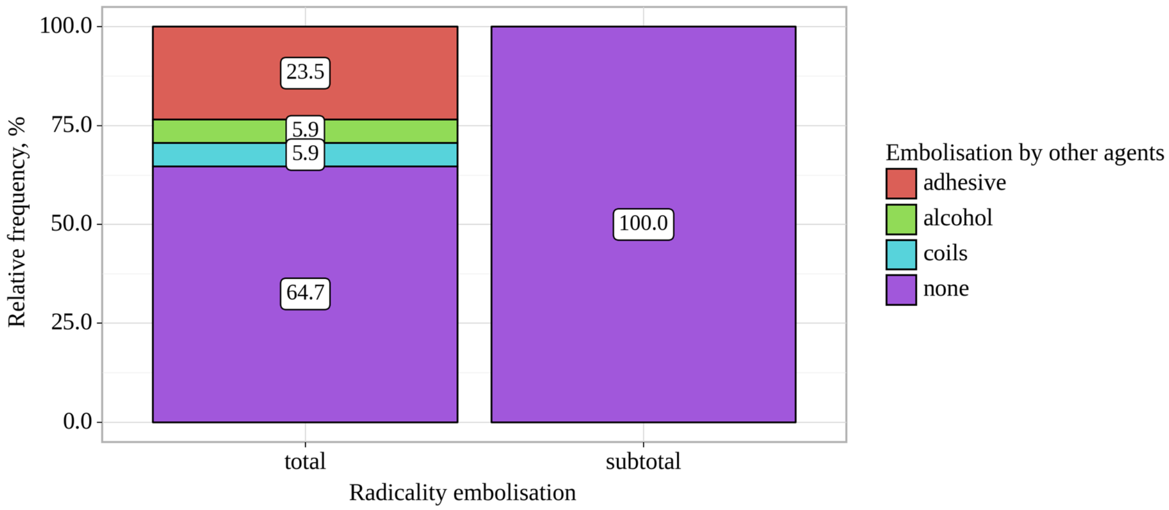

Figure S48 – Analysis of Embolisation by other agents conditioning on Radicality embolisation

Analysis of Time of embolisation was performed conditioning on Radicality embolisation.

Table S46 – Analysis of Time of embolisation conditioning on Radicality embolisation

Variable	Categories	Time of embolisation (min)			*p*
		M ± SD	95% CI	n	
Radicality embolisation	total	98 ± 30	82 – 113	17	0.801
	subtotal	94 ± 24	69 – 120	6	

When comparing of Time of embolisation depending on Radicality embolisation no statistically significant differences were revealed (*p* = 0.801) *(applied method: Student's t-test)*.

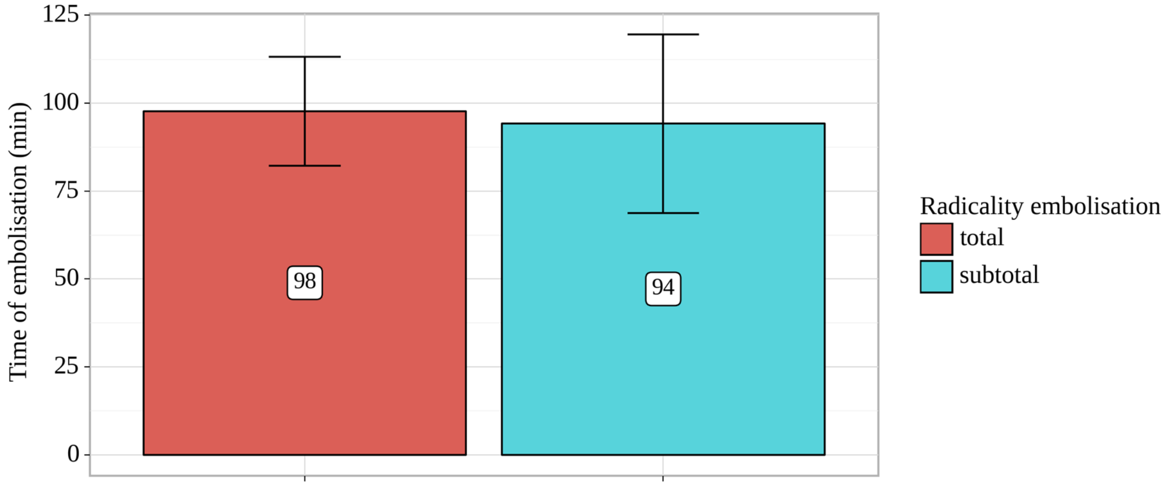

Figure S49 – Analysis of Time of embolisation conditioning on Radicality embolisation

We performed analysis of Type of NAEM conditioning on Radicality embolisation.

Table S47 – Analysis of Type of NAEM conditioning on Radicality embolisation

Variable	Categories	Radicality embolisation		*p*
		total	subtotal	
Type of NAEM	Squid	8 (47.1)	4 (66.7)	0.201
	Onyx+Squid	2 (11.8)	0 (0.0)	
	Onyx	7 (41.2)	1 (16.7)	
	Phill	0 (0.0)	1 (16.7)	

When comparing of Type of NAEM depending on Radicality embolisation no statistically significant differences were revealed (*p* = 0.201) *(applied method: Pearson's chi-square test)*.

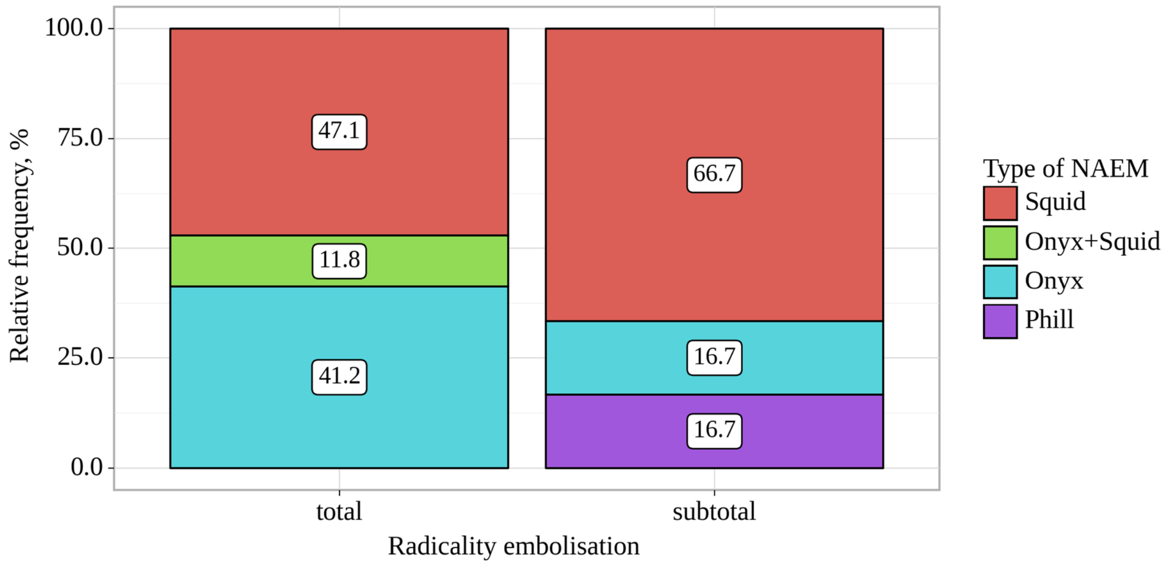

Figure S50 – Analysis of Type of NAEM conditioning on Radicality embolisation

Analysis of Coils for NAEM was performed conditioning on Radicality embolisation.

Table S48 – Analysis of Coils for NAEM conditioning on Radicality embolisation

Variable	Categories	Radicality embolisation		*p*
		total	subtotal	
Coils for NAEM	none	17 (100.0)	4 (66.7)	0.059
	+coils	0 (0.0)	2 (33.3)	

When comparing of Coils for NAEM depending on Radicality embolisation there were no statistically significant differences ($p = 0.059$) *(applied method: Fisher's exact test)*.

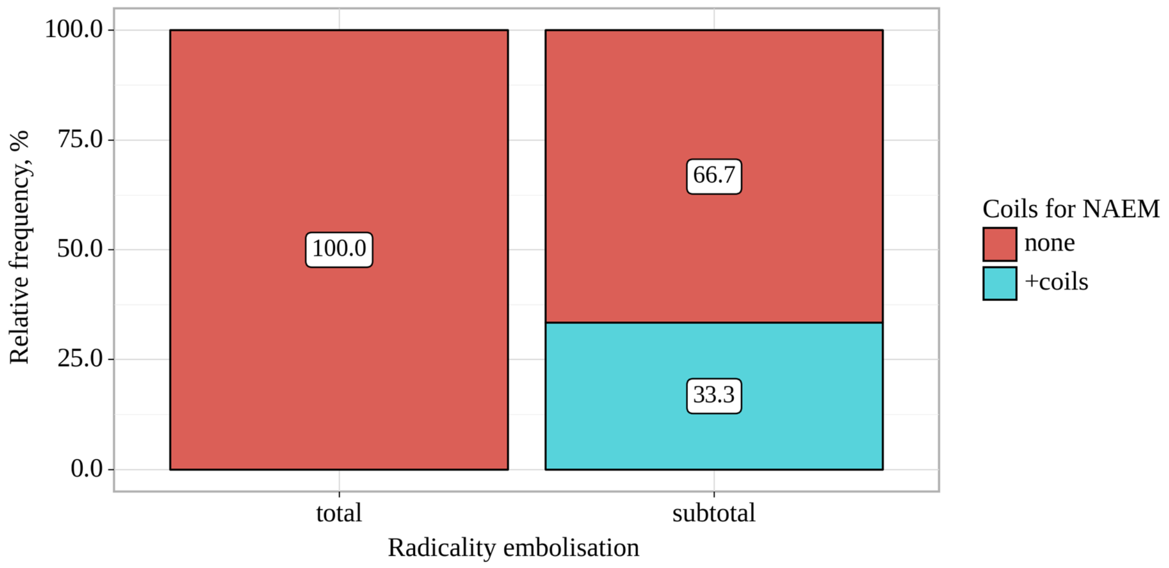

Figure S51 – Analysis of Coils for NAEM conditioning on Radicality embolisation

Odds of +coils were 19.444 times greater in subtotal group comparing with total, the relative difference in odds was not statistically significant (95% CI: 0.786 – 480.958).

Analysis of Type of catheter was performed conditioning on Radicality embolisation.

Table S49 – Analysis of Type of catheter conditioning on Radicality embolisation

Variable	Categories	Radicality embolisation		*p*
		total	subtotal	
Type of catheter	Scepter C, XC	17 (100.0)	4 (66.7)	0.059
	Headway	0 (0.0)	2 (33.3)	

When comparing of Type of catheter depending on Radicality embolisation there were no statistically significant differences ($p = 0.059$) *(applied method: Fisher's exact test)*.

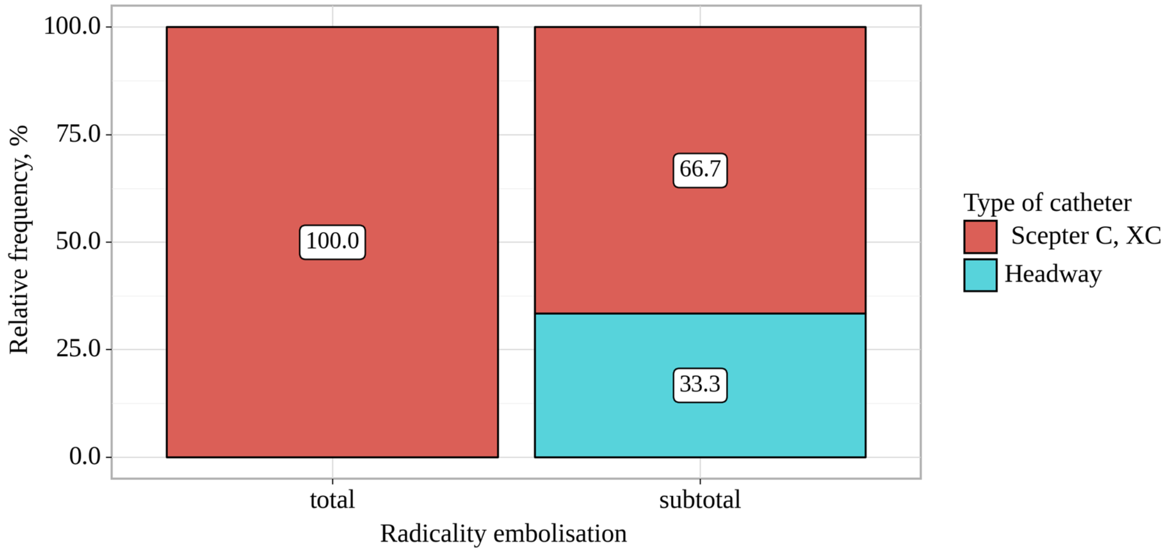

Figure S52 – Analysis of Type of catheter conditioning on Radicality embolisation

Odds of Headway were 19.444 times greater in subtotal group comparing with total, the relative difference in odds was not statistically significant (95% CI: 0.786 – 480.958).

Analysis of NAEM volume was performed conditioning on Radicality embolisation.

Table S50 – Analysis of NAEM volume conditioning on Radicality embolisation

Variable	Categories	NAEM volume (ml)			*p*
		Me	$Q_1 – Q_3$	n	
Radicality embolisation	total	7.00	6.00 – 8.00	17	0.888
	subtotal	7.75	5.25 – 8.75	6	

When comparing of NAEM volume depending on Radicality embolisation no statistically significant differences were revealed (*p* = 0.888) *(applied method: Mann-Whitney U-test)*.

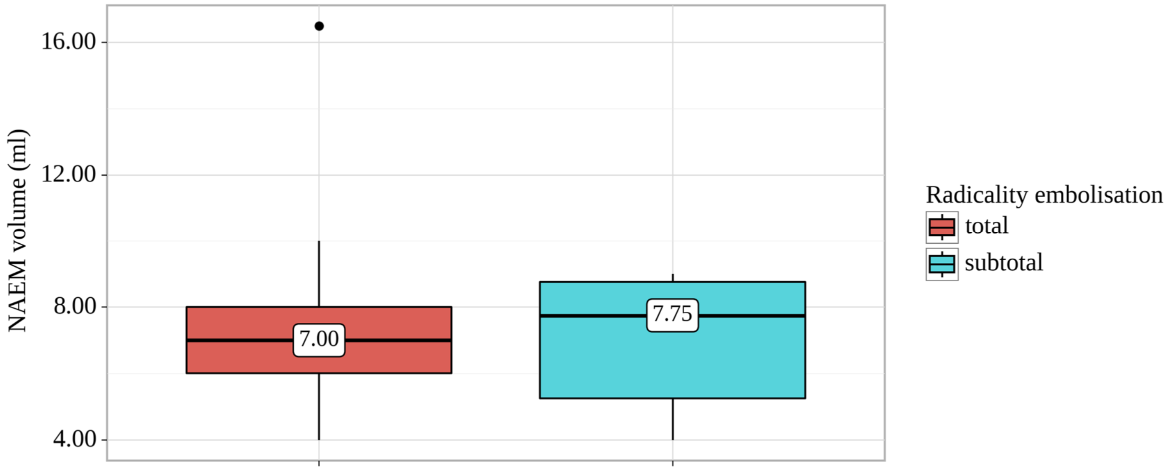

Figure S53 – Analysis of NAEM volume conditioning on Radicality embolisation

Analysis of Type of Lesion was performed conditioning on Type of catheter.

Table S51 – Analysis of Type of Lesion conditioning on Type of catheter

Variable	Categories	Type of catheter		*p*
		Scepter C, XC	Headway	
Type of Lesion	Arteriovenous Malformation of Face	5 (23.8)	0 (0.0)	0.303
	Carotid body paraganglioma	7 (33.3)	0 (0.0)	
	Jugular paraganglioma	9 (42.9)	2 (100.0)	

When comparing of Type of Lesion depending on Type of catheter no statistically significant differences were revealed (*p* = 0.303) *(applied method: Pearson's chi-square test)*.

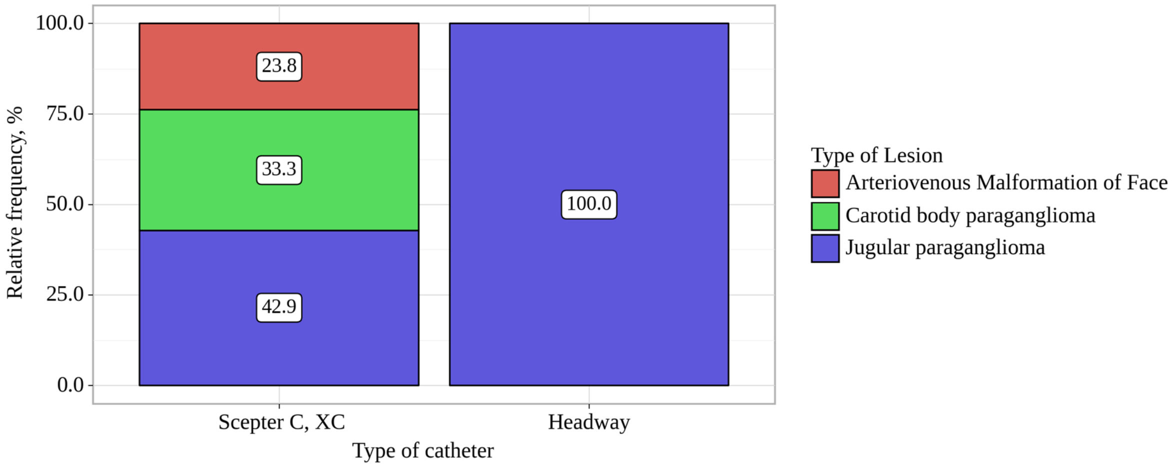

Figure S54 – Analysis of Type of Lesion conditioning on Type of catheter

Analysis of Number of treatment stages was performed conditioning on Type of catheter.

Table S52 – Analysis of Number of treatment stages conditioning on Type of catheter

Variable	Categories	Number of treatment stages			*p*
		Me	$Q_1 – Q_3$	n	
Type of catheter	Scepter C, XC	2	1 – 2	21	0.159
	Headway	1	1 – 1	2	

When comparing of Number of treatment stages depending on Type of catheter no statistically significant differences were revealed ($p = 0.159$) *(applied method: Mann-Whitney U-test)*.

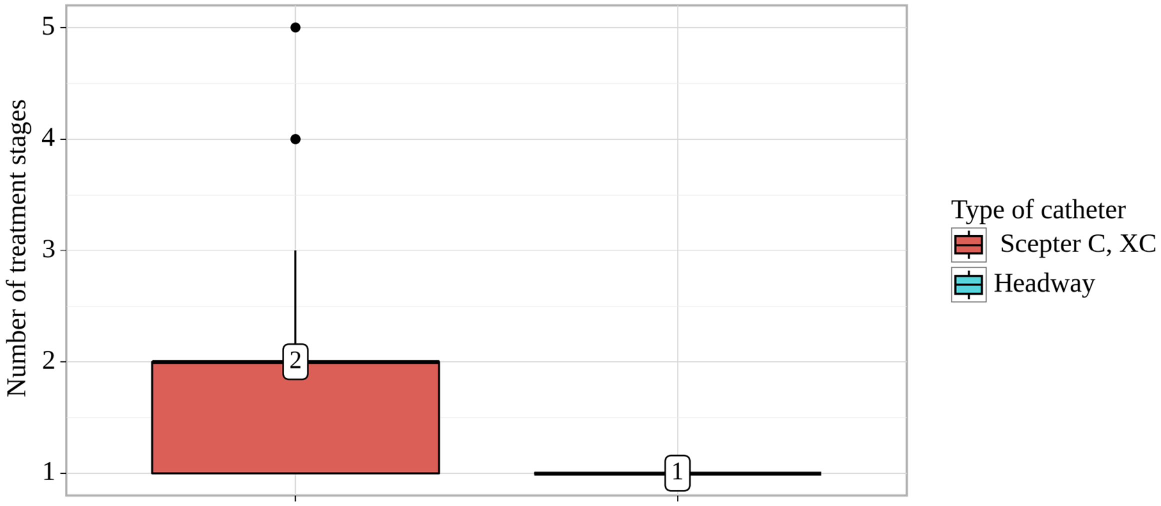

Figure S55 – Analysis of Number of treatment stages conditioning on Type of catheter

Analysis of Number of NAEM embolisation steps was performed conditioning on Type of catheter.

Table S53 – Analysis of Number of NAEM embolisation steps conditioning on Type of catheter

Variable	Categories	Number of NAEM embolisation steps			*p*
		Me	$Q_1 – Q_3$	n	
Type of catheter	Scepter C, XC	1	1 – 1	21	0.449
	Headway	1	1 – 1	2	

When comparing of Number of NAEM embolisation steps depending on Type of catheter there were no statistically significant differences ($p = 0.449$) *(applied method: Mann-Whitney U-test)*.

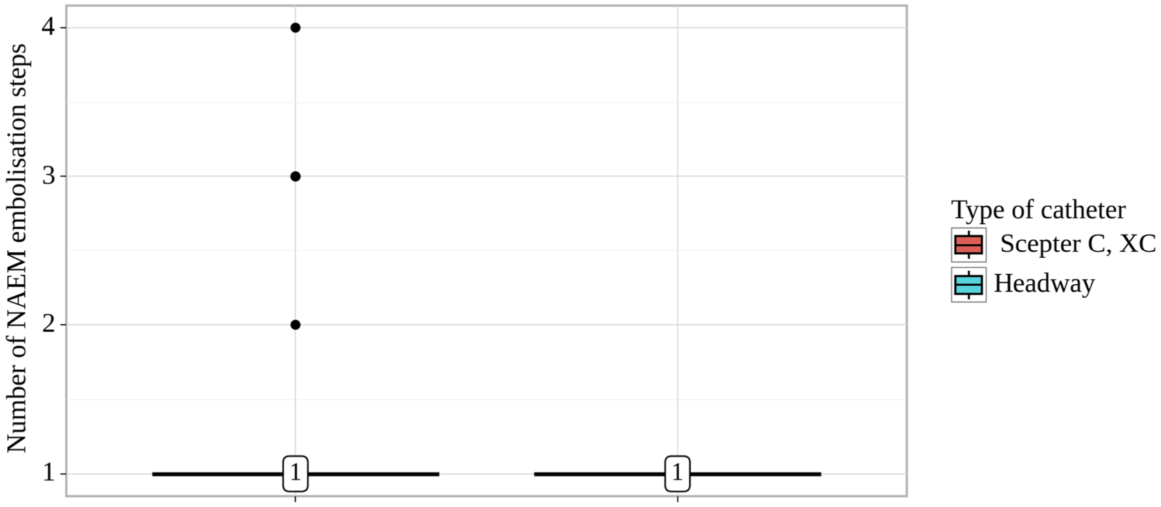

Figure S56 – Analysis of Number of NAEM embolisation steps conditioning on Type of catheter

We performed analysis of Embolisation by other agents conditioning on Type of catheter.

Table S54 – Analysis of Embolisation by other agents conditioning on Type of catheter

Variable	Categories	Type of catheter		*p*
		Scepter C, XC	Headway	
Embolisation by other agents	adhesive	4 (19.0)	0 (0.0)	0.856
	alcohol	1 (4.8)	0 (0.0)	
	coils	1 (4.8)	0 (0.0)	
	none	15 (71.4)	2 (100.0)	

When comparing of Embolisation by other agents depending on Type of catheter no statistically significant differences were revealed ($p = 0.856$) *(applied method: Pearson's chi-square test)*.

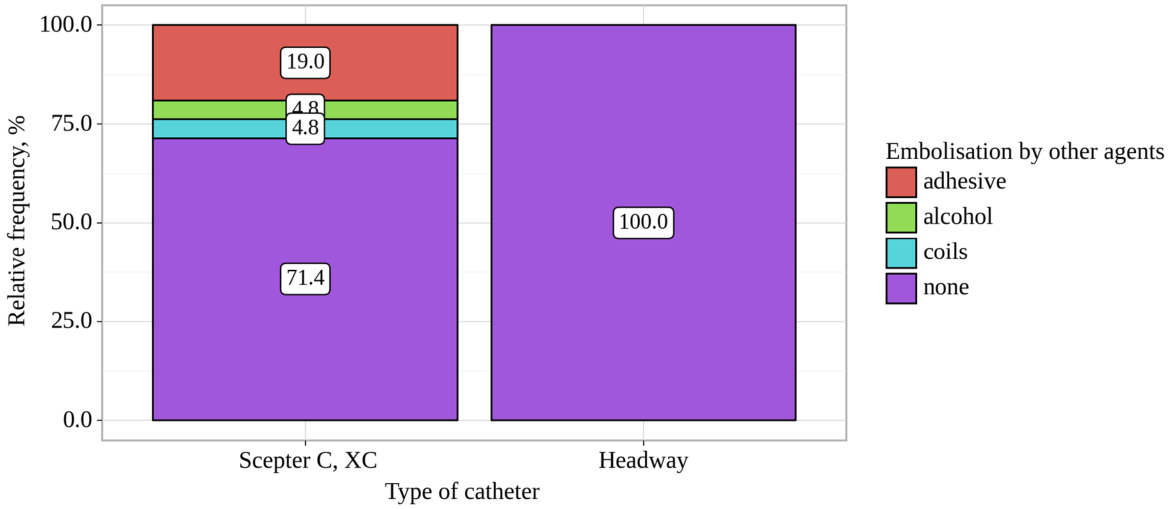

Figure S57 – Analysis of Embolisation by other agents conditioning on Type of catheter

We performed analysis of Time of embolisation conditioning on Type of catheter.

Table S55 – Analysis of Time of embolisation conditioning on Type of catheter

Variable	Categories	Time of embolisation (min)			*p*
		Me	$Q_1 - Q_3$	n	
Type of catheter	Scepter C, XC	100	75 – 120	21	0.206
	Headway	75	68 – 82	2	

When comparing of Time of embolisation depending on Type of catheter there were no statistically significant differences ($p = 0.206$) *(applied method: Mann-Whitney U-test)*.

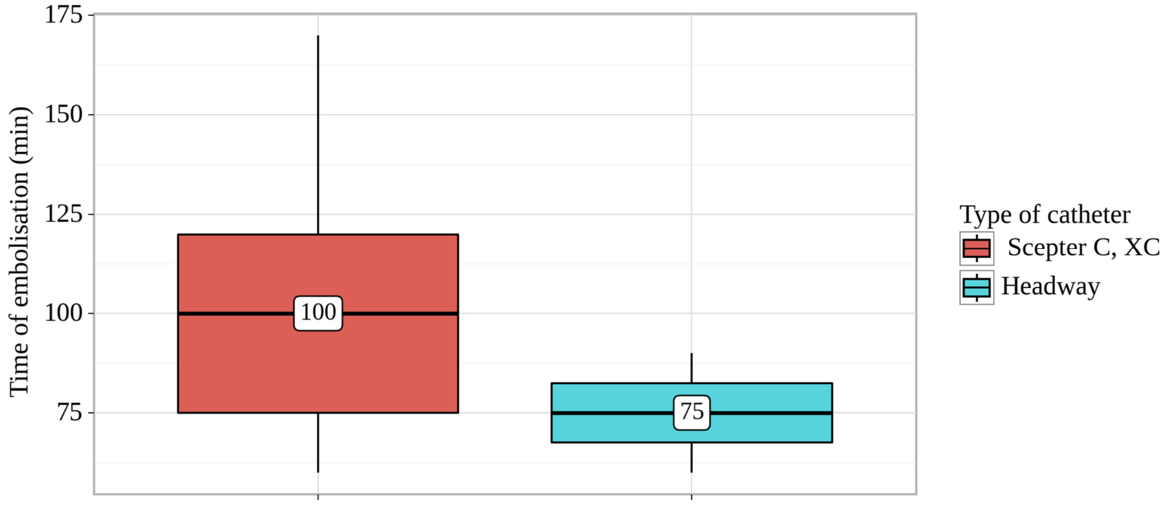

Figure S58 – Analysis of Time of embolisation conditioning on Type of catheter

We performed analysis of Type of NAEM conditioning on Type of catheter.

Table S56 – Analysis of Type of NAEM conditioning on Type of catheter

Variable	Categories	Type of catheter		p
		Scepter C, XC	Headway	
Type of NAEM	Squid	10 (47.6)	2 (100.0)	0.571
	Onyx+Squid	2 (9.5)	0 (0.0)	
	Onyx	8 (38.1)	0 (0.0)	
	Phill	1 (4.8)	0 (0.0)	

When comparing of Type of NAEM depending on Type of catheter there were no statistically significant differences ($p = 0.571$) *(applied method: Pearson's chi-square test)*.

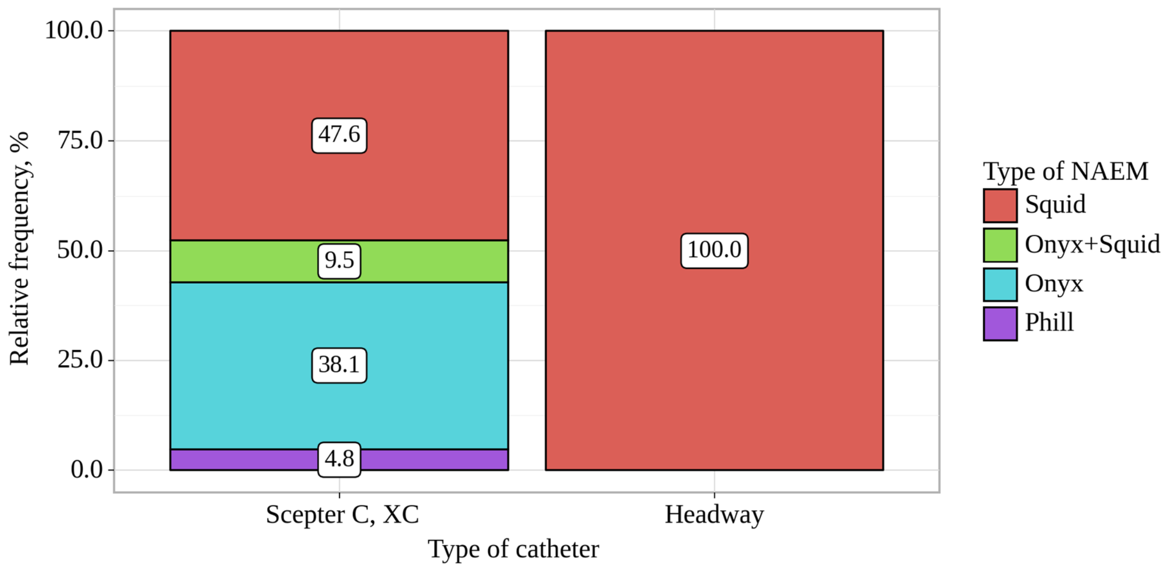

Figure S59 – Analysis of Type of NAEM conditioning on Type of catheter

We performed analysis of Coils for NAEM conditioning on Type of catheter.

Table S57 – Analysis of Coils for NAEM conditioning on Type of catheter

Variable	Categories	Type of catheter		p
		Scepter C, XC	Headway	
Coils for NAEM	none	20 (95.2)	1 (50.0)	0.170
	+coils	1 (4.8)	1 (50.0)	

When comparing of Coils for NAEM depending on Type of catheter no statistically significant differences were revealed ($p = 0.170$) *(applied method: Fisher's exact test)*.

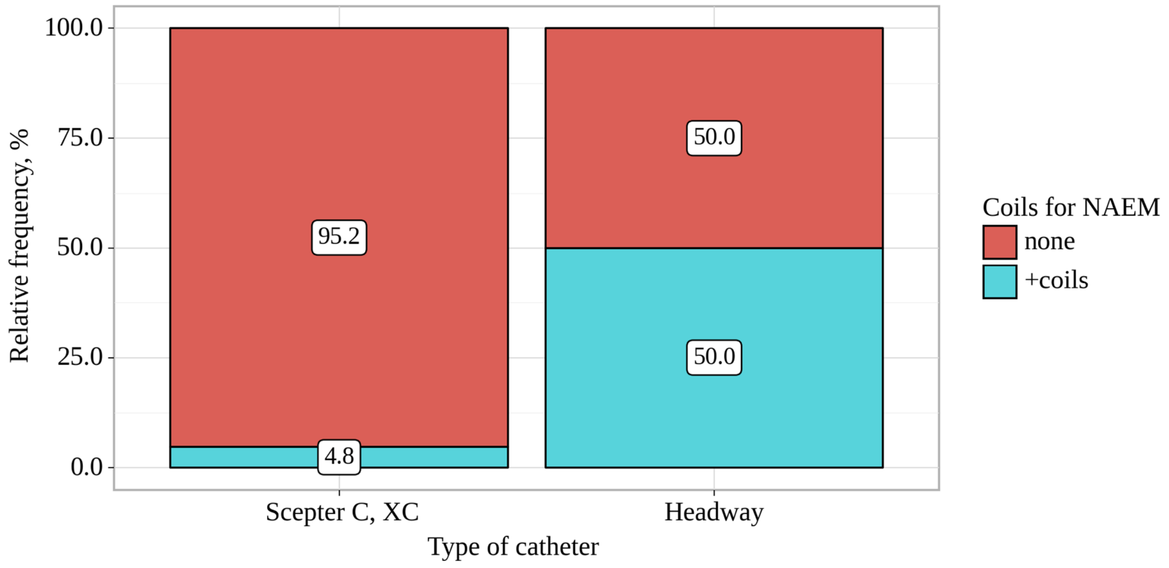

Figure S60 – Analysis of Coils for NAEM conditioning on Type of catheter

Odds of +coils were 20.000 times greater in Headway group comparing with Scepter C, XC, the relative difference in odds was not statistically significant (95% CI: 0.652 – 613.182).

We performed analysis of Radicality embolisation conditioning on Type of catheter.

Table S58 – Analysis of Radicality embolisation conditioning on Type of catheter

Variable	Categories	Type of catheter		*p*
		Scepter C, XC	Headway	
Radicality embolisation	total	17 (81.0)	0 (0.0)	0.059
	subtotal	4 (19.0)	2 (100.0)	

When comparing of Radicality embolisation depending on Type of catheter no statistically significant differences were revealed ($p = 0.059$) *(applied method: Fisher's exact test)*.

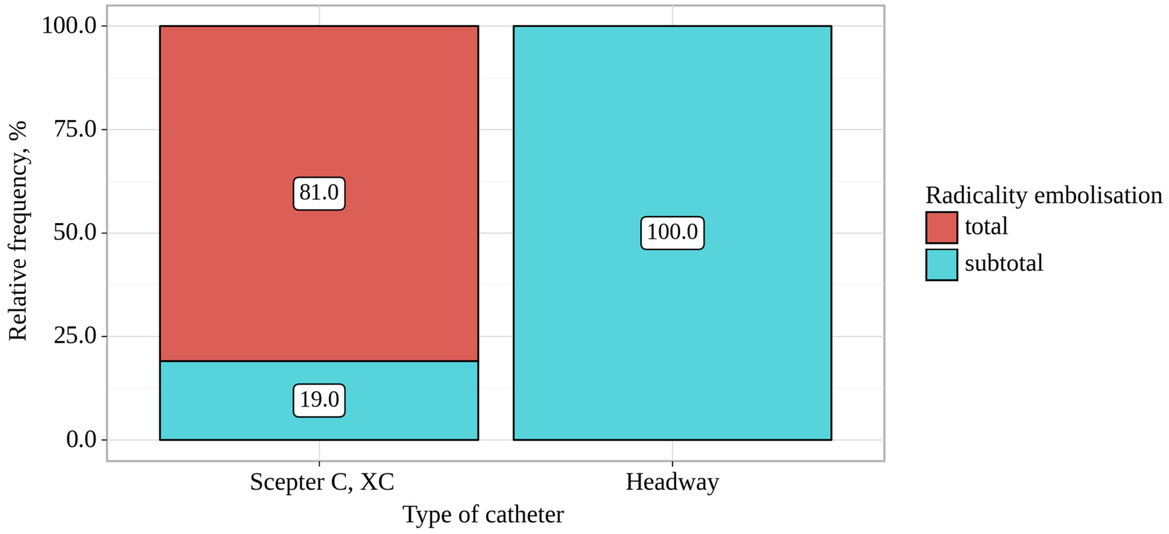

Figure S61 – Analysis of Radicality embolisation conditioning on Type of catheter

Odds of subtotal were 19.444 times greater in Headway group comparing with Scepter C, XC, the relative difference in odds was not statistically significant (95% CI: 0.786 – 480.958).

We performed analysis of NAEM volume conditioning on Type of catheter.

Table S59 – Analysis of NAEM volume conditioning on Type of catheter

Variable	Categories	NAEM volume (ml)			*p*
		Me	$Q_1 – Q_3$	n	
Type of catheter	Scepter C, XC	7.50	6.00 – 8.00	21	0.032*
	Headway	4.25	4.12 – 4.38	2	

* – differences are statistically significant ($p < 0.05$)

Statistically significant differences were revealed when comparing of NAEM volume depending on Type of catheter ($p = 0.032$) *(applied method: Mann-Whitney U-test)*.

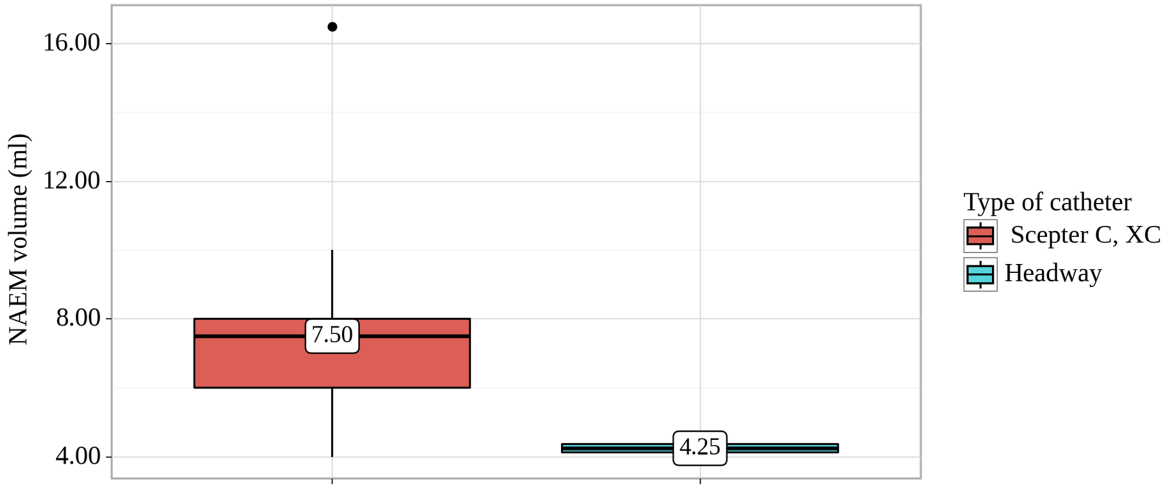

Figure S62 – Analysis of NAEM volume conditioning on Type of catheter

Correlation analysis of the association between NAEM volume and Time of embolisation was performed.

Table S60 – Results of the correlation analysis of the association between NAEM volume and Time of embolisation

Variable	Correlation characteristics		
	ρ	Strength of the association assesed using Chaddock scale	*p*
NAEM volume – Time of embolisation	0.395	Moderate	0.062

A moderate correlation positive association between Time of embolisation and NAEM volume was estimated.

Observed dependence of Time of embolisation from NAEM volume is described by a linear regression equation:

$$Y_{\text{Time of embolisation}} = 2.631 \times X_{\text{NAEM volume}} + 77.461$$

With an 1 ml increase of NAEM volume 2.631 min change of Time of embolisation should be expected. According to the coefficient of determination R^2 of the resulting model, 6.0% of the observed variance of Time of embolisation were explained..

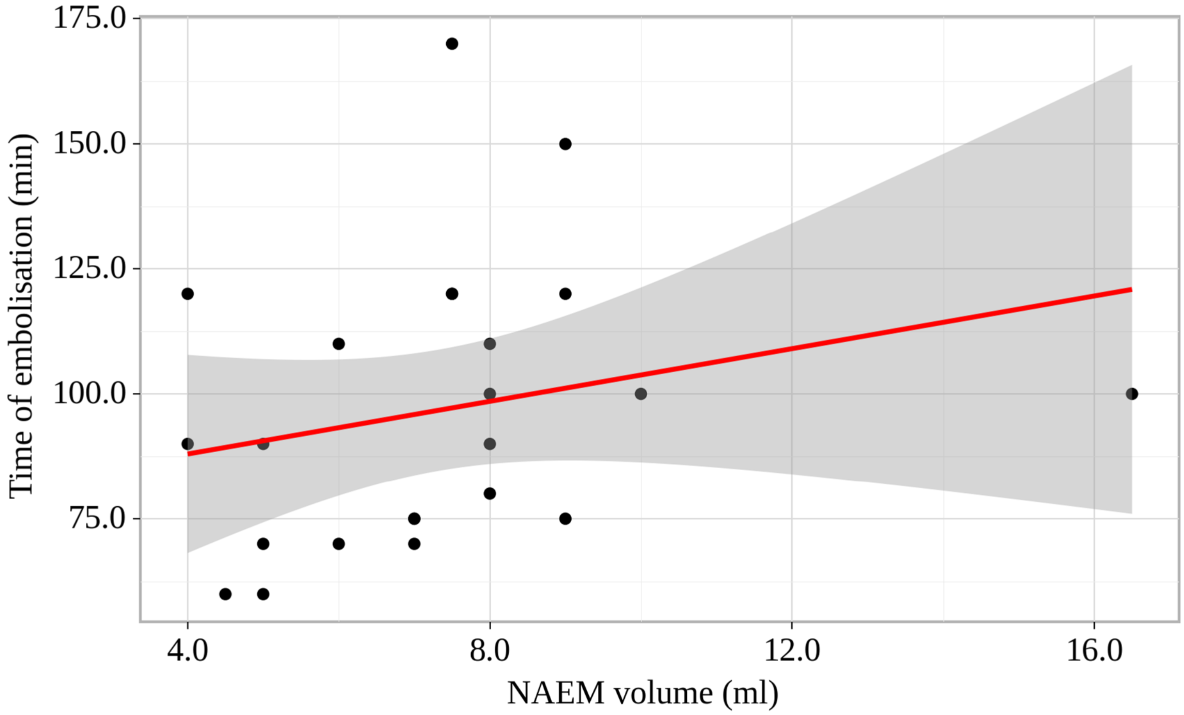

Figure S63 – Regression line characterizing the dependence of Time of embolisation from NAEM volume

Analysis of NAEM volume was performed conditioning on Type of NAEM.

Table S61 – Analysis of NAEM volume conditioning on Type of NAEM

Variable	Categories	NAEM volume (ml)			*p*
		Me	$Q_1 – Q_3$	n	
Type of NAEM	Squid	7.25	5.00 – 8.00	12	0.672
	Onyx+Squid	10.25	7.12 – 13.38	2	
	Onyx	7.25	6.00 – 8.00	8	
	Phill	9.00	9.00 – 9.00	1	

When comparing of NAEM volume depending on Type of NAEM there were no statistically significant differences (p = 0.672) *(applied method: The Kruskal-Wallis test)*.

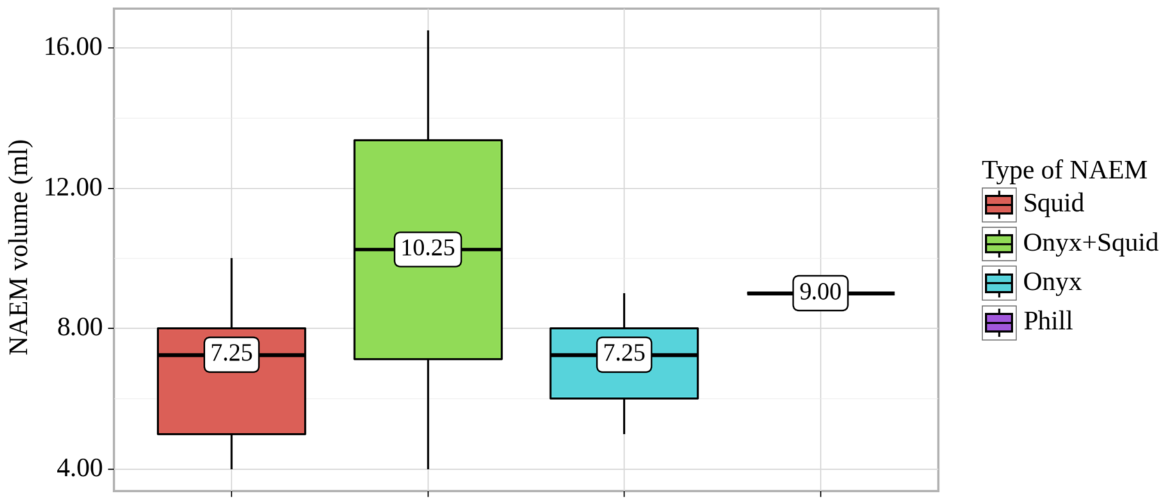

Figure S64 – Analysis of NAEM volume conditioning on Type of NAEM

Analysis of NAEM volume was performed conditioning on Coils for NAEM.

Table S62 – Analysis of NAEM volume conditioning on Coils for NAEM

Variable	Categories	NAEM volume (ml)			*p*
		Me	$Q_1 – Q_3$	n	
Coils for NAEM	none	7.50	6.00 – 8.00	21	0.621
	+coils	6.25	5.38 – 7.12	2	

When comparing of NAEM volume depending on Coils for NAEM no statistically significant differences were revealed ($p = 0.621$) *(applied method: Mann-Whitney U-test)*.

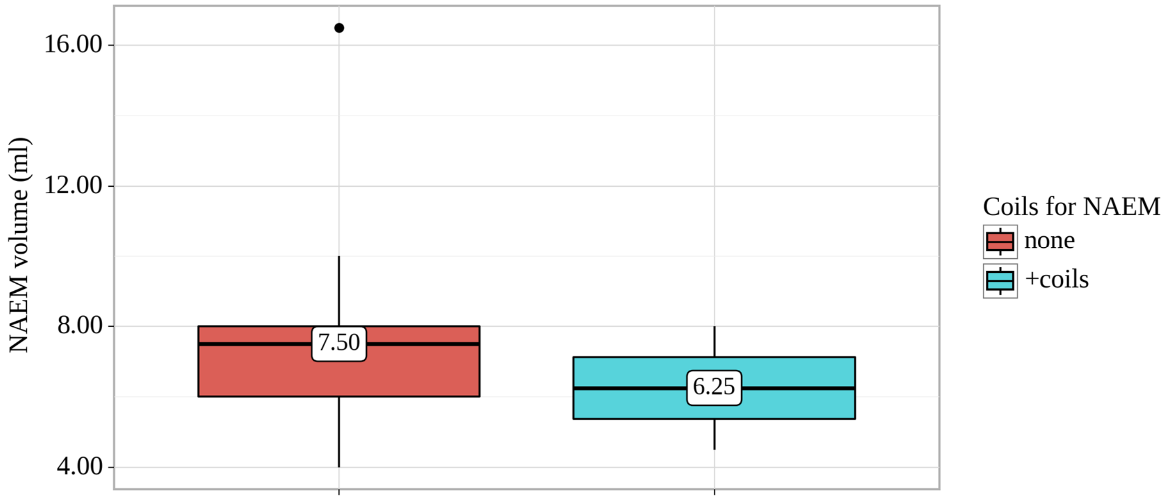

Figure S65 – Analysis of NAEM volume conditioning on Coils for NAEM

When evaluating the dependence of the probability of +coils on the NAEM volume using the ROC analysis, the following curve was obtained.

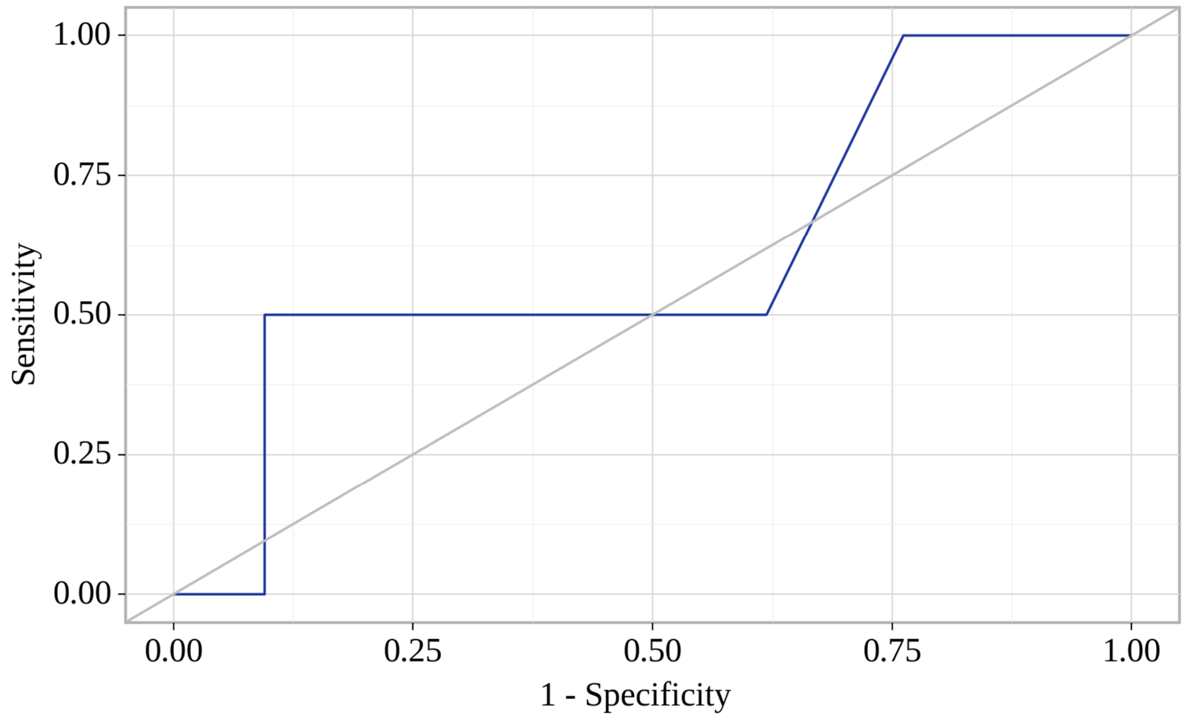

Figure S66 – ROC-curve characterizing the dependence of the probability Coils for NAEM on NAEM volume

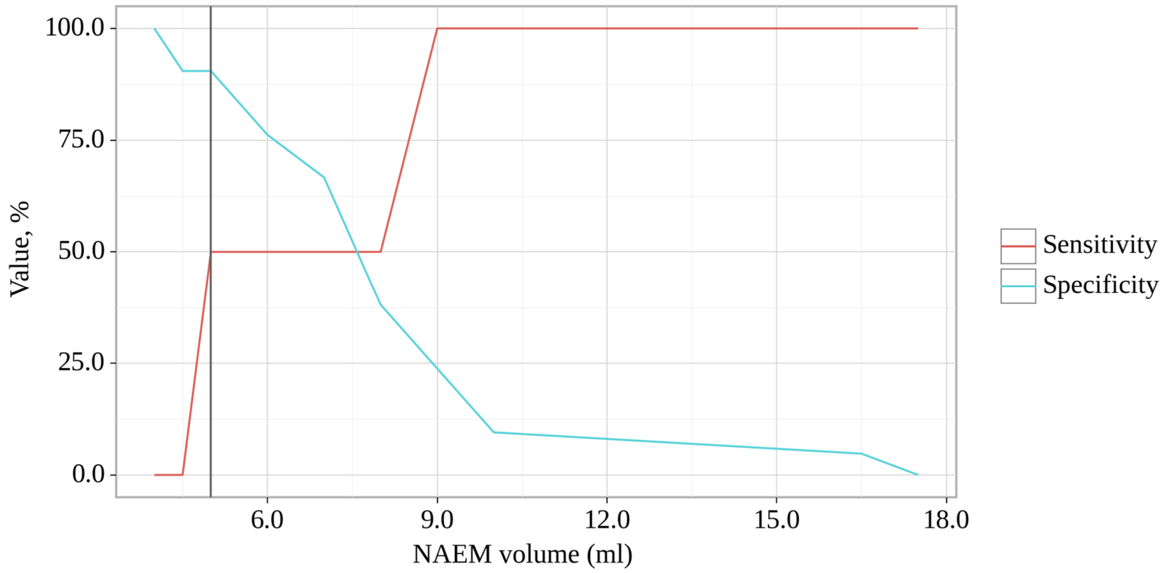

Figure S67 - Analysis of the sensitivity and specificity of Coils for NAEM depending on NAEM volume

Table S63 – Threshold NAEM volume

Threshold	Sensitivity (Se), %	Specificity (Sp), %	PPV	NPV
**7.00**	**50.0**	**66.7**	**12.5**	**93.3**
6.00	50.0	76.2	16.7	94.1
5.00	50.0	90.5	33.3	95.0

The area under the ROC curve comprised 0.607 ± 0.200 with 95% CI: 0.216 - 0.998. The resulting model was not statistically significant ($p = 0.621$).

The cut-off value of NAEM volume which corresponds to the highest Youden's J statistic is 5.000 ml. If NAEM volume was less than this value, +coils was predicted. The sensitivity and specificity of the method were 50.0% and 90.5%, respectively.

Analysis of NAEM volume was performed conditioning on Embolisation by other agents.

Table S64 – Analysis of NAEM volume conditioning on Embolisation by other agents

Variable	Categories	NAEM volume (ml)			*p*
		Me	$Q_1 – Q_3$	n	
Embolisation by other agents	adhesive	6.00	5.00 – 7.00	4	0.377
	alcohol	6.00	6.00 – 6.00	1	
	coils	7.00	7.00 – 7.00	1	
	none	8.00	6.00 – 9.00	17	

When comparing of NAEM volume depending on Embolisation by other agents no statistically significant differences were revealed ($p = 0.377$) *(applied method: The Kruskal-Wallis test)*.

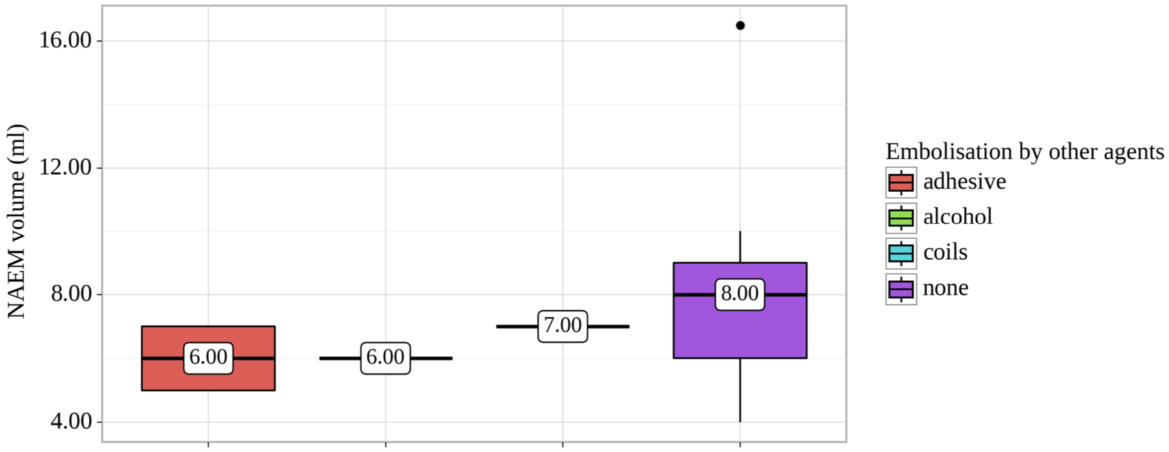

Figure S68 – Analysis of NAEM volume conditioning on Embolisation by other agents

We performed a correlation analysis of the association between NAEM volume and Number of NAEM embolisation steps.

Table S65 – Results of the correlation analysis of the association between NAEM volume and Number of NAEM embolisation steps

Variable	Correlation characteristics		
	ρ	Strength of the association assesed using Chaddock scale	*p*

NAEM volume – Number of NAEM embolisation steps	0.045	None	0.839

There was no association between Number of NAEM embolisation steps and NAEM volume.

Observed dependence of Number of NAEM embolisation steps from NAEM volume is described by a linear regression equation:

$$Y_{\text{Number of NAEM embolisation steps}} = 0.064 \times X_{\text{NAEM volume}} + 0.965$$

With an 1 ml increase of NAEM volume 0.064 change of Number of NAEM embolisation steps should be expected. According to the coefficient of determination R^2 of the resulting model, 3.5% of the observed variance of Number of NAEM embolisation steps were explained..

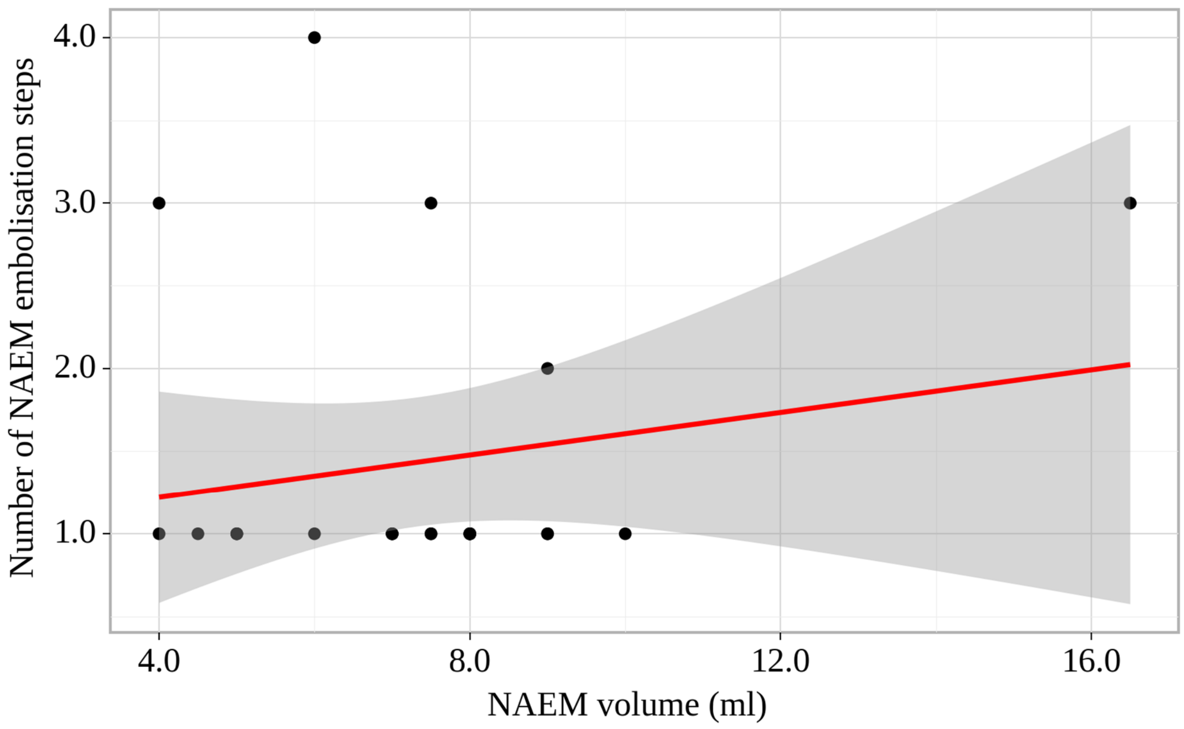

Figure S69 – Regression line characterizing the dependence of Number of NAEM embolisation steps from NAEM volume

We performed analysis of NAEM volume conditioning on Type of Lesion.

Table S66 – Analysis of NAEM volume conditioning on Type of Lesion

Variable	Categories	NAEM volume (ml)			*p*
		Me	$Q_1 – Q_3$	n	
Type of Lesion	Arteriovenous Malformation of Face	7.00	6.00 – 7.00	5	0.172

	Carotid body paraganglioma	8.00	7.75 – 8.50	7	
	Jugular paraganglioma	7.50	4.75 – 8.50	11	

When comparing of NAEM volume depending on Type of Lesion no statistically significant differences were revealed ($p = 0.172$) *(applied method: The Kruskal-Wallis test)*.

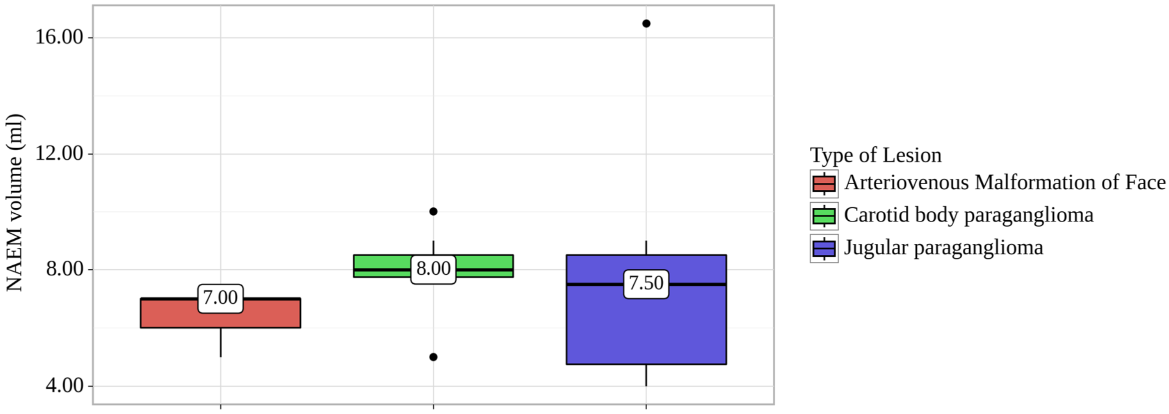

Figure S70 – Analysis of NAEM volume conditioning on Type of Lesion

We performed analysis of NAEM volume conditioning on Radicality embolisation.

Table S67 – Analysis of NAEM volume conditioning on Radicality embolisation

Variable	Categories	NAEM volume (ml)			*p*
		Me	$Q_1 – Q_3$	n	
Radicality embolisation	total	7.00	6.00 – 8.00	17	0.888
	subtotal	7.75	5.25 – 8.75	6	

When comparing of NAEM volume depending on Radicality embolisation there were no statistically significant differences ($p = 0.888$) *(applied method: Mann-Whitney U-test)*.

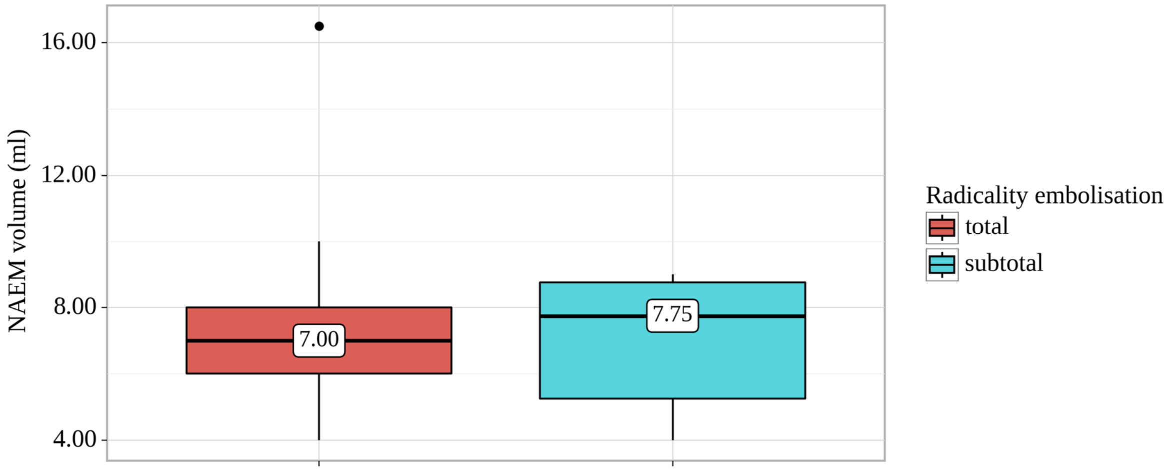

Figure S71 – Analysis of NAEM volume conditioning on Radicality embolisation

When evaluating the dependence of the probability of subtotal on the NAEM volume using the ROC analysis, the following curve was obtained.

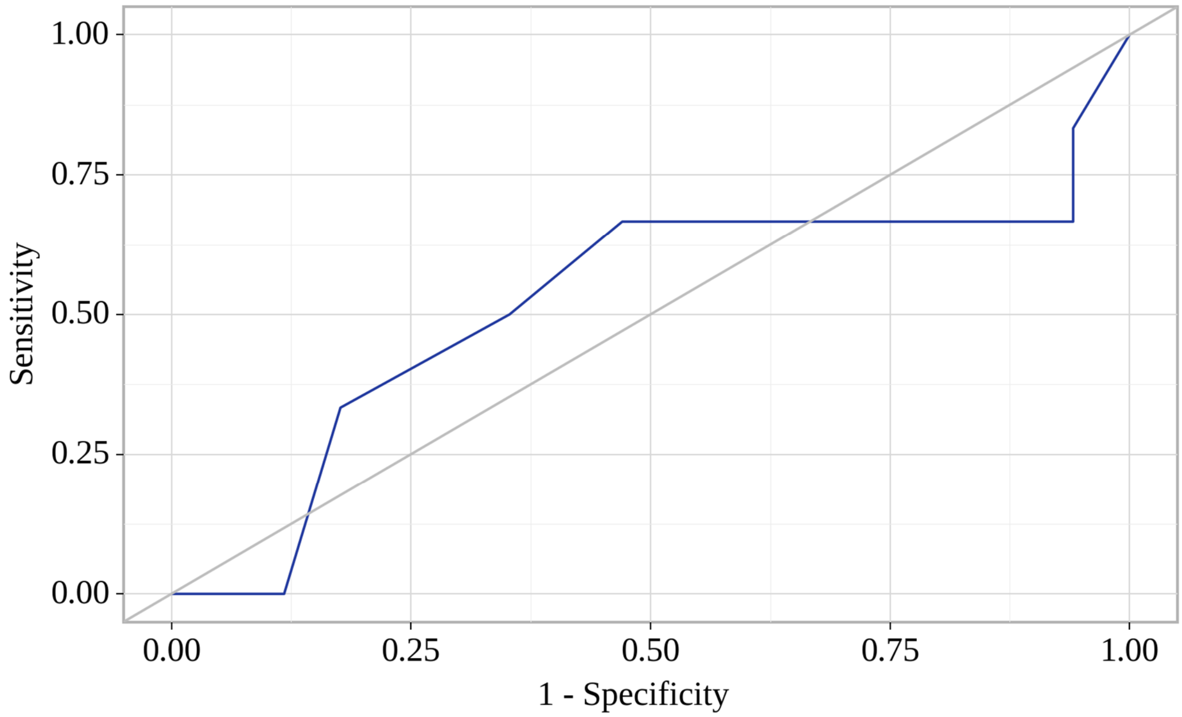

Figure S72 – ROC-curve characterizing the dependence of the probability Radicality embolisation on NAEM volume

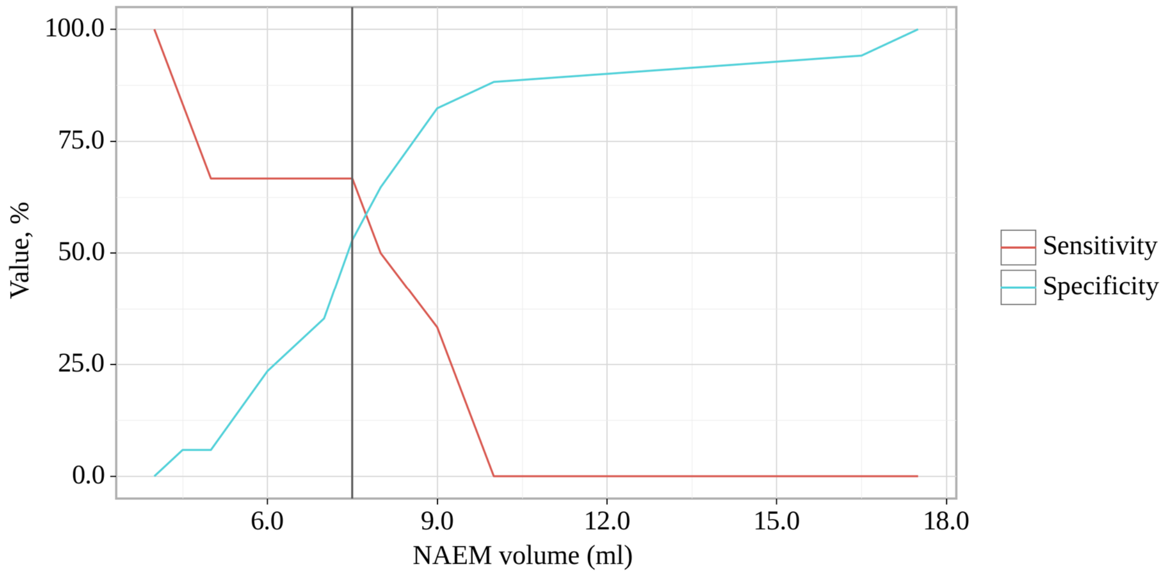

Figure S73 - Analysis of the sensitivity and specificity of Radicality embolisation depending on NAEM volume

Table S68 – Threshold NAEM volume

Threshold	Sensitivity (Se), %	Specificity (Sp), %	PPV	NPV
8.00	50.0	64.7	33.3	78.6
**7.50**	**66.7**	**52.9**	**33.3**	**81.8**

The area under the ROC curve comprised 0.520 ± 0.141 with 95% CI: 0.244 - 0.795. The resulting model was not statistically significant ($p = 0.888$).

The cut-off value of NAEM volume which corresponds to the highest Youden's J statistic is 7.500 ml. If NAEM volume was greater than or equal to this value, subtotal was predicted. The sensitivity and specificity of the method were 66.7% and 52.9%, respectively.

We performed analysis of NAEM volume conditioning on Type of catheter.

Table S69 – Analysis of NAEM volume conditioning on Type of catheter

Variable	Categories	NAEM volume (ml)			*p*
		Me	$Q_1 – Q_3$	n	
Type of catheter	Scepter C, XC	7.50	6.00 – 8.00	21	0.032*
	Headway	4.25	4.12 – 4.38	2	

* – differences are statistically significant ($p < 0.05$)

According to the data obtained when comparing of NAEM volume statistically significant differences were revealed depending on Type of catheter ($p = 0.032$) *(applied method: Mann-Whitney U-test)*.

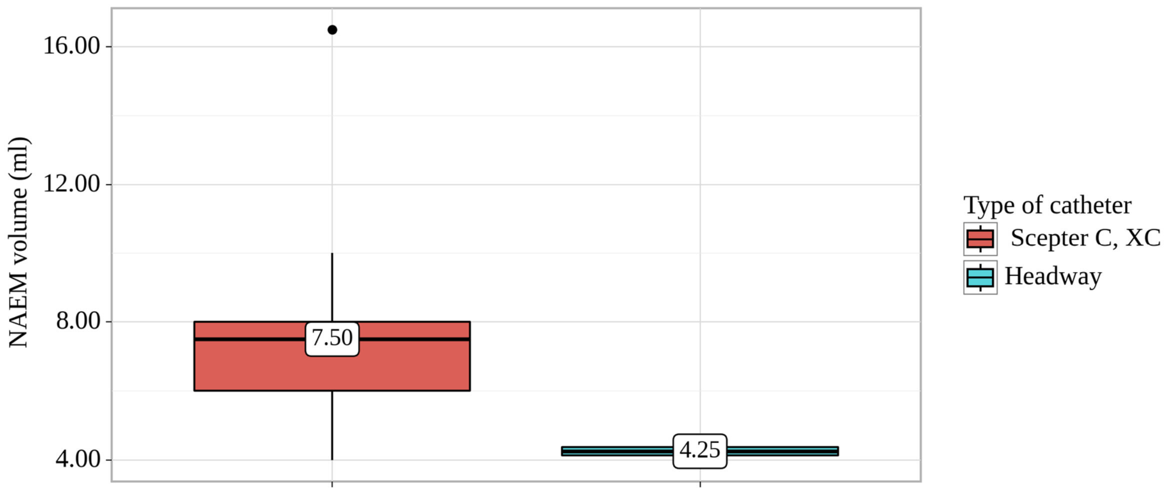

Figure S74 – Analysis of NAEM volume conditioning on Type of catheter

When evaluating the dependence of the probability of Headway on the NAEM volume using the ROC analysis, the following curve was obtained.

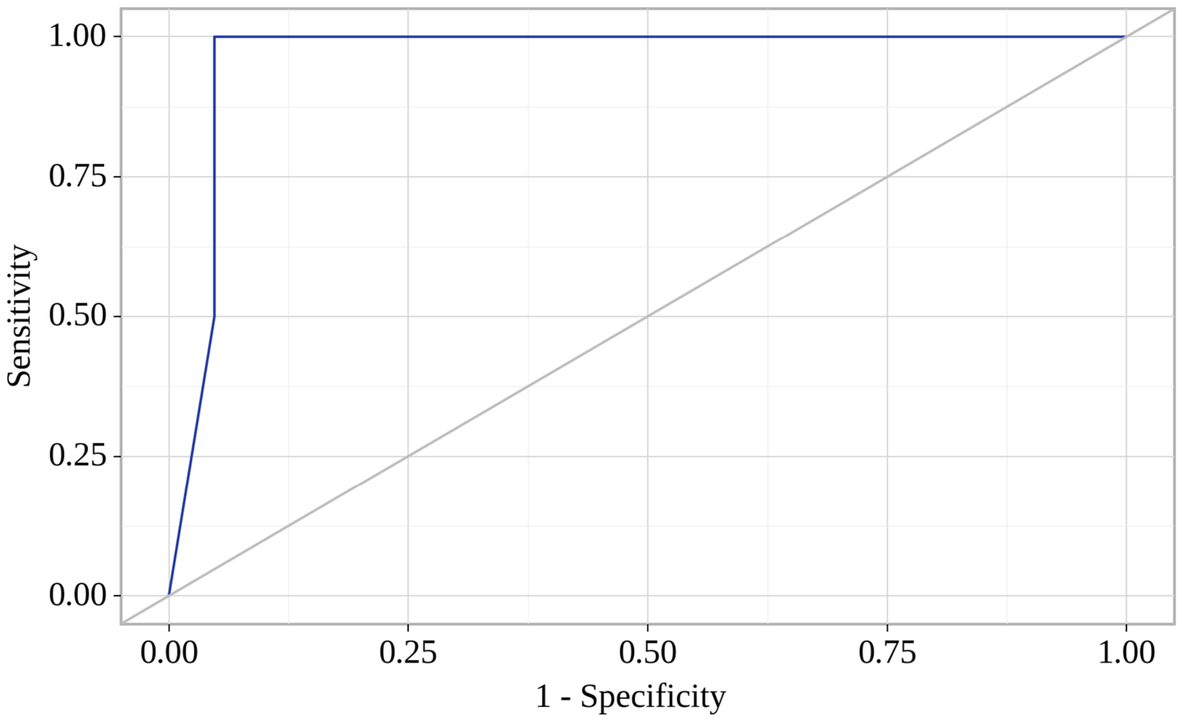

Figure S75 – ROC-curve characterizing the dependence of the probability Type of catheter on NAEM volume

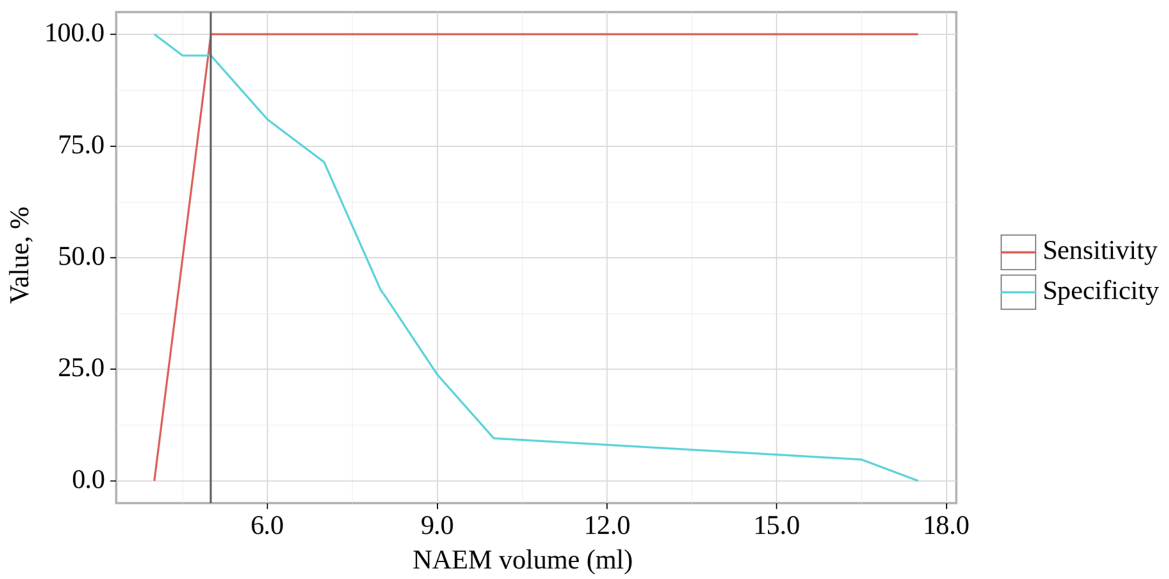

Figure S76 - Analysis of the sensitivity and specificity of Type of catheter depending on NAEM volume

Table S70 – Threshold NAEM volume

Threshold	Sensitivity (Se), %	Specificity (Sp), %	PPV	NPV
7.00	100.0	71.4	25.0	100.0
6.00	100.0	81.0	33.3	100.0
**5.00**	**100.0**	**95.2**	**66.7**	**100.0**
4.50	50.0	95.2	50.0	95.2

The area under the ROC curve comprised 0.964 ± 0.042 with 95% CI: 0.881 - 1.000. The resulting model was statistically significant (p = 0.032).

The cut-off value of NAEM volume which corresponds to the highest Youden's J statistic is 5.000 ml. If NAEM volume was less than this value, Headway was predicted. The sensitivity and specificity of the method were 100.0% and 95.2%, respectively.

Analysis of Age was performed conditioning on Complications.

Table S71 – Analysis of Age conditioning on Complications

Variable	Categories	Age (year)			p
		Me	$Q_1 – Q_3$	n	
Complications	none	52	46 – 66	22	0.327
	cerebral ischaemia	68	68 – 68	1	

When comparing of Age depending on Complications there were no statistically significant differences (p = 0.327) *(applied method: Mann-Whitney U-test)*.

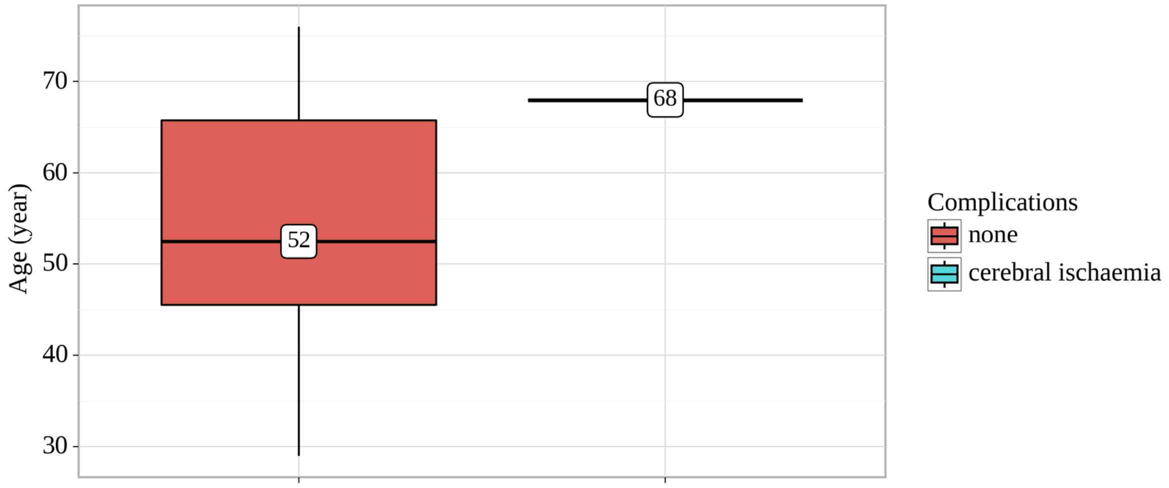

Figure S77 – Analysis of Age conditioning on Complications

Analysis of Type of Lesion was performed conditioning on Complications.

Table S72 – Analysis of Type of Lesion conditioning on Complications

Variable	Categories	Complications		*p*
		none	cerebral ischaemia	
Type of Lesion	Arteriovenous Malformation of Face	5 (22.7)	0 (0.0)	0.565
	Carotid body paraganglioma	7 (31.8)	0 (0.0)	
	Jugular paraganglioma	10 (45.5)	1 (100.0)	

When comparing of Type of Lesion depending on Complications no statistically significant differences were revealed (*p* = 0.565) *(applied method: Pearson's chi-square test)*.

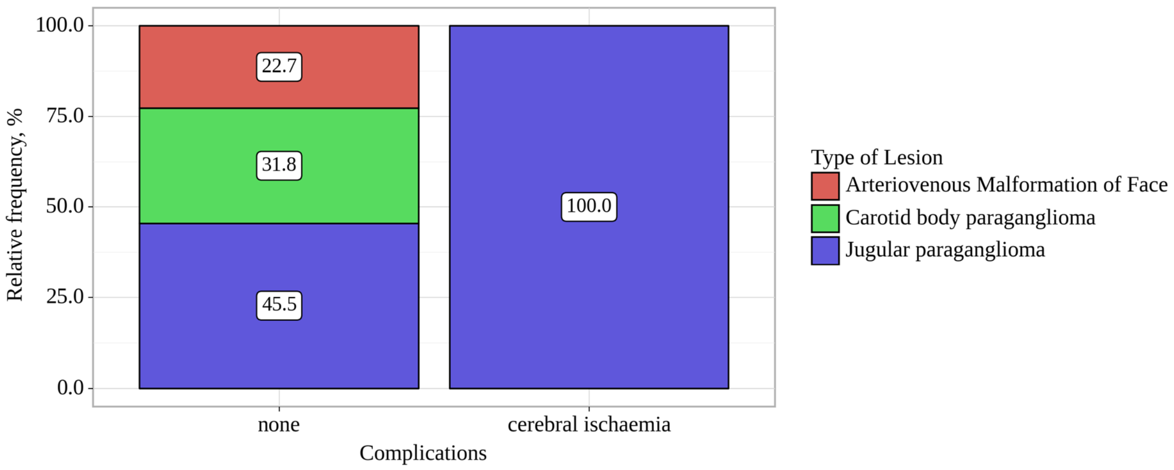

Figure S78 – Analysis of Type of Lesion conditioning on Complications

We performed analysis of Number of NAEM embolisation steps conditioning on Complications.

Table S73 – Analysis of Number of NAEM embolisation steps conditioning on Complications

Variable	Categories	Number of NAEM embolisation steps			*p*
		Me	$Q_1 – Q_3$	n	
Complications	none	1	1 – 1	22	0.601
	cerebral ischaemia	1	1 – 1	1	

When comparing of Number of NAEM embolisation steps depending on Complications there were no statistically significant differences (*p* = 0.601) *(applied method: Mann-Whitney U-test)*.

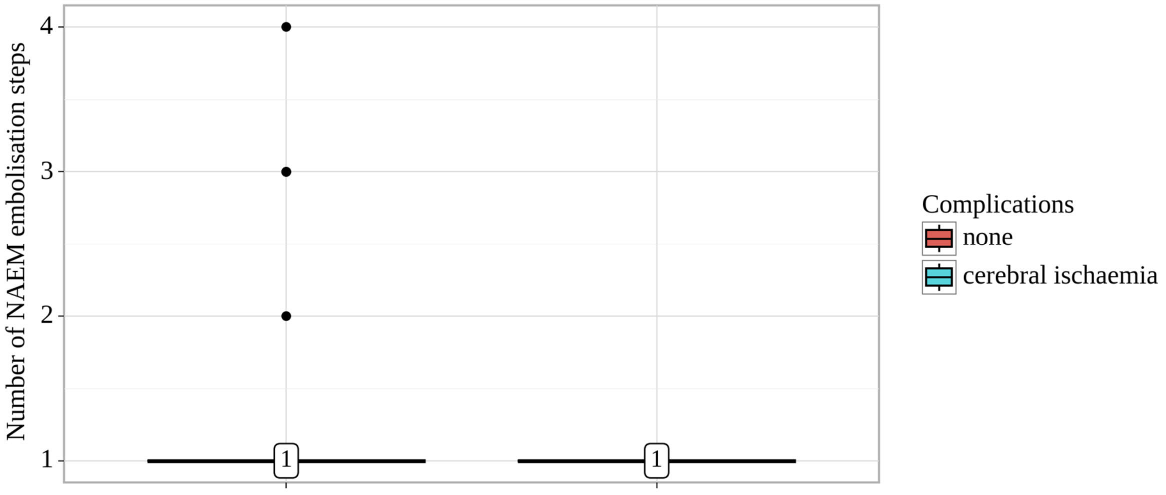

Figure S79 – Analysis of Number of NAEM embolisation steps conditioning on Complications

We performed analysis of Time of embolisation conditioning on Complications.

Table S74 – Analysis of Time of embolisation conditioning on Complications

Variable	Categories	Time of embolisation (min)			*p*
		Me	$Q_1 – Q_3$	n	
Complications	none	90	75 – 110	22	0.255
	cerebral ischaemia	120	120 – 120	1	

When comparing of Time of embolisation depending on Complications no statistically significant differences were revealed (*p* = 0.255) *(applied method: Mann-Whitney U-test)*.

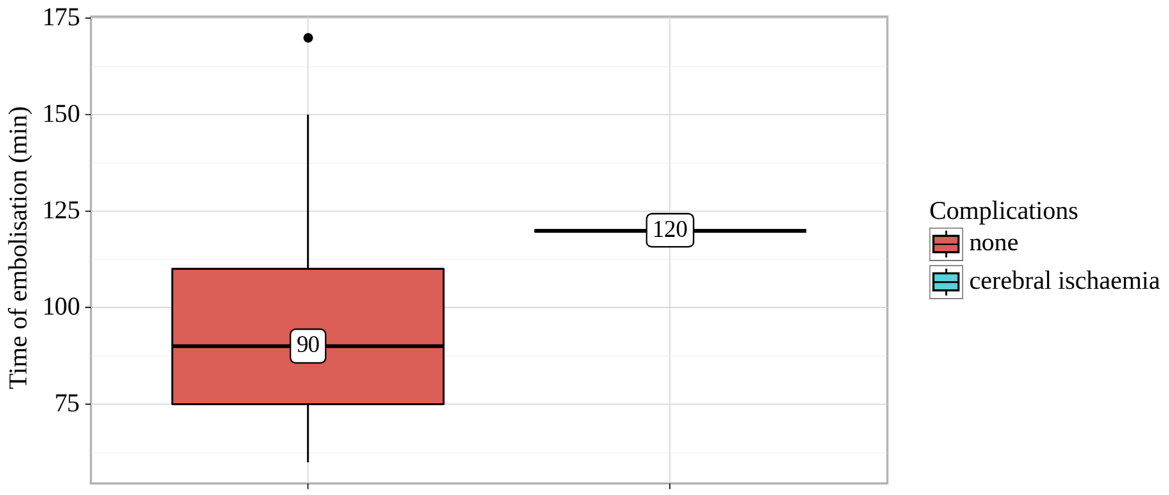

Figure S80 – Analysis of Time of embolisation conditioning on Complications

Analysis of Type of NAEM was performed conditioning on Complications.

Table S75 – Analysis of Type of NAEM conditioning on Complications

Variable	Categories	Complications		*p*
		none	cerebral ischaemia	
Type of NAEM	Squid	11 (50.0)	1 (100.0)	0.811
	Onyx+Squid	2 (9.1)	0 (0.0)	
	Onyx	8 (36.4)	0 (0.0)	
	Phill	1 (4.5)	0 (0.0)	

When comparing of Type of NAEM depending on Complications there were no statistically significant differences ($p = 0.811$) *(applied method: Pearson's chi-square test)*.

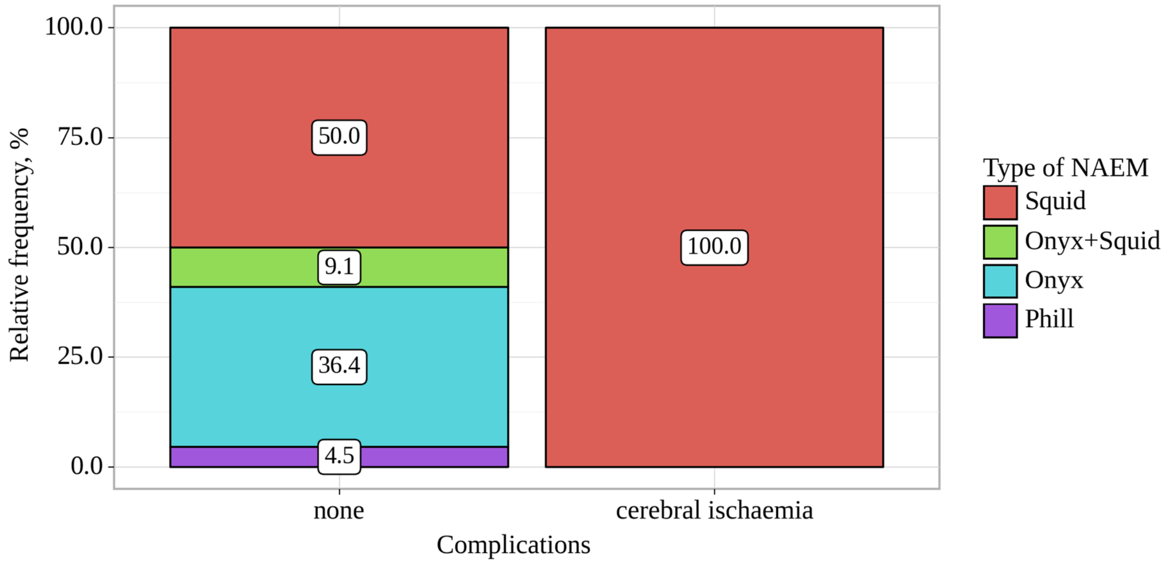

Figure S81 – Analysis of Type of NAEM conditioning on Complications

Analysis of Coils for NAEM was performed conditioning on Complications.

Table S76 – Analysis of Coils for NAEM conditioning on Complications

Variable	Categories	Complications		*p*
		none	cerebral ischaemia	
Coils for NAEM	none	20 (90.9)	1 (100.0)	1.000
	+coils	2 (9.1)	0 (0.0)	

When comparing of Coils for NAEM depending on Complications there were no statistically significant differences ($p = 1.000$) *(applied method: Fisher's exact test)*.

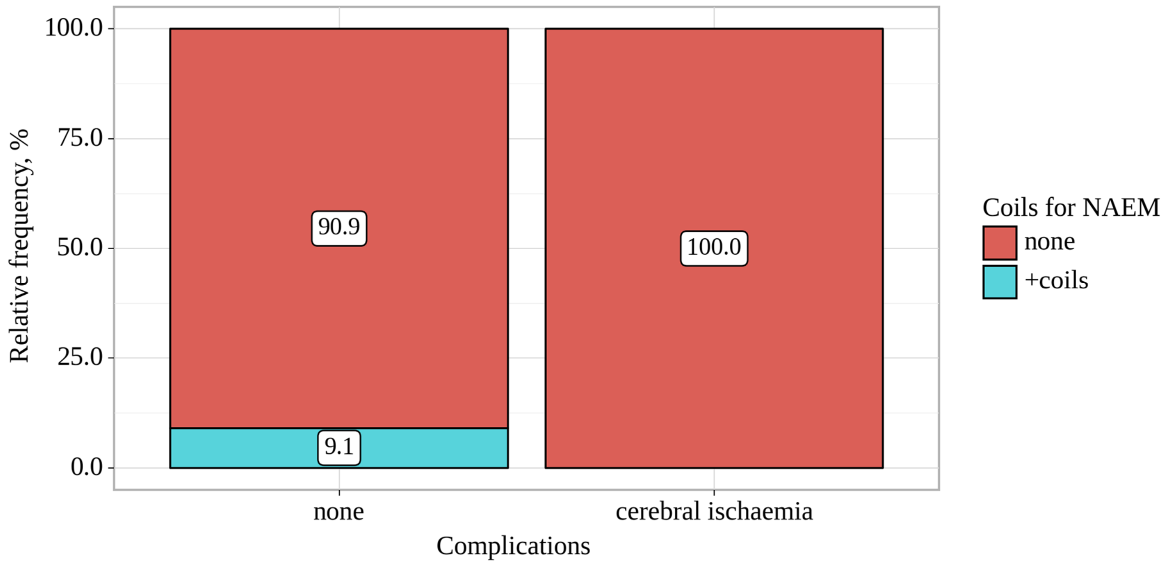

Figure S82 – Analysis of Coils for NAEM conditioning on Complications

Odds of +coils were 2.733 times greater in cerebral ischaemia group comparing with none, the relative difference in odds was not statistically significant (95% CI: 0.086 – 86.919).

We performed analysis of Radicality embolisation conditioning on Complications.

Table S77 – Analysis of Radicality embolisation conditioning on Complications

Variable	Categories	Complications		*p*
		none	cerebral ischaemia	
Radicality embolisation	total	17 (77.3)	0 (0.0)	0.261
	subtotal	5 (22.7)	1 (100.0)	

When comparing of Radicality embolisation depending on Complications there were no statistically significant differences ($p = 0.261$) *(applied method: Fisher's exact test)*.

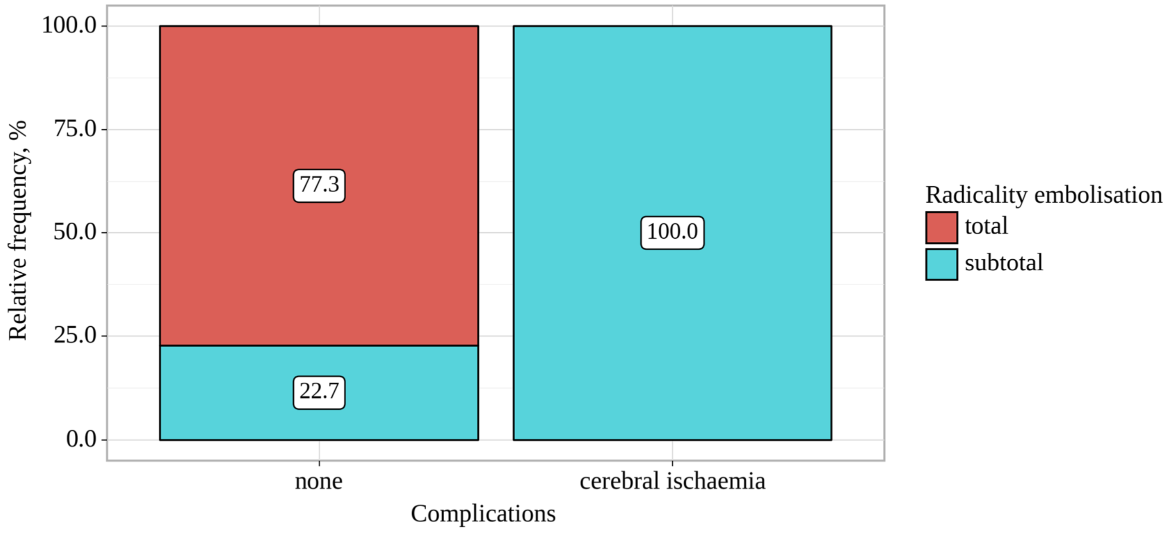

Figure S83 – Analysis of Radicality embolisation conditioning on Complications

Odds of subtotal were 9.545 times greater in cerebral ischaemia group comparing with none, the relative difference in odds was not statistically significant (95% CI: 0.338 – 269.616).

Analysis of Type of catheter was performed conditioning on Complications.

Table S78 – Analysis of Type of catheter conditioning on Complications

Variable	Categories	Complications		*p*
		none	cerebral ischaemia	
Type of catheter	Scepter C, XC	20 (90.9)	1 (100.0)	1.000
	Headway	2 (9.1)	0 (0.0)	

When comparing of Type of catheter depending on Complications there were no statistically significant differences (p = 1.000) *(applied method: Fisher's exact test)*.

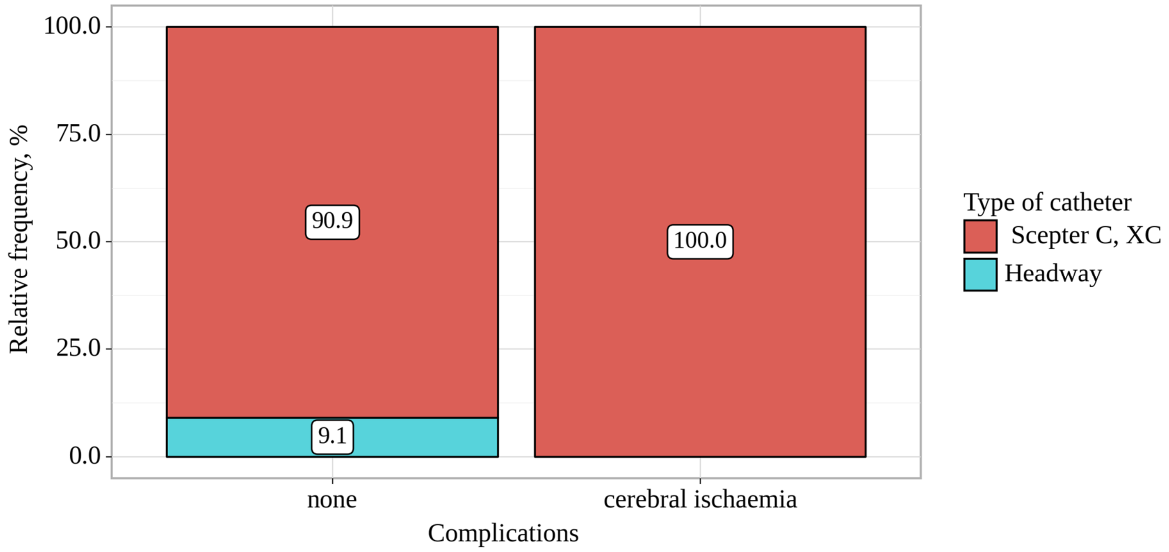

Figure S84 – Analysis of Type of catheter conditioning on Complications

Odds of Headway were 2.733 times greater in cerebral ischaemia group comparing with none, the relative difference in odds was not statistically significant (95% CI: 0.086 – 86.919).

Analysis of NAEM volume was performed conditioning on Complications.

Table S79 – Analysis of NAEM volume conditioning on Complications

Variable	Categories	NAEM volume (ml)			*p*
		Me	$Q_1 – Q_3$	n	
Complications	none	7.25	5.25 – 8.00	22	0.879
	cerebral ischaemia	7.50	7.50 – 7.50	1	

When comparing of NAEM volume depending on Complications there were no statistically significant differences (p = 0.879) *(applied method: Mann-Whitney U-test)*.

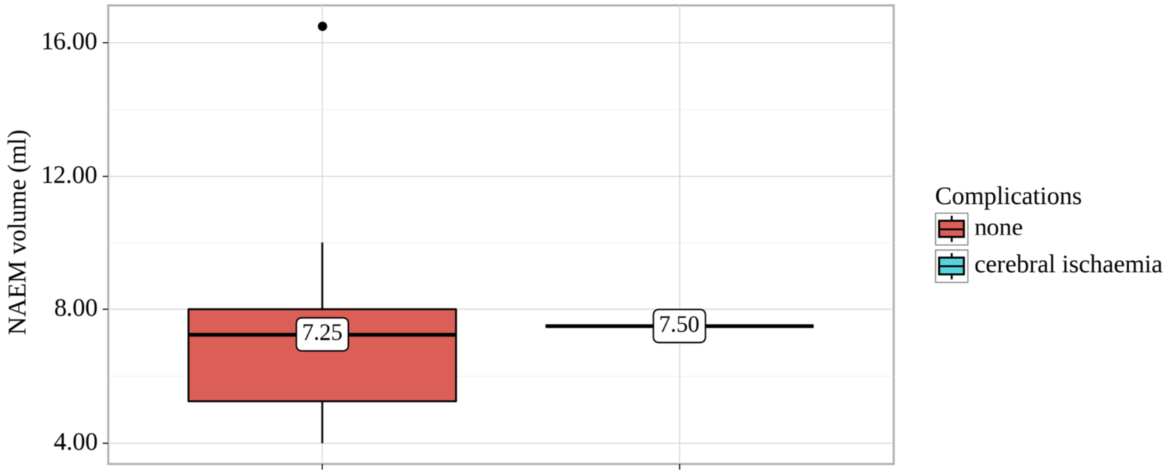

Figure S85 – Analysis of NAEM volume conditioning on Complications

Correlation analysis of the association between mRS at discharge and Age was performed.

Table S80 – Results of the correlation analysis of the association between mRS at discharge and Age

Variable	Correlation characteristics		
	ρ	Strength of the association assesed using Chaddock scale	*p*
mRS at discharge – Age	0.022	None	0.920

There was no association between Age and mRS at discharge.

Observed dependence of Age from mRS at discharge is described by a linear regression equation:

$$Y_{Age} = 0.005 \times X_{mRS\ at\ discharge} + 54.78$$

With an 1 increase of mRS at discharge 0.005 year change of Age should be expected. According to the coefficient of determination R^2 of the resulting model, 0.0% of the observed variance of Age were explained..

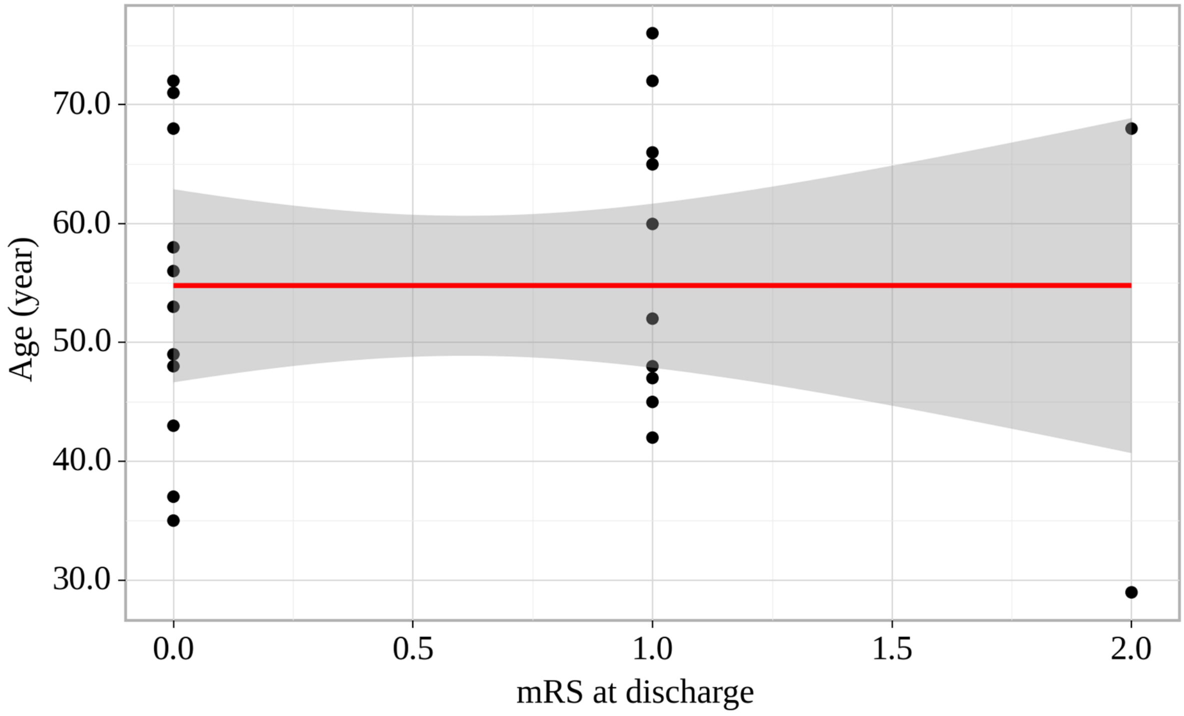

Figure S86 – Regression line characterizing the dependence of Age from mRS at discharge

We performed analysis of mRS at discharge conditioning on Type of Lesion.

Table S81 – Analysis of mRS at discharge conditioning on Type of Lesion

Variable	Categories	mRS at discharge			*p*
		Me	$Q_1 - Q_3$	n	

Type of Lesion	Arteriovenous Malformation of Face	0	0 – 1	5	0.001* $p_{\text{Jugular paraganglioma – Carotid body paraganglioma}} < 0.001$
	Carotid body paraganglioma	0	0 – 0	7	
	Jugular paraganglioma	1	1 – 1	11	

* – differences are statistically significant ($p < 0.05$)

According to the data obtained when comparing of mRS at discharge statistically significant differences were revealed depending on Type of Lesion ($p = 0.001$) *(applied method: The Kruskal-Wallis test)*.

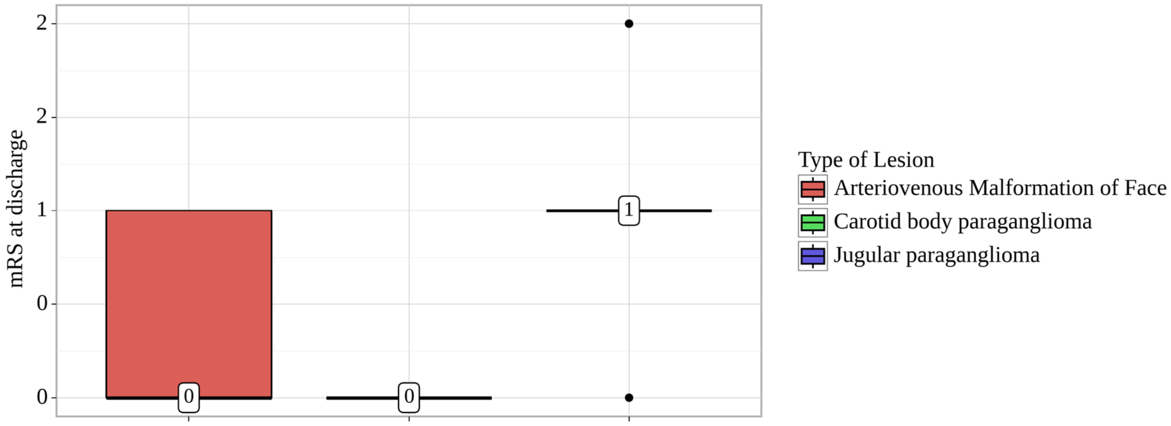

Figure S87 – Analysis of mRS at discharge conditioning on Type of Lesion

Correlation analysis of the association between mRS at discharge and Number of NAEM embolisation steps was performed.

Table S82 – Results of the correlation analysis of the association between mRS at discharge and Number of NAEM embolisation steps

Variable	Correlation characteristics		
	ρ	Strength of the association assesed using Chaddock scale	*p*
mRS at discharge – Number of NAEM embolisation steps	0.348	Moderate	0.104

A moderate correlation positive association between Number of NAEM embolisation steps and mRS at discharge was estimated.

Observed dependence of Number of NAEM embolisation steps from mRS at discharge is described by a linear regression equation:

$$Y_{\text{Number of NAEM embolisation steps}} = 0.518 \times X_{\text{mRS at discharge}} + 1.119$$

With an 1 increase of mRS at discharge 0.518 change of Number of NAEM embolisation steps should be expected. According to the coefficient of determination R^2 of the resulting model, 14.4% of the observed variance of Number of NAEM embolisation steps were explained..

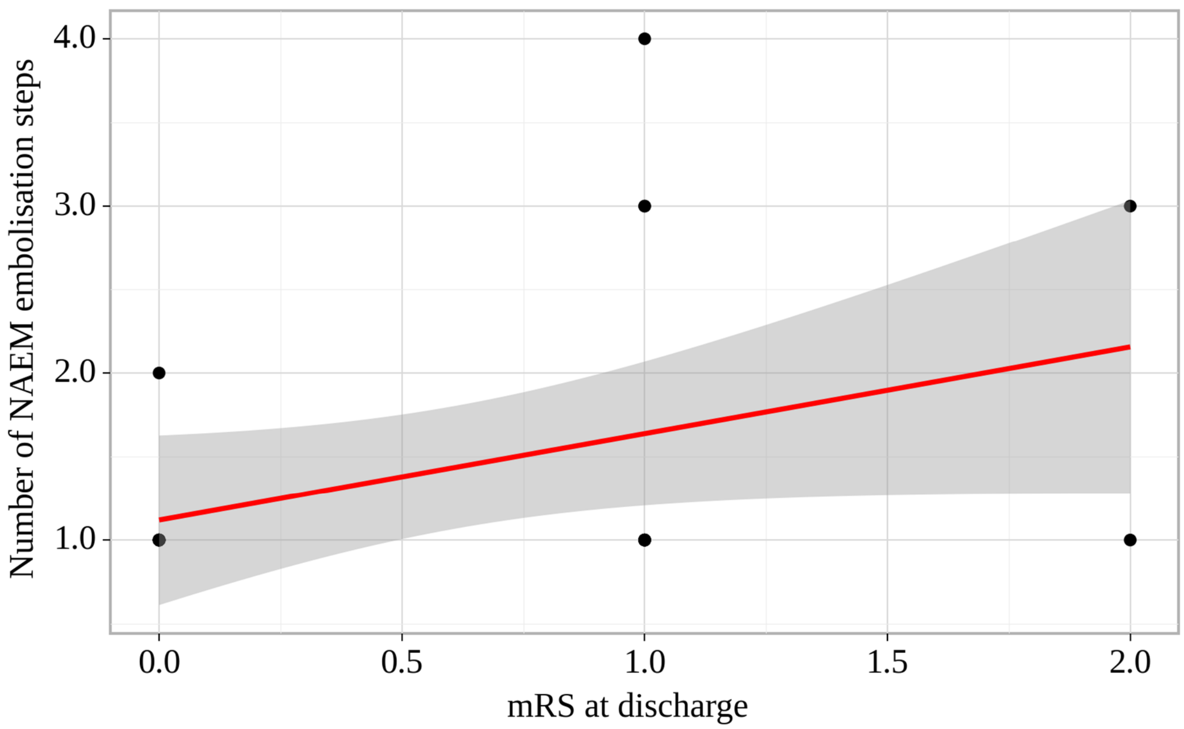

Figure S88 – Regression line characterizing the dependence of Number of NAEM embolisation steps from mRS at discharge

Analysis of mRS at discharge was performed conditioning on Open Surgical Interventions.

Table S83 – Analysis of mRS at discharge conditioning on Open Surgical Interventions

Variable	Categories	mRS at discharge			*p*
		Me	$Q_1 – Q_3$	n	
Open Surgical Interventions	after embolisation	1	1 – 1	4	0.566
	before embolisation	1	1 – 1	1	
	none	0	0 – 1	18	

When comparing of mRS at discharge depending on Open Surgical Interventions there were no statistically significant differences ($p = 0.566$) *(applied method: The Kruskal-Wallis test)*.

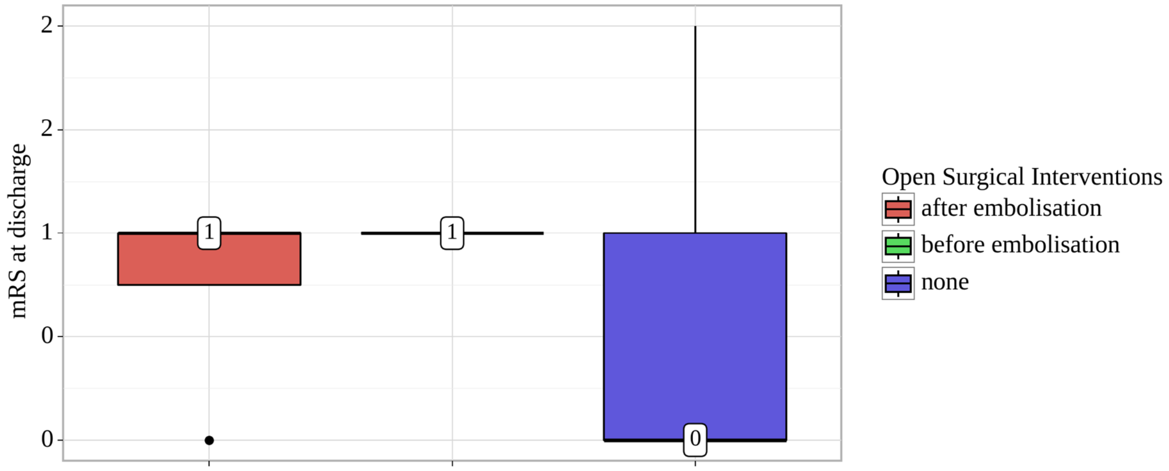

Figure S89 – Analysis of mRS at discharge conditioning on Open Surgical Interventions

We performed a correlation analysis of the association between mRS at discharge and mRS before embolisation.

Table S84 – Results of the correlation analysis of the association between mRS at discharge and mRS before embolisation

Variable	Correlation characteristics		
	ϱ	Strength of the association assesed using Chaddock scale	*p*
mRS at discharge – mRS before embolisation	0.738	Strong	< 0.001*

* – differences are statistically significant ($p < 0.05$)

A strong correlation positive association between mRS before embolisation and mRS at discharge was estimated.

Observed dependence of mRS before embolisation from mRS at discharge is described by a linear regression equation:

$$Y_{\text{mRS before embolisation}} = 0.959 \times X_{\text{mRS at discharge}} - 0.018$$

With an 1 increase of mRS at discharge 0.959 change of mRS before embolisation should be expected. According to the coefficient of determination R^2 of the resulting model, 63.8% of the observed variance of mRS before embolisation were explained..

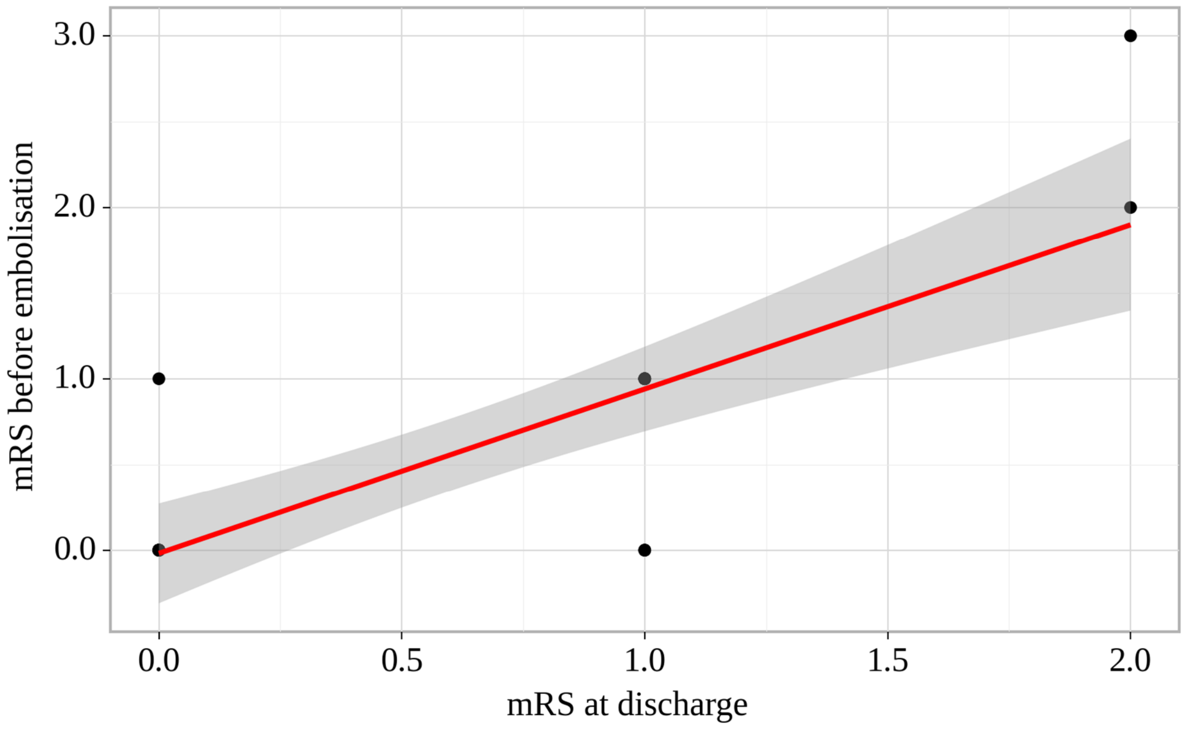

Figure S90 – Regression line characterizing the dependence of mRS before embolisation from mRS at discharge

We performed a correlation analysis of the association between mRS at discharge and Time of embolisation.

Table S85 – Results of the correlation analysis of the association between mRS at discharge and Time of embolisation

Variable	Correlation characteristics		
	ρ	Strength of the association assesed using Chaddock scale	*p*
mRS at discharge – Time of embolisation	0.208	Weak	0.340

A weak correlation positive association between Time of embolisation and mRS at discharge was estimated.

Observed dependence of Time of embolisation from mRS at discharge is described by a linear regression equation:

$$Y_{\text{Time of embolisation}} = 7.982 \times X_{\text{mRS at discharge}} + 91.881$$

With an 1 increase of mRS at discharge 7.982 min change of Time of embolisation should be expected. According to the coefficient of determination R^2 of the resulting model, 3.5% of the observed variance of Time of embolisation were explained..

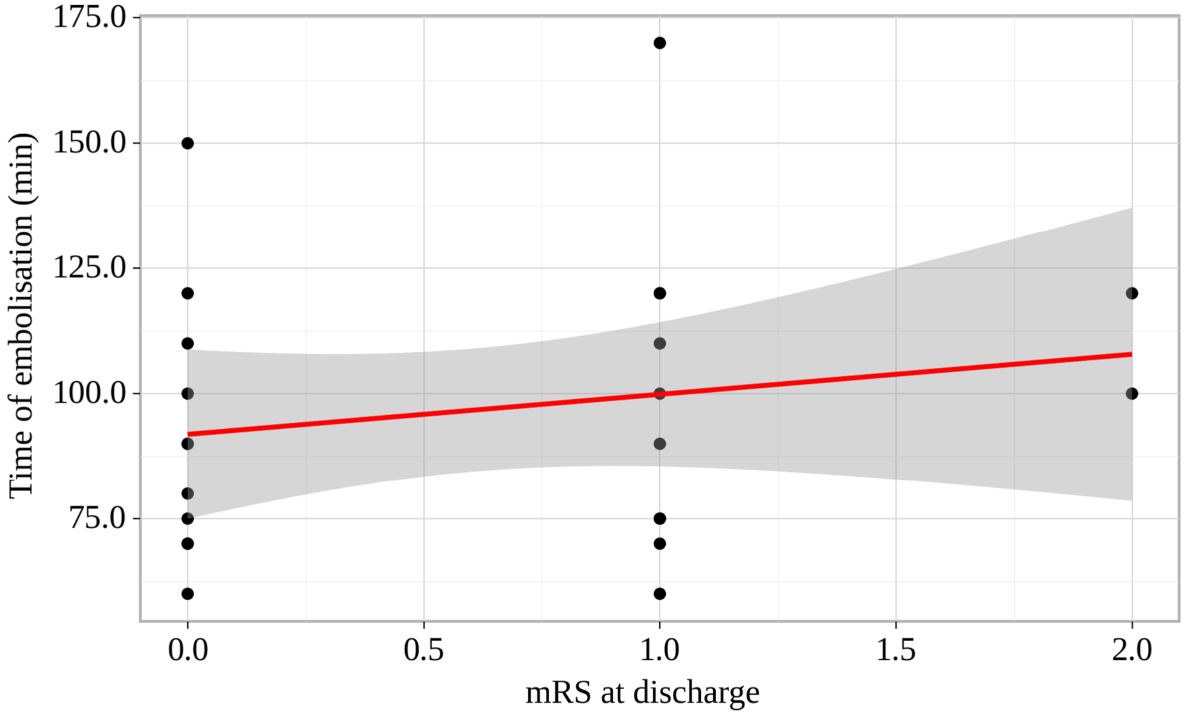

Figure S91 – Regression line characterizing the dependence of Time of embolisation from mRS at discharge

We performed analysis of mRS at discharge conditioning on Type of NAEM.

Table S86 – Analysis of mRS at discharge conditioning on Type of NAEM

Variable	Categories	mRS at discharge			*p*
		Me	$Q_1 – Q_3$	n	
Type of NAEM	Squid	0	0 – 1	12	0.212
	Onyx+Squid	2	1 – 2	2	
	Onyx	0	0 – 1	8	
	Phill	1	1 – 1	1	

When comparing of mRS at discharge depending on Type of NAEM there were no statistically significant differences ($p = 0.212$) *(applied method: The Kruskal-Wallis test)*.

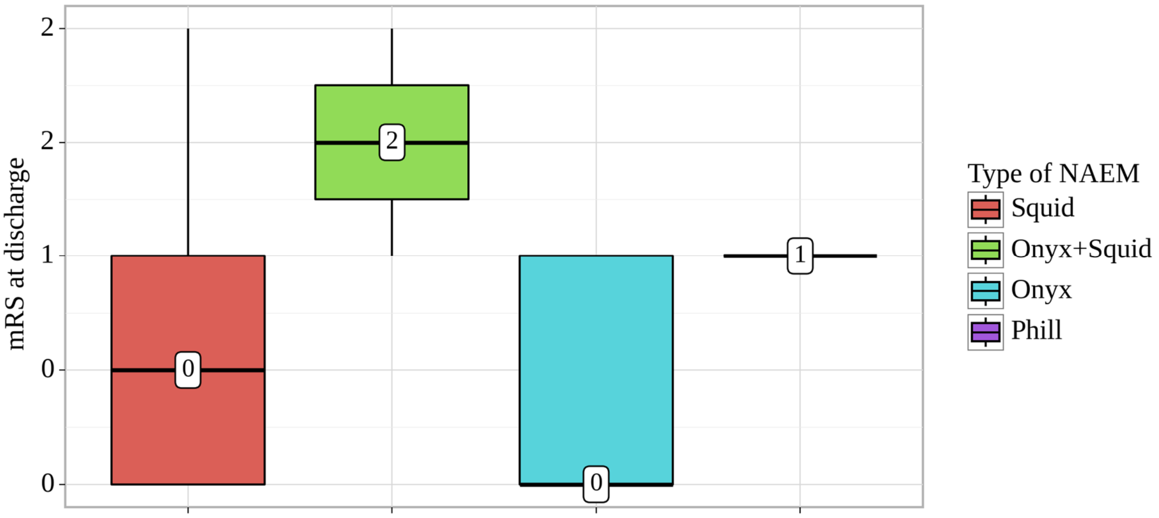

Figure S92 – Analysis of mRS at discharge conditioning on Type of NAEM

Analysis of mRS at discharge was performed conditioning on Radicality embolisation.

Table S87 – Analysis of mRS at discharge conditioning on Radicality embolisation

Variable	Categories	mRS at discharge			p
		Me	$Q_1 – Q_3$	n	
Radicality embolisation	total	0	0 – 1	17	0.080
	subtotal	1	1 – 1	6	

When comparing of mRS at discharge depending on Radicality embolisation there were no statistically significant differences ($p = 0.080$) *(applied method: Mann-Whitney U-test)*.

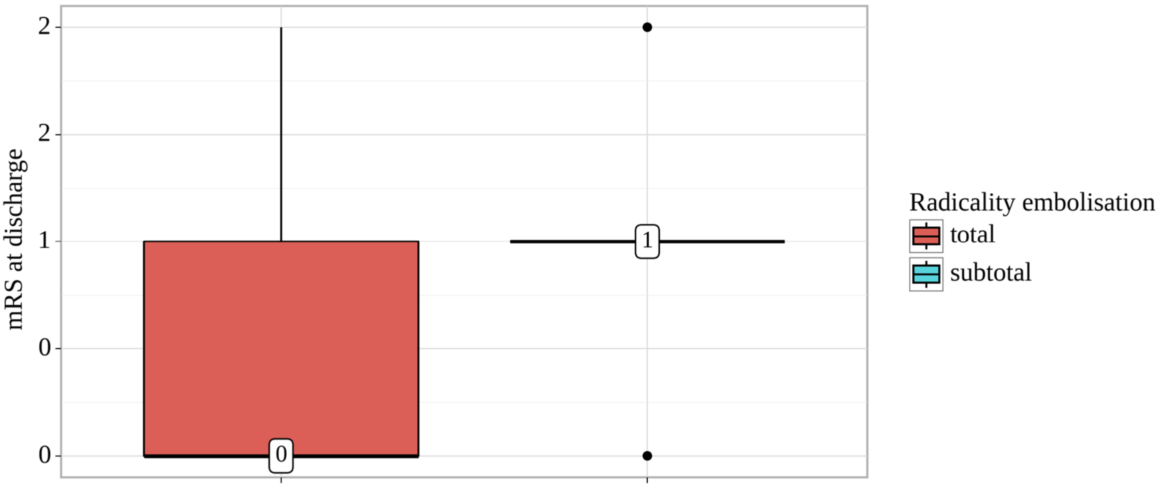

Figure S93 – Analysis of mRS at discharge conditioning on Radicality embolisation

When evaluating the dependence of the probability of subtotal on the mRS at discharge using the ROC analysis, the following curve was obtained.

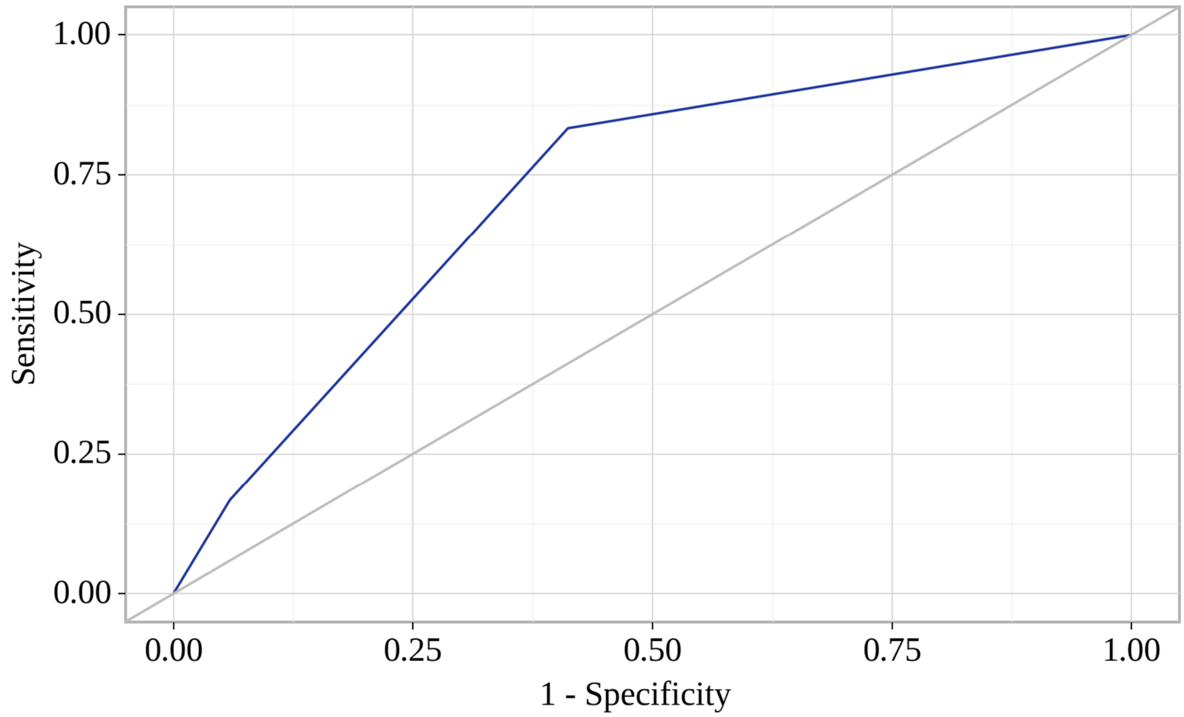

Figure S94 – ROC-curve characterizing the dependence of the probability Radicality embolisation on mRS at discharge

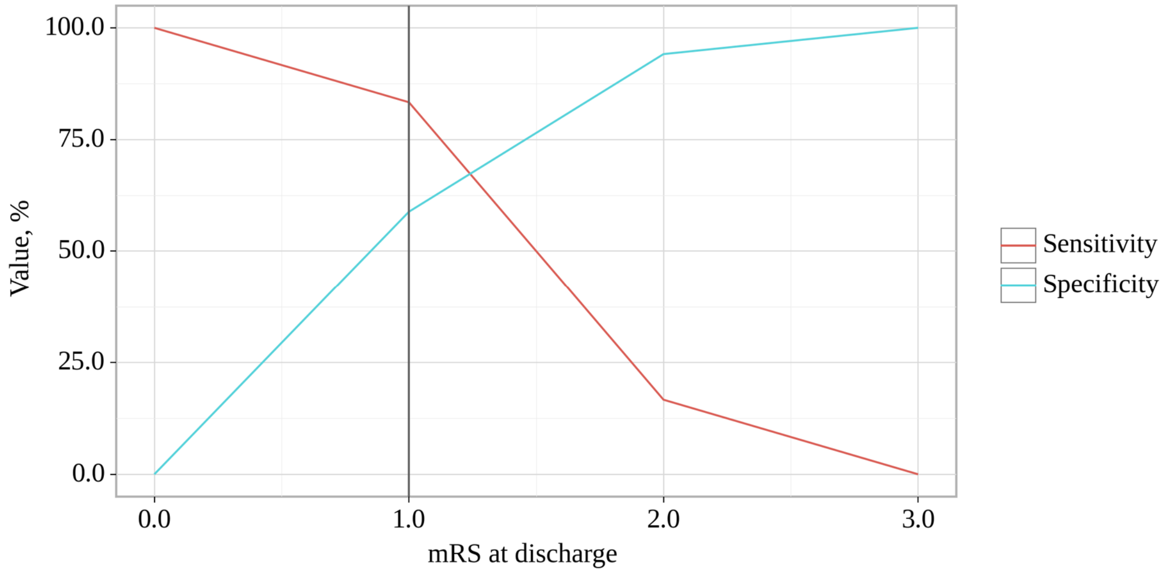

Figure S95 - Analysis of the sensitivity and specificity of Radicality embolisation depending on mRS at discharge

Table S88 – Threshold mRS at discharge

Threshold	Sensitivity (Se), %	Specificity (Sp), %	PPV	NPV
**1**	**83.3**	**58.8**	**41.7**	**90.9**

The area under the ROC curve comprised 0.721 ± 0.132 with 95% CI: 0.462 - 0.979. The resulting model was not statistically significant (p = 0.080).

The cut-off value of mRS at discharge which corresponds to the highest Youden's J statistic is 1.000. If mRS at discharge was greater than or equal to this value, subtotal was predicted. The sensitivity and specificity of the method were 83.3% and 58.8%, respectively.

We performed a correlation analysis of the association between mRS at discharge and NAEM volume.

Table S89 – Results of the correlation analysis of the association between mRS at discharge and NAEM volume

Variable	Correlation characteristics		
	ρ	Strength of the association assesed using Chaddock scale	*p*
mRS at discharge – NAEM volume	-0.013	None	0.952

There was no association between NAEM volume and mRS at discharge.

Observed dependence of NAEM volume from mRS at discharge is described by a linear regression equation:

$$Y_{\text{NAEM volume}} = 1.048 \times X_{\text{mRS at discharge}} + 6.688$$

With an 1 increase of mRS at discharge 1.048 ml change of NAEM volume should be expected. According to the coefficient of determination R^2 of the resulting model, 6.9% of the observed variance of NAEM volume were explained..

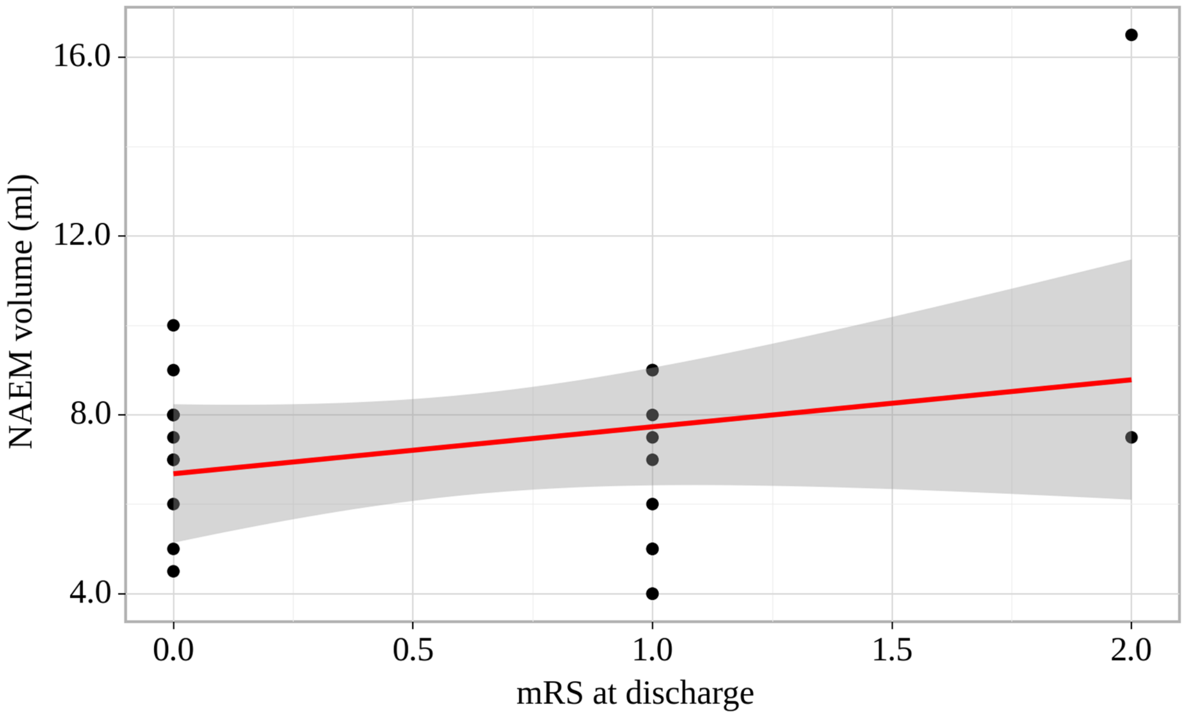

Figure S96 – Regression line characterizing the dependence of NAEM volume from mRS at discharge

We performed analysis of mRS at discharge conditioning on Complications.

Table S90 – Analysis of mRS at discharge conditioning on Complications

Variable	Categories	mRS at discharge			p
		Me	$Q_1 – Q_3$	n	
Complications	none	0	0 – 1	22	0.078
	cerebral ischaemia	2	2 – 2	1	

When comparing of mRS at discharge depending on Complications no statistically significant differences were revealed (p = 0.078) *(applied method: Mann-Whitney U-test)*.

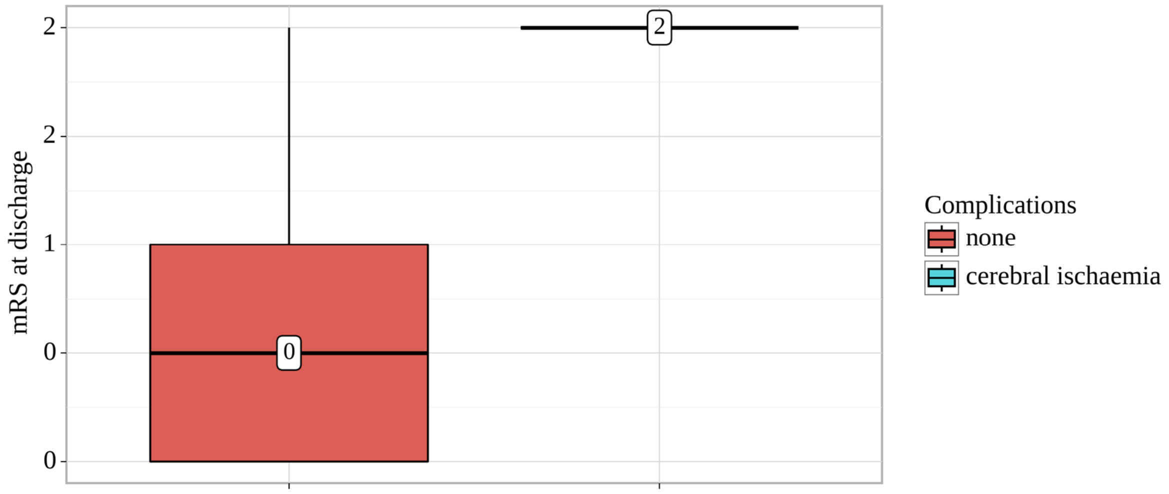

Figure S97 – Analysis of mRS at discharge conditioning on Complications

When evaluating the dependence of the probability of cerebral ischaemia on the mRS at discharge using the ROC analysis, the following curve was obtained.

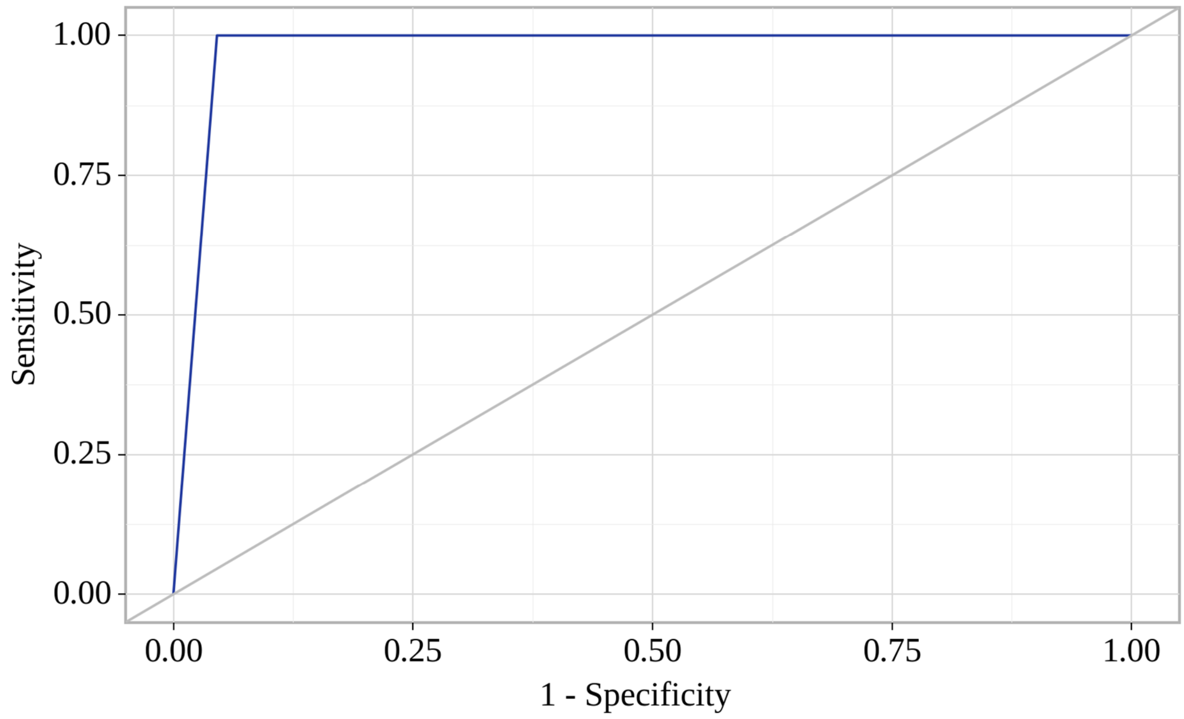

Figure S98 – ROC-curve characterizing the dependence of the probability Complications on mRS at discharge

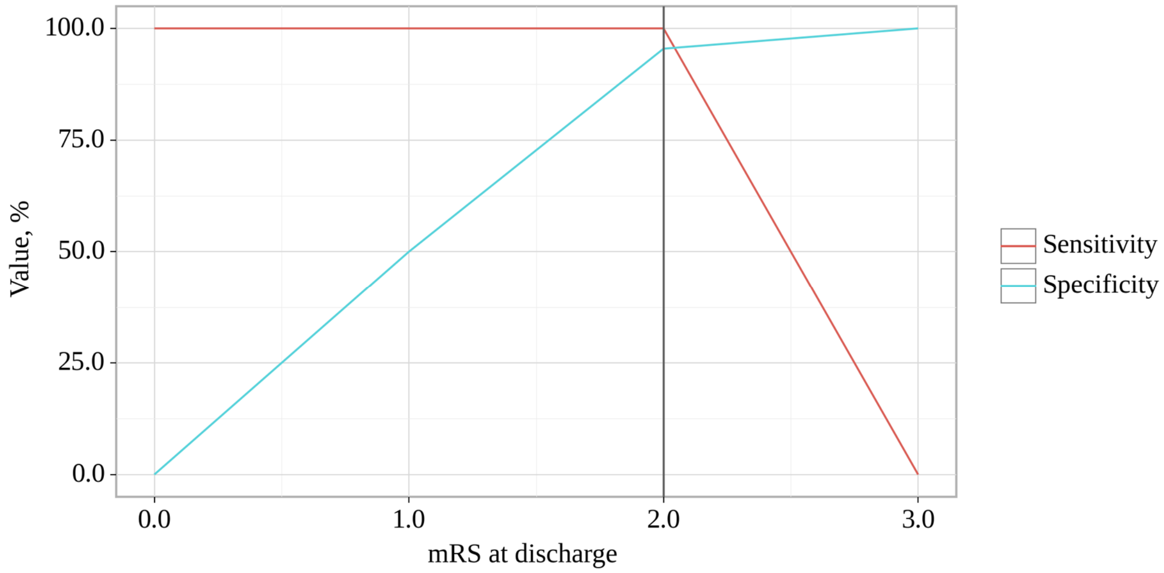

Figure S99 - Analysis of the sensitivity and specificity of Complications depending on mRS at discharge

Table S91 – Threshold mRS at discharge

Threshold	Sensitivity (Se), %	Specificity (Sp), %	PPV	NPV
**2**	**100.0**	**95.5**	**50.0**	**100.0**
1	100.0	50.0	8.3	100.0

The area under the ROC curve comprised 0.977 ± 0.107 with 95% CI: 0.767 - 1.000. The resulting model was not statistically significant (p = 0.078).

The cut-off value of mRS at discharge which corresponds to the highest Youden's J statistic is 2.000. If mRS at discharge was greater than or equal to this value, cerebral ischaemia was predicted. The sensitivity and specificity of the method were 100.0% and 95.5%, respectively.

The selection of predictors of Type of Lesion did not reveal statistically significant associations.

The selection of predictors of Number of treatment stages did not reveal statistically significant associations.

The selection of predictors of Number of NAEM embolisation steps did not reveal statistically significant associations.

The selection of predictors of Open Surgical Interventions did not reveal statistically significant associations.

The selection of predictors of Embolisation by other agents did not reveal statistically significant associations.

The dependence of mRS before embolisation on quantitative variables was estimated using multiple linear regression. The number of observations was 23.

Table S92 – Analysis of mRS before embolisation conditioning on Number of treatment stages, Complications

	B	std error	t	*p*
Intercept	-0.141	0.273	-0.518	0.610
Number of treatment stages	0.328	0.120	2.727	0.013*
Complications: cerebral ischaemia	1.813	0.672	2.699	0.014*

* – differences are statistically significant ($p < 0.05$)

The observed association of mRS before embolisation with Number of treatment stages, Complications is presented by a linear regression equation:

$$Y_{\text{mRS before embolisation}} = -0.141 + 0.328X_{\text{Number of treatment stages}} + 1.813X_{\text{cerebral ischaemia}}$$

where Y – mRS before embolisation value, $X_{\text{Number of treatment stages}}$ – Number of treatment stages, $X_{\text{cerebral ischaemia}}$ – Complications (0 – none, 1 – cerebral ischaemia)

With an 1 increase of Number of treatment stages, an 0.328 of mRS before embolisation should be expected, the change of Complications by cerebral ischaemia is associated with an expected increase of mRS before embolisation by 1.813 .

The resulting regression model is characterized by the correlation coefficient $r_{xy} = 0.621$, which corresponds to the Close relationship on the Chaddock scale. The model was statistically significant ($p = 0.008$). The resulting model explains 38.6% of the observed variance of mRS before embolisation.

The dependence of Time of embolisation on quantitative variables was estimated using multiple linear regression. The number of observations was 23.

Table S93 – Analysis of Time of embolisation conditioning on Number of NAEM embolisation steps, Embolisation by other agents, Type of NAEM, Type of catheter

	B	std error	t	p
Intercept	64.227	11.756	5.463	< 0.001*
Number of NAEM embolisation steps	15.798	5.664	2.789	0.014*
Embolisation by other agents: alcohol	12.524	21.472	0.583	0.569
Embolisation by other agents: coils	-5.024	21.472	-0.234	0.818
Embolisation by other agents: none	31.055	11.221	2.768	0.015*
Type of NAEM: Onyx+Squid	-32.675	17.250	-1.894	0.079
Type of NAEM: Onyx	-22.549	9.492	-2.376	0.032*
Type of NAEM: Phill	8.920	20.050	0.445	0.663
Type of catheter: Headway	-36.080	15.053	-2.397	0.031*

* – differences are statistically significant ($p < 0.05$)

The observed association of Time of embolisation with Number of NAEM embolisation steps, Embolisation by other agents, Type of NAEM, Type of catheter is presented by a linear regression equation:

$$Y_{\text{Time of embolisation}} = 64.227 + 15.798X_{\text{Number of NAEM embolisation steps}} + 12.524X_{\text{alcohol}} - 5.024X_{\text{coils}} + 31.055X_{\text{none}} - 32.675X_{\text{Onyx+Squid}} - 22.549X_{\text{Onyx}} + 8.920X_{\text{Phill}} - 36.080X_{\text{Headway}}$$

where Y – Time of embolisation value, $X_{\text{Number of NAEM embolisation steps}}$ – Number of NAEM embolisation steps, X_{alcohol} – Embolisation by other agents (0 – adhesive, 1 – alcohol), X_{coils} – Embolisation by other agents (0 – adhesive, 1 – coils), X_{none} – Embolisation by other agents (0 – adhesive, 1 – none), $X_{\text{Onyx+Squid}}$ – Type of NAEM (0 – Squid, 1 – Onyx+Squid), X_{Onyx} – Type of NAEM (0 – Squid, 1 – Onyx), X_{Phill} – Type of NAEM (0 – Squid, 1 – Phill), X_{Headway} – Type of catheter (0 – Scepter C, XC, 1 – Headway)

With an 1 increase of Number of NAEM embolisation steps, an 15.798 min of Time of embolisation should be expected, the change of Embolisation by other agents by alcohol is associated with an expected increase of Time of embolisation by 12.524 min, the change of Embolisation by other agents by coils is associated with an expected decrease of Time of embolisation by 5.024 min, the change of Embolisation by other agents by none is associated with an expected increase of Time of embolisation by 31.055 min, the change of Type of NAEM by Onyx+Squid is associated with an expected decrease of Time of embolisation by 32.675 min, the change of Type of NAEM by Onyx is associated with an expected decrease of Time of embolisation by 22.549 min, the change of Type of NAEM by Phill is associated with an expected increase of Time of embolisation by 8.920 min, the change of Type of catheter by Headway is associated with an expected decrease of Time of embolisation by 36.080 min.

The resulting regression model is characterized by the correlation coefficient $r_{xy} = 0.847$, which corresponds to the Strong relationship on the Chaddock scale. The model was statistically significant ($p = 0.007$). The resulting model explains 71.8% of the observed variance of Time of embolisation.

The selection of predictors of Type of NAEM did not reveal statistically significant associations.

The selection of predictors of Coils for NAEM did not reveal statistically significant associations.

The selection of predictors of Radicality embolisation did not reveal statistically significant associations.

The selection of predictors of Type of catheter did not reveal statistically significant associations.

The selection of predictors of NAEM volume did not reveal statistically significant associations.

The selection of predictors of Complications did not reveal statistically significant associations.

The dependence of mRS at discharge on quantitative variables was estimated using multiple linear regression. The number of observations was 23.

Table S94 – Analysis of mRS at discharge conditioning on Type of Lesion, Number of treatment stages, Number of NAEM embolisation steps, Open Surgical Interventions, mRS before embolisation, Radicality embolisation, NAEM volume, Complications

	B	std error	t	*p*
Intercept	1.575	0.385	4.088	0.002*
Type of Lesion: Carotid body paraganglioma	-0.997	0.258	-3.860	0.002*
Type of Lesion: Jugular paraganglioma	0.712	0.315	2.263	0.043*
Number of treatment stages	-0.868	0.224	-3.882	0.002*
Number of NAEM embolisation steps	0.587	0.181	3.236	0.007*
Open Surgical Interventions: before embolisation	0.351	0.390	0.899	0.387
Open Surgical Interventions: none	-0.961	0.207	-4.651	< 0.001*
mRS before embolisation	0.318	0.133	2.385	0.034*
Radicality embolisation: subtotal	-1.027	0.315	-3.261	0.007*
NAEM volume	0.083	0.027	3.110	0.009*
Complications: cerebral ischaemia	0.724	0.311	2.324	0.038*

* – differences are statistically significant ($p < 0.05$)

The observed association of mRS at discharge with Type of Lesion, Number of treatment stages, Number of NAEM embolisation steps, Open Surgical Interventions, mRS before embolisation, Radicality embolisation, NAEM volume, Complications is presented by a linear regression equation:

$$Y_{\text{mRS at discharge}} = 1.575 - 0.997X_{\text{Carotid body paraganglioma}} + 0.712X_{\text{Jugular paraganglioma}} - 0.868X_{\text{Number of treatment stages}} + 0.587X_{\text{Number of NAEM embolisation steps}} + 0.351X_{\text{before embolisation}} - 0.961X_{\text{none}} + 0.318X_{\text{mRS before embolisation}} - 1.027X_{\text{subtotal}} + 0.083X_{\text{NAEM volume}} + 0.724X_{\text{cerebral ischaemia}}$$

where Y – mRS at discharge value, $X_{\text{Carotid body paraganglioma}}$ – Type of Lesion (0 – Arteriovenous Malformation of Face, 1 – Carotid body paraganglioma), $X_{\text{Jugular paraganglioma}}$ – Type of Lesion (0 – Arteriovenous Malformation of Face, 1 – Jugular paraganglioma), $X_{\text{Number of treatment stages}}$ – Number of treatment stages, $X_{\text{Number of NAEM embolisation steps}}$ – Number of NAEM embolisation steps, $X_{\text{before embolisation}}$ – Open Surgical Interventions (0 – after embolisation, 1 – before embolisation), X_{none} – Open Surgical Interventions (0 – after embolisation, 1 – none), $X_{\text{mRS before embolisation}}$ – mRS before embolisation, X_{subtotal} – Radicality embolisation (0 – total, 1 – subtotal), $X_{\text{NAEM volume}}$ – NAEM volume (ml), $X_{\text{cerebral ischaemia}}$ – Complications (0 – none, 1 – cerebral ischaemia)

The change of Type of Lesion by Carotid body paraganglioma is associated with an expected decrease of mRS at discharge by 0.997 , the change of Type of Lesion by Jugular paraganglioma is associated with an expected increase of mRS at discharge by 0.712 , With an 1 decrease of Number of treatment stages, an 0.868 of mRS at discharge should be expected, With an 1 increase of Number of NAEM embolisation steps, an 0.587 of mRS at discharge should be expected, the change of Open Surgical Interventions by before embolisation is associated with an expected increase of mRS at discharge by 0.351 , the change of Open Surgical Interventions by none is associated with an expected decrease of mRS at discharge by 0.961 , With an 1 increase of mRS before embolisation, an 0.318 of mRS at discharge should be expected, the change of Radicality embolisation by subtotal is associated with an expected decrease of mRS at discharge by 1.027 , With an 1 ml increase of NAEM volume, an 0.083 of mRS at discharge should be expected, the change of Complications by cerebral ischaemia is associated with an expected increase of mRS at discharge by 0.724 .

The resulting regression model is characterized by the correlation coefficient $r_{xy} = 0.968$, which corresponds to the Functional relationship on the Chaddock scale. The model was statistically significant ($p < 0.001$). The resulting model explains 93.6% of the observed variance of mRS at discharge.
